# RNA‐Binding Protein Hnrnpa1 Triggers Daughter Cardiomyocyte Formation by Promoting Cardiomyocyte Dedifferentiation and Cell Cycle Activity in a Post‐Transcriptional Manner

**DOI:** 10.1002/advs.202402371

**Published:** 2024-11-19

**Authors:** Chuling Li, Yijin Chen, Qiqi Chen, Haoxiang Huang, Michael Hesse, Yilin Zhou, Ming Jin, Yu Liu, Yifei Ruan, Xiang He, Guoquan Wei, Hao Zheng, Senlin Huang, Guojun Chen, Wangjun Liao, Yulin Liao, Yanmei Chen, Jianping Bin

**Affiliations:** ^1^ Department of Cardiology State Key Laboratory of Organ Failure Research Nanfang Hospital Southern Medical University Guangzhou 510515 China; ^2^ Guangdong Provincial Key Laboratory of Cardiac Function and Microcirculation Guangzhou 510515 China; ^3^ Bioland Laboratory (Guangzhou Regenerative Medicine and Health Guangdong Laboratory) Guangzhou 510005 China; ^4^ Institute of Physiology I, Life and Brain Center Medical Faculty University of Bonn 53115 Bonn Germany; ^5^ Department of Oncology Nanfang Hospital Southern Medical University Guangzhou 510515 China

**Keywords:** daughter cardiomyocyte formation, dedifferentiation and cell cycle activity, Hnrnpa1, post‐transcriptional regulation, RNA‐binding protein

## Abstract

Stimulating cardiomyocyte (CM) dedifferentiation and cell cycle activity (DACCA) is essential for triggering daughter CM formation. In addition to transcriptional processes, RNA‐binding proteins (RBPs) are emerging as crucial post‐transcriptional players in regulating CM DACCA. However, whether post‐transcriptional regulation of CM DACCA by RBPs could effectively trigger daughter CM formation remains unknown. By performing integrated bioinformatic analysis of snRNA‐seq data from neonatal and adult hearts, this study identified Hnrnpa1 as a potential RBP regulating CM DACCA. Hnrnpa1 expression decreased significantly during postnatal heart development. With the use of α‐MHC‐H2B‐mCh/CAG‐eGFP‐anillin transgenic mice, Hnrnpa1 overexpression promoted CM DACCA, thereby triggering daughter CM formation and enhancing cardiac repair after myocardial infarction (MI). In contrast, CRISPR/Cas9 technology is used to generate CM‐specific Hnrnpa1 knockout mice. Hnrnpa1 knockout inhibited cardiac regeneration and worsened cardiac function in the neonatal MI model. Nanopore RNA sequencing, RIP assay, IP‐MS, MeRIP‐qPCR, PAR‐CLIP and luciferase reporter experiments showed that Hnrnpa1 induced Mettl3 post‐transcriptional splicing to inhibit m6A‐dependent Pbx1 and E2F1 degradation, thereby increasing Runx1, Ccne1, Cdk2 and Ccnb2 expression to promote CM DACCA. In conclusion, Hnrnpa1 triggered daughter CM formation by promoting CM DACCA in a post‐transcriptional manner, indicating that Hnrnpa1 might serve as a promising target in cardiac repair post‐MI.

## Graphical abstract

RNA‐binding protein Hnrnpa1 induces Mettl3 post‐transcriptional splicing to inhibit m6A‐dependent Pbx1 and E2F1 degradation, thereby increasing Runx1, Ccne1, Cdk2 and Ccnb2 expression to promote cardiomyocyte dedifferentiation and cell cycle activity.

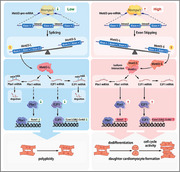

## 1 Introduction

Stimulating cardiac regeneration is a promising approach for promoting cardiac repair in many cardiovascular diseases, such as myocardial infarction (MI) and cardiomyopathy.^[^ ^1^ ^]^ Previous studies have focused primarily on controlling cardiomyocyte (CM) cell cycle reentry to induce cardiac regeneration through the modulation of cell cycle regulators, such as cyclin A2,^[^ ^2^ ^]^ lncDACH1^[^ ^3^ ^]^ and Meis1,^[^ ^4^ ^]^ or signaling pathways, such as the Hippo pathway,^[^ ^5^ ^]^ thyroid hormone signaling^[^ ^6^ ^]^ and Neuregulin1/ErbB4 signaling.^[^ ^7^ ^]^ Although the above regenerative strategies could prolong the postnatal proliferative window of CMs to varying degrees, most of these protocols likewise resulted in the generation of multinucleated CMs rather than daughter CMs.^[^ ^8^, ^9^ ^]^ The successful generation of daughter CMs from preexisting CMs requires efficient progression through the dedifferentiation and cell cycle phases, in which dedifferentiation allows mature adult CMs to enter the preproliferative state by reactivating cardiac embryonic genes and inducing partial disassembly of contractile sarcomeres.^[^ ^10^, ^11^, ^12^ ^]^ Without dedifferentiation, the biophysical constraints exerted by sarcomeres could limit the cytokinesis of adult CMs.^[^ ^13^ ^]^ Thus, coordinated regulation of CM dedifferentiation and cell cycle activity could be a source of inspiration for developing new strategies triggering daughter CM formation.

Currently, coordinated regulation of CM dedifferentiation and cell cycle activity is predominantly achieved by modulating the expression of functional mRNAs or noncoding RNAs (ncRNAs) via transcriptional regulation of precursor RNA formation.^[^ ^10^, ^14^ ^]^ Looking beyond transcriptional regulation,^[^ ^15^ ^]^ post‐transcriptional regulation is another critical player that regulates the expression of functional RNAs.^[^ ^16^, ^17^ ^]^ Targeting post‐transcriptional regulation is economical and efficient, as it typically regulates the location, maturation or stability of preexisting transcripts, which can rapidly alter the functions of preexisting RNAs.^[^ ^18^ ^]^ Accumulating evidence indicates that RNA‐binding proteins (RBPs) play crucial roles in the post‐transcriptional regulation of tissue development and regeneration.^[^ ^19^, ^20^, ^21^ ^]^ Through interactions with protein partners and target transcripts, RBPs control RNA metabolism through processes ranging from pre‐mRNA splicing to mRNA transportation, localization, stability and translation.^[^ ^22^ ^]^ Thus, RBPs contribute to the diversity of the proteome, the stage‐ and tissue‐specific expression of protein isoforms, and the maintenance of protein homeostasis within the cell.^[^ ^20^ ^]^ Certain kinds of RBPs have been found to be master post‐transcriptional regulators in the physiological and pathological conditions of the heart.^[^ ^22^, ^23^ ^]^ RBPs such as RBM20, RBM24, FXR1 and SRSF10 can regulate cardiac embryonic gene expression and CM sarcomere assembly, thereby affecting embryonic heart development.^[^ ^23^ ^]^ Moreover, the RBP LIN28a enhanced CM cell cycle reentry by binding and regulating lncRNA‐H19.^[^ ^24^ ^]^ Therefore, these studies indicated that RBPs have powerful potential to promote CM dedifferentiation and cell cycle activity. However, whether post‐transcriptional regulation of CM dedifferentiation and cell cycle activity by RBPs could be an effective strategy for triggering daughter CM formation remains unknown.

Identification and validation of the key RBPs involved in CM dedifferentiation and cell cycle activity is critical for seeking potential therapeutic targets, but further recognition of the crucial RBP subgroups among numerous candidate genes is difficult. Single‐cell omics could reveal complex and rare cell populations, uncover regulatory relationships among genes and track the trajectories of distinct cell lineages during development.^[^ ^25^, ^26^ ^]^ Because single‐cell omics enable the identification of CM subpopulations and the reconstruction of complex cell‐state transition trajectories, it may be useful in accurately identifying the crucial RBPs regulating CM dedifferentiation and cell cycle activity. Interestingly, a recent study identified Hnrnpa2b1 as the key RBP directing myogenic cell fate decisions by analyzing the single‐cell omics data from muscle stem cells.^[^ ^19^ ^]^ Nevertheless, systemic screening and identification of key RBP functions in CM dedifferentiation and cell cycle activity through single‐cell omics are still lacking. Herein, we performed a comprehensive bioinformatics analysis of single‐nucleus RNA sequencing (snRNA‐seq) data obtained from neonatal and adult hearts and identified Hnrnpa1 as the key RBP involved in CM dedifferentiation and cell cycle activity. We postulated that Hnrnpa1‐mediated post‐transcriptional regulation of CM dedifferentiation and cell cycle activity would be a flexible and effective strategy for triggering daughter CM formation.

In line with this hypothesis, we found that the cardiac‐specific RBP Hnrnpa1 induced Mettl3 post‐transcriptional splicing to promote CM dedifferentiation and cell cycle activity, thereby effectively triggering daughter CM formation and functional recovery post‐MI. Hence, targeting Hnrnpa1‐mediated post‐transcriptional regulation might serve as a promising strategy for inducing daughter CM formation and cardiac regenerative response after injury.

## 2 Results

### 2.1 RNA‐Binding Protein Hnrnpa1 has the Potential to Regulate CM Dedifferentiation and Cell Cycle Activity

**Figure 1:**
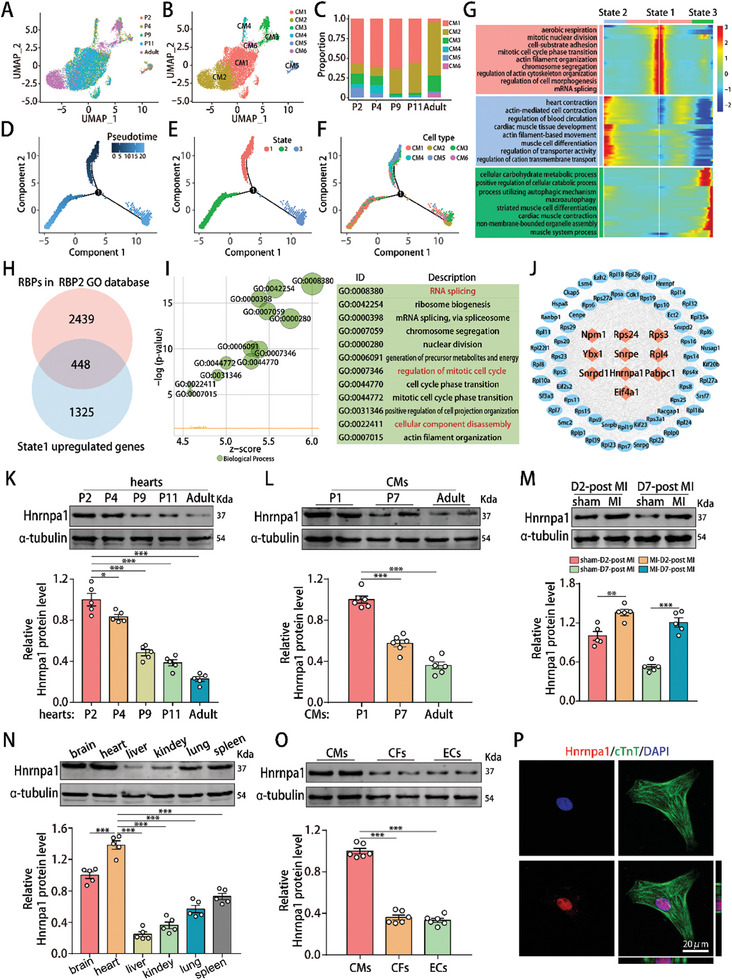
RNA‐binding protein Hnrnpa1 has the potential to regulate CM dedifferentiation and cell cycle activity. A) UMAP visualization of CM clusters colored by time point. B) UMAP visualization of CM clusters colored by identity (n = 13 383). C) The fraction of CM populations in each snRNA‐seq sample. D–F) Pseudotime trajectory of CMs with each origin indicated on the trajectory. G) Heatmap showing the expression of differentially regulated genes during the state 1‐to‐state 3 (right) transition, with the top GO terms for the differentially expressed genes shown on the left. H) Venn diagram showing the number of upregulated RBPs in state 1. I) GO enrichment analysis of upregulated RBPs in state 1. J) Protein‐protein interaction analysis of upregulated RBPs in cluster 3 to identify crucial RBPs. K) Hnrnpa1 protein levels in mouse hearts of different ages. n = 5 mice. L) Hnrnpa1 protein levels in CMs isolated from mouse hearts of different ages. n = 6 cell samples. M) Hnrnpa1 protein levels in neonatal hearts harvested at 2 and 7 days post‐MI. n = 5 mice. N) Hnrnpa1 protein levels in different organs of neonatal mice. n = 5 mice. O) Hnrnpa1 protein levels in isolated CMs, cardiac fibroblasts (CFs) and endothelial cells (ECs). n = 6 cell samples. P) Immunostaining of Hnrnpa1 in CMs. Bar = 20 µm in P. Statistical significance was calculated using one‐way ANOVA in K‐O; *p < 0.05, **p < 0.01, ***p < 0.001.

To identify the crucial RBPs potentially involved in regulating CM dedifferentiation and cell cycle activity, we first conducted an analysis using snRNA‐seq data obtained from neonatal and adult hearts (GSE130699^[^ ^27^ ^]^ and GSE128628^[^ ^28^ ^]^). After filtering the non‐CM nuclei, we successfully captured 13 383 single CM nuclei extracted from all CMs, including mononucleated, binucleated and multinucleated CMs (Figure S1A–C, Supporting Information). By performing dimensionality reduction and clustering, we identified 15 distinct CM subtypes exhibiting high expression of the CM marker Tnnt2 (**Figure** **1**A; Figure S1D–F, Supporting Information). We next integrated them into 6 CM subtypes (Figure 1B) on the basis of specific marker genes identified in previous studies^[^ ^12^, ^27^, ^29^, ^30^, ^31^ ^]^ (Table S1 and Figure S1G, Supporting Information) and gene expression correlation analysis among clusters 1–15 (Figure S1H, Supporting Information). The highly specific marker genes expression and GO enrichment analysis suggested that CM1, CM2, CM3, CM4, CM5, CM6 were immature, mature, adhesion related, proliferating, dedifferentiated and hypertrophic CM cluster, respectively (Figure S1I,J, Supporting Information). Among these CM subtypes, proliferating CM4 cells expressed higher levels of specific cell cycle markers (Mki67 and Cenpp, Figure S1I, Supporting Information) and dedifferentiated CM5 cells expressed higher levels of specific dedifferentiated markers (DAB2 and Runx1, Figure S1I, Supporting Information). Since the specific marker genes of redifferentiated CMs remain to be uncertain, we identified redifferentiated CMs by referring to the related GO enrichment of CM redifferentiation reported in previous studies,^[^ ^12^, ^32^ ^]^ including calcium ion transport,^[^ ^12^ ^]^ cell junction assembly,^[^ ^12^ ^]^ regulation of metal ion transport,^[^ ^32^ ^]^ cytoplasmic translation,^[^ ^32^ ^]^ and oxidative phosphorylation.^[^ ^32^ ^]^ We found that several CM clusters were related to CM redifferentiation and adhesion related CM3 has the strongest redifferentiation potential (Figure S1J, Supporting Information).

After analyzing the ratio of CM1‐6 in different aged hearts, we found that CM4 and CM5 clusters were particularly enriched in newborn mouse hearts, and their total ratio significantly decreased during heart development (Figure 1C). Furthermore, we performed the pseudotime trajectory analysis to infer cell lineage relationships and found that CM trajectories could be divided into three states, in which CM4 and CM5 were primarily concentrated in state 1 (Figure 1D–F; Figure S2A, Supporting Information). By performing RNA velocity analysis of CMs, we found that in state 1, immature CM1 cells might transform into dedifferentiated CM5 cells and dedifferentiated CM5 cells might transform into proliferating CM4 cells (Figure S2B, Supporting Information). In state 2, proliferating CM4 cells might transform into adhesion related CM3 or immature CM1 cells and then adhesion related CM3 cells might transform into immature CM1 cells (Figure S2C, Supporting Information). In state 3, immature CM1 cells might further transform into mature CM2 and hypertrophic CM6 cells (Figure S2D, Supporting Information). Gene Ontology (GO) enrichment analysis revealed that the genes differentially expressed in state 1 were significantly enriched in mitotic cell cycle phase transition, actin filament organization and regulation of actin cytoskeleton organization, indicating that these genes play important roles in CM dedifferentiation and cell cycle activity (Figure 1G).

To identify the potential RBPs that induce CM dedifferentiation and cell cycle activity, we selected 448 RBPs with higher expression in state 1 for further investigation (Figure 1H). GO enrichment analysis revealed that these RBPs were enriched in RNA splicing, nuclear division, regulation of the mitotic cell cycle and cellular component disassembly (Figure 1I). Time‐series cluster analysis showed that the 448 upregulated RBPs in state 1 could be classified into four clusters according to their expression patterns during postnatal heart development (Figure S3A,B, Supporting Information). Since the mRNA expression trends of cluster 3‐RBPs were most similar to the trends in the CM4+CM5 proportion and in the “regulation of mitotic cell cycle” or “cellular component disassembly” score during postnatal heart development (Figure S3C–E, Supporting Information), we chose cluster 3 for further analysis. Analysis of the protein‐protein interaction networks of the RBPs in cluster 3 revealed that 10 genes might be crucial RBPs involved in CM dedifferentiation and cell cycle activity (Figure 1J). qRT‐PCR showed a significant decrease in the expression of the RBPs Npm1, Rps24, Ybx1, Snrpd1 and Hnrnpa1 during heart or CM development (Figure S3F,G, Supporting Information). We also investigated the expression of these RBPs in different types of heart cells and found that the RBPs Npm1, Ybx1 and Hnrnpa1 were highly expressed in CMs (Figure S3H, Supporting Information).

Compared with Npm1 and Ybx1, Hnrnpa1 mRNA expression exhibited a more significant reduction during heart or CM development (Figure S3F,G, Supporting Information). Additionally, CMs have a higher expression of Hnrnpa1 compared with that in CFs and ECs (Figure S3H, Supporting Information). Hence, we focused on the detailed expression profile of Hnrnpa1. Western blotting showed that Hnrnpa1 protein expression decreased significantly during postnatal heart or CM development (Figure 1K,L) and increased partially at 2 and 7 days post‐MI in neonatal hearts (Figure 1M). In addition, Hnrnpa1 was expressed primarily in heart tissues and highly expressed in isolated neonatal mouse CMs (Figure 1N,O). Immunostaining showed that Hnrnpa1 was predominantly located in the nucleus of neonatal mouse CMs (Figure 1P). Therefore, these results indicated that Hnrnpa1 has the potential to regulate CM dedifferentiation and cell cycle activity.

### 2.2 Hnrnpa1 Overexpression Promoted P7 CM Dedifferentiation and Cell Cycle Activity In Vitro

**Figure 2:**
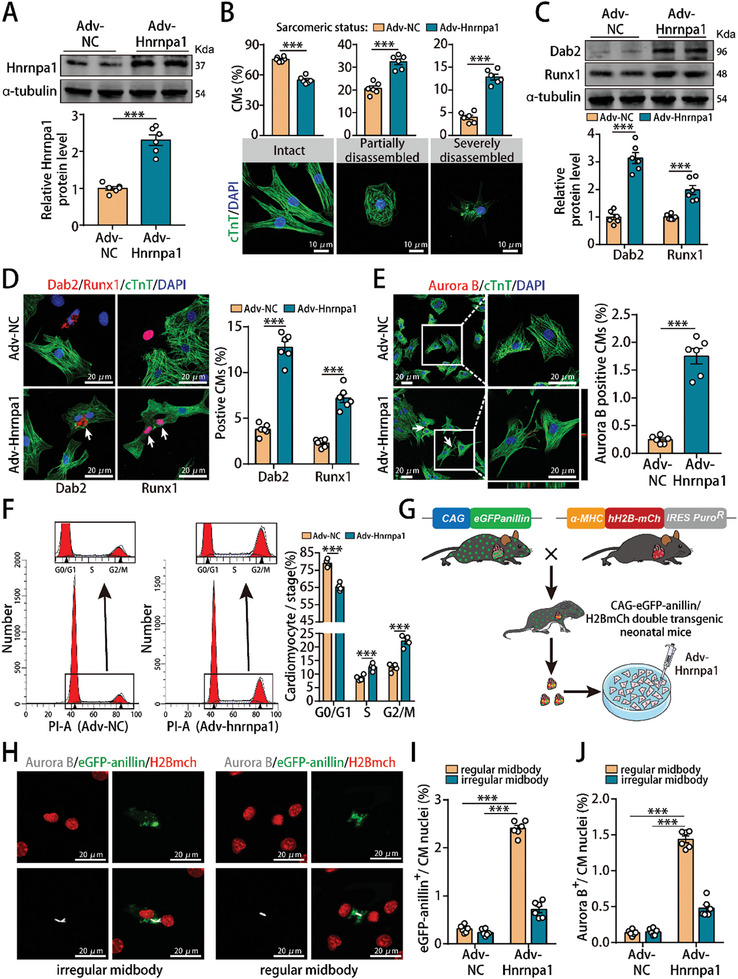
Hnrnpa1 overexpression promoted P7 CM dedifferentiation and cell cycle activity in vitro. A) Hnrnpa1 protein levels in isolated P7 CMs after Hnrnpa1 overexpression. n = 6 cell samples. B) cTnT staining of P7 CMs and quantification of the sarcomeric status after Hnrnpa1 overexpression. Representative images of CMs with intact, partially disassembled and severely disassembled sarcomeres are shown. n = 6 cell samples. C) Runx1 and Dab2 protein levels in P7 CMs after Hnrnpa1 overexpression. n = 6 cell samples. D) Dab2 or Runx1 staining of P7 CMs (1530 CMs from 6 mice in the Adv‐NC group and 1470 CMs from 6 mice in the Adv‐Hnrnpa1 group for Dab2 staining; 968 CMs from 6 mice in the Adv‐NC group and 937 CMs from 6 mice in the Adv‐Hnrnpa1 group for Runx1 staining). Dab2 or Runx1 positive cardiomyocyte was indicated by an arrow. E) Aurora B staining of P7 CMs (2632 CMs from 6 mice in the Adv‐NC group and 1671 CMs from 6 mice in the Adv‐Hnrnpa1 group). F) Detection of cell cycle progression in P7 CMs after Hnrnpa1 overexpression by flow cytometry. n = 4 cell samples. G) Schematic showing the generation of CAG‐eGFP‐anillin/H2BmCh double transgenic mice followed by the isolation of CMs and Adv‐Hnrnpa1 transduction. H–J) Representative image of P7 double transgenic CMs expressing eGFP‐anillin (green) and Aurora B (white) after Hnrnpa1 overexpression and the frequency of regular and irregular midbodies identified by anillin or Aurora B staining (4726 CMs from 6 mice in the Adv‐NC group and 2829 CMs from 6 mice in the Adv‐Hnrnpa1 group). Bars = 10 µm in B, and 20 µm in D‐E and H. Statistical significance was calculated using an unpaired t test in A‐E, and two‐way ANOVA in F and I‐J; *p < 0.05, **p < 0.01, ***p < 0.001.

To determine whether Hnrnpa1 could promote P7 CM dedifferentiation and cell cycle activity, we generated an adenoviral vector to overexpress Hnrnpa1 in isolated P7 CMs (Figure S4A, Supporting Information). Successful transduction of Adv‐Hnrnpa1 resulted in a significant increase in Hnrnpa1 mRNA and protein levels (**Figure** **2**A; Figure S4B, Supporting Information). Hnrnpa1 overexpression increased the number of rounded cells displaying partially and severely disassembled sarcomeric structures (Figure 2B). In addition, Hnrnpa1 overexpression upregulated the mRNA and protein expression of the dedifferentiated markers Dab2 and Runx1 (Figure 2C; Figure S4c, Supporting Information), and significantly increased the proportion of P7 CMs expressing Dab2 and Runx1 (Figure 2D). Immunofluorescence experiments showed that Hnrnpa1 overexpression increased the proportion of CMs expressing Ki67, EdU, pH3 or Aurora B (Figure 2E; Figure S4D–F, Supporting Information) and induced a greater proportion of mononucleated CMs (Figure S4G, Supporting Information). Furthermore, flow cytometry assays revealed that Hnrnpa1 overexpression promoted P7 CM cell cycle activity (Figure 2F). The α‐MHC‐H2B‐mCh/CAG‐eGFP‐anillin system was subsequently used to evaluate whether Hnrnpa1 overexpression could promote CM cytokinesis (Figure 2G; Figure S4H, Supporting Information). Anillin and Aurora B staining showed that Hnrnpa1 overexpression in P7 CMs isolated from α‐MHC‐H2B‐mCh/CAG‐eGFP‐anillin double transgenic mice led to an increase of regular midbodies, suggesting that Hnrnpa1 overexpression promoted daughter CM formation (Figure 2H–J). The growth curve and multiple time point evaluation further showed that Hnrnpa1 overexpression increased the proliferative rate of P7 CMs starting from 32 h post Adv‐Hnrnpa1 infection and became stable thereafter 80 h post Adv‐Hnrnpa1 infection, indicating that Hnrnpa1 overexpression induced the extent of increase in CM number (Figure S5A,B, Supporting Information). Taken together, these data indicated that Hnrnpa1 overexpression promoted P7 CM dedifferentiation and cell cycle activity, thereby triggering daughter CM formation rather than multinucleated CMs.

To investigate the role of Hnrnpa1 in human CMs, we first conducted an analysis using preprocessed and integrated scRNA‐seq data obtained from fetal and adult human hearts (GSE216019^[^ ^33^ ^]^) and successfully captured 8930 CMs after filtering non‐CMs (Figure S6A,B, Supporting Information). Hnrnpa1 exhibited higher expression level in fetal CMs than in adult CMs (Figure S6C,D, Supporting Information). By conducting gene expression correlation analysis, we identified 1511 genes that were positively correlated with Hnrnpa1 (*p*‐value < 0.05 and Pearson correlation index>0.8). These genes were significantly enriched in nuclear division, mitotic cell cycle phase transition and so on (Figure S6E,F, Supporting Information), indicating the potential role of Hnrnpa1 in regulating human CM proliferation. To test the role of Hnrnpa1 in human CMs, we further overexpressed Hnrnpa1 in postmitotic 60‐day‐old human induced pluripotent stem cell‐derived CMs (hiPSC‐CMs), and observed an increase in pH3^+^ or Aurora B^+^ hiPSC‐CMs (Figure S6G,H, Supporting Information). Collectively, these results indicated that Hnrnpa1 overexpression induced cell cycle activation in hiPSC‐CMs.

### 2.3 Hnrnpa1 Overexpression Promoted adult CM Dedifferentiation and Cell Cycle Activity In Vivo

**Figure 3:**
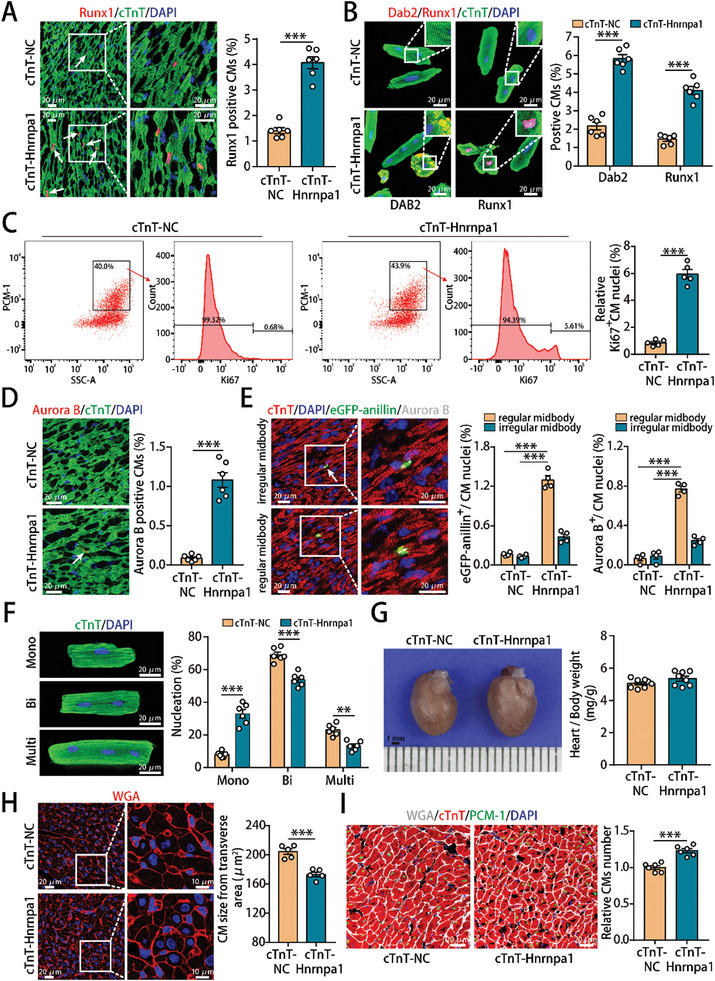
Hnrnpa1 overexpression promoted adult CM dedifferentiation and cell cycle activity in vivo. A) Runx1 staining of adult mouse hearts (2629 CMs from 6 mice in the cTnT‐NC group and 2208 CMs from 6 mice in the cTnT‐Hnrnpa1 group). B) Detection of Dab2^+^ or Runx1^+^ adult CMs isolated from adult mouse hearts (845 CMs from 6 mice in the cTnT‐NC group and 947 CMs from 6 mice in the cTnT‐Hnrnpa1 group for Dab2 staining, 2357 CMs from 6 mice in the cTnT‐NC group and 2417 CMs from 6 mice in the cTnT‐Hnrnpa1 group for Runx1 staining). C) Flow cytometry analysis of Ki67‐positive CM nuclei in adult mouse hearts after Hnrnpa1 overexpression. PCM1 was used to label cardiomyocyte nuclei and Ki67 was used to evaluate cell cycle activity. n = 5 mice. D) Aurora B staining of adult mouse hearts (8002 CMs from 6 mice in the cTnT‐NC group and 4118 CMs from 6 mice in the cTnT‐Hnrnpa1 group). E) Representative image of a CAG‐eGFP‐anillin transgenic adult CM expressing eGFP‐anillin (green) in an asymmetrical or symmetrical midbody (arrow) and Aurora B staining (white) after treatment and the frequencies of regular and irregular midbodies in normal adult hearts (4629 CMs from 4 mice in the cTnT‐NC group and 1867 CMs from 4 mice in the cTnT‐Hnrnpa1 group). F) Detecting the nucleation of adult CMs isolated from adult mouse hearts at 14 days after AAV9 infection (1390 CMs from 6 mice in the cTnT‐NC group and 1112 CMs from 6 mice in the cTnT‐Hnrnpa1 group). G) Heart‐to‐body weight ratios were measured after Hnrnpa1 overexpression. n = 8 mice. H) WGA staining of ventricular sections at 14 days after AAV9 infection (159 CMs from 5 mice in the cTnT‐NC group and 143 CMs from 5 mice in the cTnT‐Hnrnpa1 group). I) Stereological analysis of the CM number in adult mouse hearts after Hnrnpa1 overexpression. n = 6 mice. Bars = 20 µm in A,B, D–F and I, 20 µm (left) and 10 µm (right) in H, and 1 mm in G. Statistical significance was calculated using an unpaired t test in A–D and G–I, and two‐way ANOVA in E‐F; *p < 0.05, **p < 0.01, ***p < 0.001.

To investigate the role of Hnrnpa1 in normal adult hearts, we used an AAV9‐mediated delivery system harboring the Hnrnpa1 gene with a CM‐specific cTnT promoter to overexpress Hnrnpa1 specifically in adult mouse hearts (Figure S7A,B, Supporting Information). Hnrnpa1 mRNA and protein expression increased significantly at 14 days after AAV9 infection (Figure S7C,D, Supporting Information) and the AAV9 delivery system sustained Hnrnpa1 overexpression for at least 10 weeks (Figure S7E, Supporting Information). Runx1 immunostaining showed that Hnrnpa1 overexpression promoted adult CM dedifferentiation (**Figure** **3**A). Consistent with these findings, Hnrnpa1 overexpression also led to a remarkable increase in the percentages of cells expressing Dab2 or Runx1 in adult CMs isolated from AAV9‐injected adult mouse hearts (Figure 3B). In addition, flow cytometry analysis of Ki67‐positive CM nuclei showed that Hnrnpa1 overexpression promoted cell cycle activity in adult CMs (Figure 3C). In vivo and ex vivo immunostaining experiments also showed that Hnrnpa1 overexpression promoted CM cell cycle activity in adult hearts under physiological conditions, as indicated by increases in the proportion of CMs expressing Ki67, pH3 or Aurora B (Figure 3D; Figure S7F–I, Supporting Information). Moreover, CAG‐eGFP‐anillin transgenic mice were used to confirm the proliferative effect triggered by Hnrnpa1, and the results showed that most adult CMs entering cell cycle progression underwent authentic cell division after Hnrnpa1 overexpression (Figure 3E). We also found that Hnrnpa1 overexpression increased the proportion of mononucleated CMs (Figure 3F), had no significant effect on the heart/body weight ratio (Figure 3G), caused limited effect on cardiac function of normal adult mice (Figure S7J, Supporting Information) and led to a reduction in adult CM size (Figure 3H). Moreover, stereological analysis demonstrated that Hnrnpa1 overexpression increased the total number of adult CMs (Figure 3I). Therefore, these results suggested that Hnrnpa1 overexpression triggered daughter CM formation in normal adult hearts by promoting CM dedifferentiation and cell cycle activity.

### 2.4 Hnrnpa1 Overexpression Promoted Cardiac Functional Recovery Post‐MI

**Figure 4:**
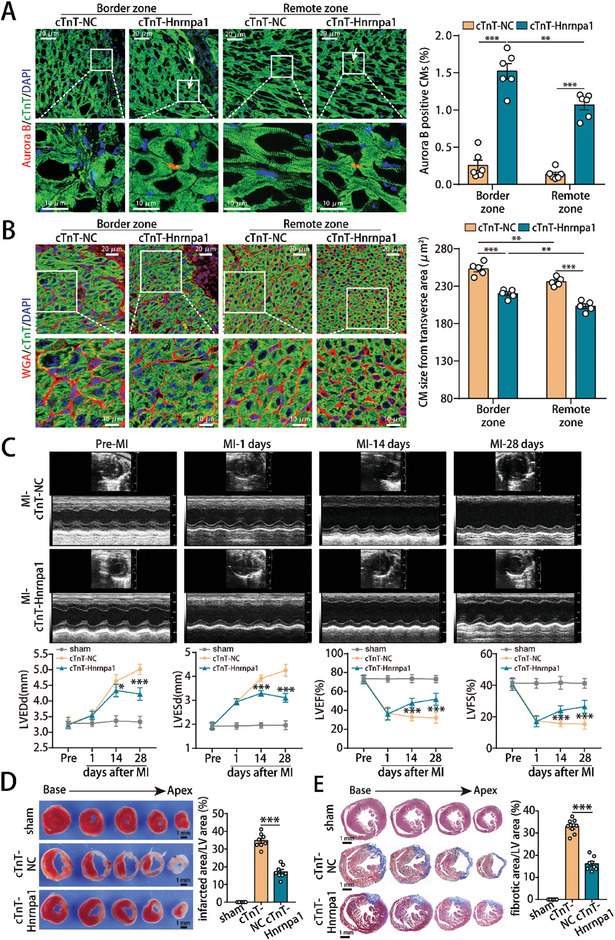
Hnrnpa1 overexpression promoted adult cardiac regeneration and functional recovery after myocardial infarction. A) Aurora B staining of adult mouse hearts at 14 days post‐MI (3674 CMs from the border zone and 5154 CMs from the remote zone of 6 mice in the cTnT‐NC group; 3340 CMs from the border zone and 4687 CMs from the remote zone of 6 mice in the cTnT‐Hnrnpa1 group). B) WGA staining of ventricular sections at 14 days post‐MI (145 CMs from the border zone and 173 CMs from the remote zone of 5 mice in the cTnT‐NC group; 160 CMs from the border zone and 178 CMs from the remote zone of 5 mice in the cTnT‐Hnrnpa1 group). C) Evaluation of cardiac function at pre‐MI, 1, 14 and 28 days post‐MI. n = 8 mice. D) Representative images of TTC‐stained heart cross‐sections from adult mice at 28 days post‐MI. n = 8 mice. E) Representative images of Masson's trichrome‐stained heart cross‐sections from adult mice at 28 days post‐MI. n = 8 mice. Bars = 20 µm (upper) and 10 µm (lower) in A,B and 1 mm in D,E. Statistical significance was calculated using two‐way ANOVA in A–C, and one‐way ANOVA in D,E; *p < 0.05, **p < 0.01, ***p < 0.001.

Next, we evaluated the effect of Hnrnpa1 in adult mice after MI (Figure S8A, Supporting Information). qRT‐PCR and western blotting showed that Hnrnpa1 expression increased in adult mouse MI model hearts after AAV9‐cTnT‐Hnrnpa1 infection (Figure S8B,C, Supporting Information). Hnrnpa1 overexpression dramatically increased the proliferative capacity of adult CMs and reduced the CM size in both the border and remote zones of adult mouse MI models (**Figure** **4**A,B; Figure S8D,E, Supporting Information). Additionally, the proliferative rate and CM size of the border zone were greater than those of the remote zone in Hnrnpa1‐overexpressed adult mouse MI models (Figure 4A,B; Figure S8D,E, Supporting Information). We subsequently studied the effect of Hnrnpa1 overexpression on cardiac functional recovery post‐MI. It was found that AAV9 overexpressing Hnrnpa1 significantly improved cardiac function post‐MI (Figure 4C). TTC staining further confirmed that the proportion of viable myocardium was greater in the cTnT‐Hnrnpa1 group than in the cTnT‐NC group (Figure 4D). Masson's trichrome staining revealed that Hnrnpa1 overexpression significantly reduced the size of fibrotic scars (Figure 4E). Taken together, these data confirmed that Hnrnpa1 promoted cardiac regeneration and functional recovery post‐MI.

### 2.5 Hnrnpa1 Depletion Inhibited Neonatal Cardiac Regeneration Post‐MI

**Figure 5:**
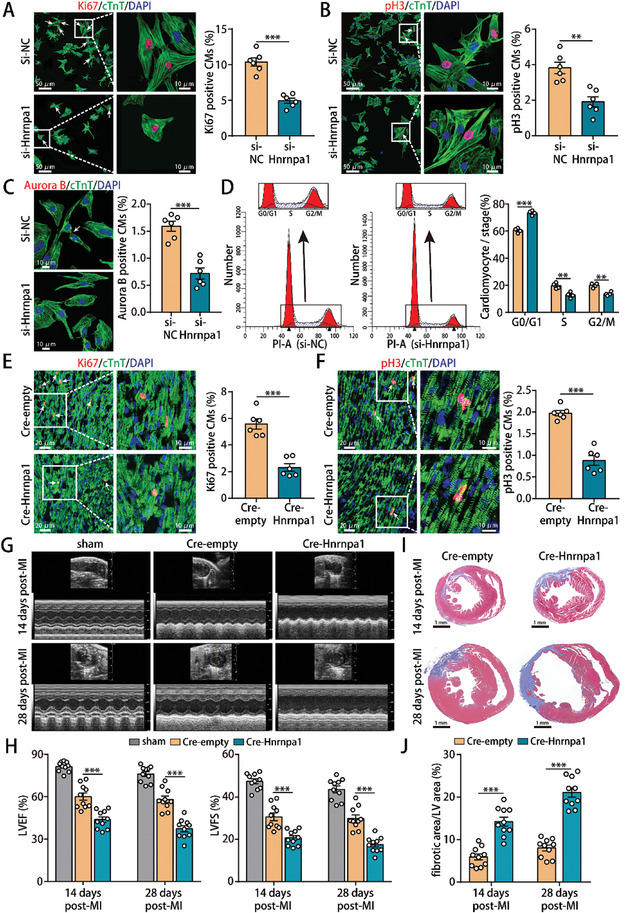
Hnrnpa1 deficiency inhibited neonatal cardiac regeneration. A) Ki67 staining of P1 CMs (916 CMs from 6 mice in the si‐NC group and 573 CMs from 6 mice in the si‐Hnrnpa1 group). B) pH3 staining of P1 CMs (974 CMs from 6 mice in the si‐NC group and 739 CMs from 6 mice in the si‐Hnrnpa1 group). C) Aurora B staining of P1 CMs (2809 CMs from 6 mice in the si‐NC group and 1126 CMs from 6 mice in the si‐Hnrnpa1 group). D) Detection of cell cycle progression in P1 CMs after Hnrnpa1 knockdown by flow cytometry. n = 4 cell samples. E) Ki67 staining of neonatal mouse hearts at 14 days post‐MI (909 CMs from 6 mice in the Cre‐NC group and 760 CMs from 6 mice in the Cre‐Hnrnpa1 group). F) pH3 staining of neonatal mouse hearts at 14 days post‐MI (1475 CMs from 6 mice in the Cre‐NC group and 1877 CMs from 6 mice in the Cre‐Hnrnpa1 group). G,H) Evaluation of cardiac function in neonatal mice at 14 and 28 days post‐MI. n = 10 mice. I,J) Representative images of Masson's trichrome‐stained heart section from neonatal mice at 14 and 28 days post‐MI. n = 10 mice. Bars = 50 µm (left) and 10 µm (right) in A,B, 10 µm in C, 20 µm (left) and 10 µm (right) in E,F, and 1 mm in I. Statistical significance was calculated using an unpaired t test in A–C, E,F and J, two‐way ANOVA in D, and one‐way ANOVA in H; *p < 0.05, **p < 0.01, ***p < 0.001.

Next, we sought to determine the role of Hnrnpa1 in isolated P1 CMs and neonatal mouse hearts. After comparing the efficacy of siRNAs targeting Hnrnpa1, we observed that si‐Hnrnpa1‐3 reduced Hnrnpa1 expression more efficiently (Figure S9A,B, Supporting Information). Hnrnpa1 deficiency resulted in a decrease in the proportion of P1 CMs expressing Ki67, pH3 and Aurora B (**Figure** **5**A–C). In addition, flow cytometry analysis further revealed that Hnrnpa1 deficiency resulted in the accumulation of P1 CMs in the G0/G1 phase (Figure 5D). To assess the in vivo effect of Hnrnpa1 depletion, we constructed CM‐specific Hnrnpa1 knockout mice by injecting Adv‐sgRNA (Hnrnpa1) into neonatal mouse hearts with myocardial Cas9‐tdTomato expression (Figure S10A, Supporting Information). Successful transduction of the Adv‐sgRNA (Hnrnpa1) vector resulted in a significant decrease in Hnrnpa1 expression (Figure S10B–D, Supporting Information). CM‐specific Hnrnpa1 depletion led to dilation of the left ventricle and thinning of the interventricular septum and free walls, thereby reducing the LVEF and LVFS in normal neonatal mouse hearts (Figure S10E,F, Supporting Information). We next evaluated the role of Hnrnpa1 in neonatal mouse MI models (Figure S11A–D, Supporting Information). CM‐specific Hnrnpa1 depletion impeded CM proliferation (Figure 5E,F), impaired cardiac functional recovery (Figure 5G,H) and increased the degree of cardiac fibrosis (Figure 5I,J) in neonatal mouse hearts 14 and 28 days after MI. These responses were consistent with a lower survival rate in neonatal mouse MI models with Hnrnpa1 depletion (Figure S11E, Supporting Information).

### 2.6 Hnrnpa1‐Mediated Post‐Transcriptional Splicing was Critical for CM Dedifferentiation and Cell Cycle Activity

**Figure 6:**
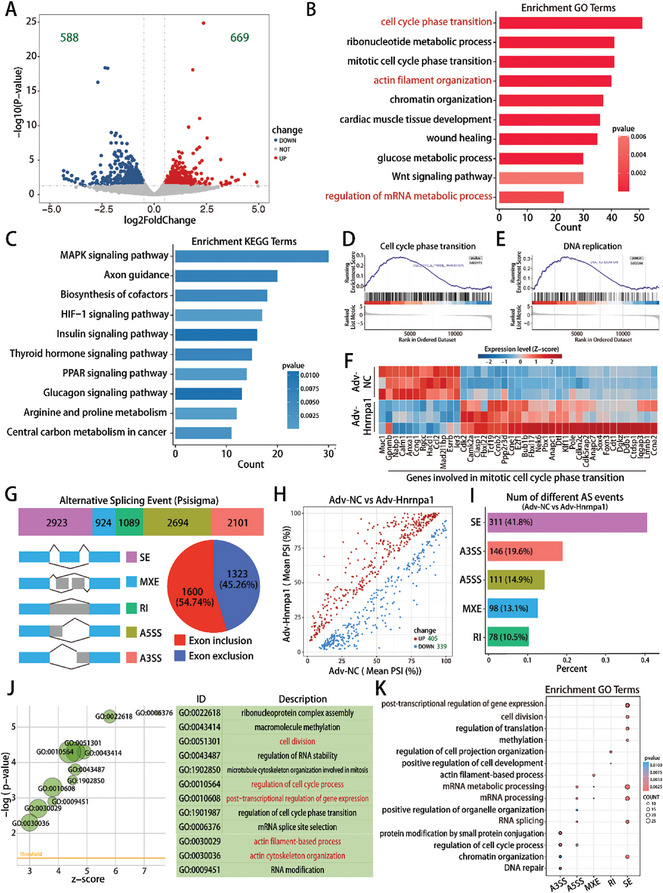
Hnrnpa1‐mediated post‐transcriptional splicing were critical for CM dedifferentiation and cell cycle activity. A) Volcano plot of differentially expressed genes (DEGs) identified by nanopore RNA‐seq of P7 CMs upon Hnrnpa1 overexpression. A |log2FC|>0.5 and a p value < 0.05 were considered to indicate statistical significance. B,C) GO and KEGG pathway enrichment analysis of differentially expressed genes after Hnrnpa1 overexpression. The x‐axis indicates the gene count, and the y‐axis specifies the GO or KEGG terms. D,E) GSEA of cell cycle phase transition and DNA replication enrichment in P7 CMs after Hnrnpa1 overexpression. F) Heatmap displaying the differentially expressed genes involved in the mitotic cell cycle phase transition. G) Hnrnpa1‐regulated total AS events in P7 CMs. AS events are composed of 5 patterns: skipped exon (SE), mutually exclusive exon (MXE), retained intron (RI), alternative 5′ splice site (A5SS) and alternative 3′ splice site (A3SS). Pie chart depicting different types of SE. H) Scatter plot of upregulated and downregulated AS events in P7 CMs after Hnrnpa1 overexpression. A Δ|PSI|> 0.05 and a p value < 0.05 were considered to indicate statistical significance. I) Quantification of the ratio about different AS events in P7 CMs after Hnrnpa1 overexpression. J) GO enrichment analysis of the 554 genes that harbored 744 different AS events. K) GO enrichment analysis of the genes that exhibited 5 patterns of AS events. FC indicates the fold change, and PSI indicates the percent spliced in.

To elucidate the molecular mechanisms of Hnrnpa1‐controlled CM dedifferentiation and cell cycle activity, we performed nanopore RNA‐seq on P7 CMs overexpressing Hnrnpa1. The volcano plot displayed significant upregulation of 669 genes and downregulation of 588 genes (**Figure** **6**A and Table S1, Supporting Information). GO enrichment analysis showed that differentially expressed genes were associated with the cell cycle phase transition, ribonucleotide metabolic process, actin filament organization, and regulation of mRNA metabolic process (Figure 6B). Kyoto Encyclopedia of Genes and Genomes (KEGG) enrichment analysis indicated the involvement of these genes in the MAPK signaling pathway, the HIF‐1 signaling pathway, and the insulin signaling pathway (Figure 6C). Gene set enrichment analysis (GSEA) analysis demonstrated that Hnrnpa1 overexpression facilitated cell cycle phase transition, DNA replication, and cardiac myofibril disassembly in P7 CMs (Figure 6D,E; Figure S12A, Supporting Information). Additionally, Hnrnpa1 overexpression increased the expression of several cell cycle‐related genes and dedifferentiated genes, including Ccne1, Ccna2, Ccnb2, CDK2, Runx1 and Dab2 (Figure 6F; Figure S12B,C, Supporting Information).

Correlation analysis showed that the z‐scores of the cellular component disassembly and the cell cycle process were positively related to the z‐score of the regulation of RNA splicing (Figure S12D,E, Supporting Information), indicating that post‐transcriptional splicing is an important regulatory mechanism in Hnrnpa1‐mediated CM dedifferentiation and cell cycle activity. Hence, we next investigated Hnrnpa1‐mediated post‐transcriptional splicing events. We identified 9731 AS events induced by Hnrnpa1, including 2923 skipped exons (SE, 54.74% exon inclusion and 45.26% exon exclusion), 924 mutually exclusive exons (MXE), 1089 retained introns (RI), 2694 alternative 5′ splice sites (A5SS) and 2101 alternative 3′ splice sites (A3SS) (Figure 6G and Table S2, Supporting Information). Subsequent analysis identified 744 AS events with significant changes and SE as the most frequent AS events in P7 CMs overexpressing Hnrnpa1 (Figure 6H,I and Table S3, Supporting Information). GO enrichment analysis of the 554 genes harboring 744 different AS events revealed that Hnrnpa1 regulated genes involved in macromolecule methylation, cell division, post‐transcriptional regulation of gene expression and actin filament‐based processes, which was consistent with the transcriptional data (Figure 6J,K). Collectively, these data indicated that Hnrnpa1‐mediated post‐transcriptional splicing was critical for CM dedifferentiation and cell cycle activity.

### 2.7 Hnrnpa1 Promoted CM Dedifferentiation and Cell Cycle Activity by Inducing Mettl3 Post‐Transcriptional Splicing

**Figure 7:**
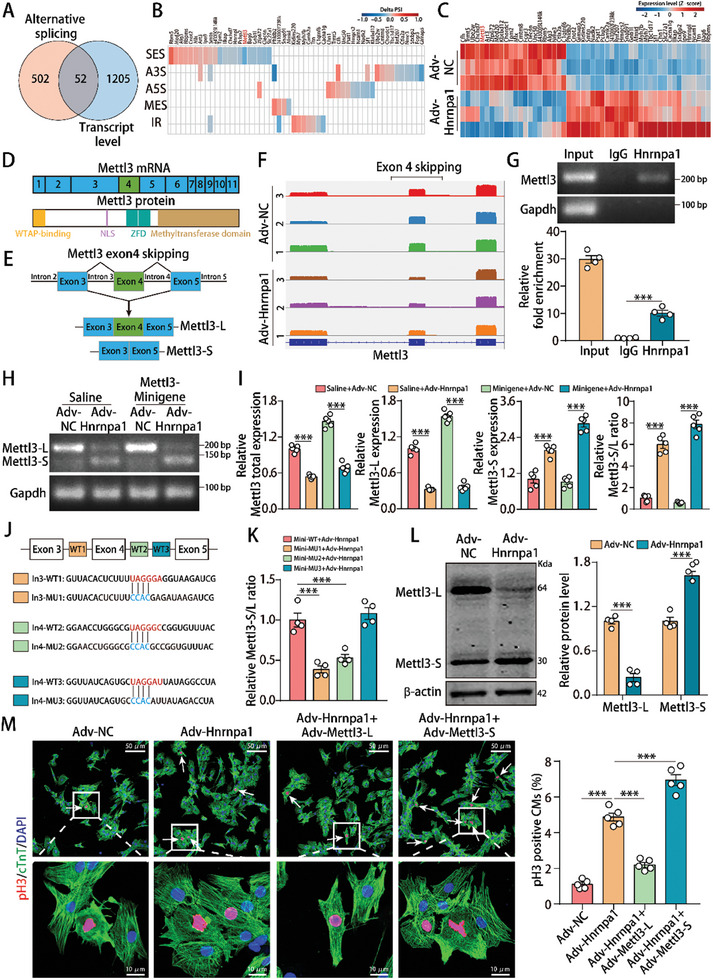
Hnrnpa1 promoted CM dedifferentiation and cell cycle activity by inducing Mettl3 alternative splicing. A) Venn diagram analysis of Hnrnpa1‐regulated genes based on AS events and transcription levels. B) Detailed splicing event information of 52 shared genes in Hnrnpa1‐overexpressed P7 CMs. C) Detailed transcriptional information for 52 shared genes in Hnrnpa1‐overexpressed P7 CMs. D) Schematic exon composition of Mettl3 mRNA and the corresponding schematic domain structure of the Mettl3 protein. The Mettl3 gene has a total of 11 exons, of which exons 4 and 5 contain zinc finger domains. E) AS mode of the Mettl3 pre‐mRNA. Mettl3‐L was produced with exon 4 retention, while Mettl3‐S was produced with exon 4 skipping. F) Sashimi plots illustrating the results of nanopore RNA‐seq for the exon 3‐to‐exon 5 sequence of Mettl3 in Hnrnpa1‐overexpressing P7 CMs. G) Binding of the Mettl3 pre‐mRNA to Hnrnpa1 protein was detected by an RNA immunoprecipitation assay in P7 CMs. n = 4 cell samples. H) Agarose gel electrophoresis was used to assess the expression of Mettl3‐L and Mettl3‐S in P7 CMs after Mettl3 minigene and Hnrnpa1 interference. I) qRT‐PCR was used to determine the mRNA levels of total Mettl3, Mettl3‐L and Mettl3‐S in P7 CMs after Mettl3 minigene and Hnrnpa1 interference. n = 5 cell samples. J) The RNA fragment sequences (wild‐type and mutant) used in the RNA pulldown assay and its position in the Mettl3 pre‐mRNA. The red font represents potential sites, and the blue font represents mutated sites. K) A mutated minigene transfection assay was used to determine the relative Mettl3‐S/L ratio after WT1, WT2 or WT3‐segment mutation. n = 4 cell samples. L) Mettl3‐L and Mettl3‐S protein levels in P7 CMs after Hnrnpa1 overexpression. n = 4 cell samples. M) pH3 staining in P7 CMs after Hnrnpa1, Mettl3‐L and Mettl3‐S interference (940 CMs of 5 mice in the Adv‐NC group, 1113 CMs of 5 mice in the Adv‐Hnrnpa1 group, 1194 CMs of 5 mice in the Adv‐Hnrnpa1+Adv‐Mettl3‐L group and 1123 CMs of 5 mice in the Adv‐Hnrnpa1+Adv‐Mettl3‐S group). Bars = 50 µm (upper) and 10 µm (lower) in M. Statistical significance was calculated using an unpaired t test in L and one‐way ANOVA in G, I, K and M; *p < 0.05, **p < 0.01, ***p < 0.001.

To uncover downstream targets responsible for Hnrnpa1‐controlled CM dedifferentiation and cell cycle activity, we compared Hnrnpa1‐controlled post‐transcriptional splicing events with Hnrnpa1‐regulated differentially expressed genes and identified 52 common genes (**Figure** **7**A). Among these common genes (Figure 7B,C), Mettl3 has been reported to be an important regulator of cardiac regeneration,^[^ ^16^, ^34^, ^35^, ^36^ ^]^ leading to the hypothesis that Hnrnpa1 controls CM dedifferentiation and cell cycle activity by regulating Mettl3 alternative splicing. Mettl3 mRNA consists of 11 exons, and the encoded protein contains a zinc finger domain recognizing targeted genes through direct binding of single‐stranded RNAs and the MT‐A70 methyltransferase domain that mediates cellular m6A deposition (Figure 7D). Analysis of the nanopore RNA‐seq data revealed that exon 4 of full‐length Mettl3 (Mettl3‐L) could be skipped to generate a short isoform (Mettl3‐S) (Figure 7E,F) and qRT‐PCR experiment suggested that Mettl3‐L and Mettl3‐S mRNA have similar stability (Figure S13A, Supporting Information).

RIP assays showed that Hnrnpa1 could directly bind to Mettl3 pre‐mRNA (Figure 7G). We next constructed a minigene fragment spanning exons 3 to 5 to further investigate whether Hnrnpa1 regulates Mettl3 splicing by directly binding to the exon 3‐to‐exon 5 region of Mettl3 pre‐mRNA (Figure S13B, Supporting Information). Semiquantitative RT‐PCR and qPCR revealed a significant increase in the ratio of Mettl3‐S to Mettl3‐L in Hnrnpa1‐overexpressing CMs compared with those in the control CMs (Figure 7H,I). Using the RNA‐binding protein site prediction online server RBPsuite (http://www.csbio.sjtu.edu.cn/bioinf/RBPsuite/),^[^ ^37^ ^]^ we identified three potential Hnrnpa1‐binding sites in the region near exon 4 of the Mettl3 pre‐mRNA (Figure S13C–E, Supporting Information). Subsequently, we performed site‐directed mutagenesis of the potential sequences in the original Mettl3‐minigene plasmid vector and observed a significant reduction in the ratio of Mettl3‐S to Mettl3‐L after mutation of WT1 or WT2 (Figure 7J,K), indicating the importance of WT1 and WT2 in Hnrnpa1‐mediated Mettl3 splicing. In addition, western blotting experiments showed that Hnrnpa1 overexpression reduced the protein product level of Mettl3‐L and increased the protein product level of Mettl3‐S (Figure 7L).

Since that Hnrnpa1 could alter Mettl3 splicing and also reduce its total mRNA expression, we performed rescue experiments to investigate which one is the major downstream for Hnrnpa1's function. Ki67 immunostaining showed that knocking down total Mettl3 mRNA had little effect on abrogating the decreased CM proliferation rate induced by Hnrnpa1 deficiency, indicating that total Mettl3 mRNA expression has a limited effect on Hnrnpa1‐mediated CM function (Figure S14A, Supporting Information). We next evaluated the role of Mettl3 splicing for Hnrnpa1's function. Runx1 and pH3 immunostaining showed that Mettl3‐L inhibited CM dedifferentiation and cell cycle activity, whereas Mettl3‐S overexpression promoted CM dedifferentiation and cell cycle activity (Figure S15A,B, Supporting Information). Moreover, Mettl3‐L abrogated the upregulated rates of CM disassembly and proliferation induced by Hnrnpa1 overexpression, while Mettl3‐S increased dedifferentiation and cell cycle activation in Hnrnpa1‐overexpressed CMs (Figure 7M; Figure S16A, Supporting Information). Taken together, these findings indicated that exon 4 exclusion of Mettl3 is the major downstream of Hnrnpa1‐controlled CM dedifferentiation and cell cycle activity.

### 2.8 Hnrnpa1‐Induced Mettl3 Post‐Transcriptional Splicing Accelerated Pbx1‐Mediated Dedifferentiation and E2f1‐Mediated Cell Cycle Progression in CMs

**Figure 8:**
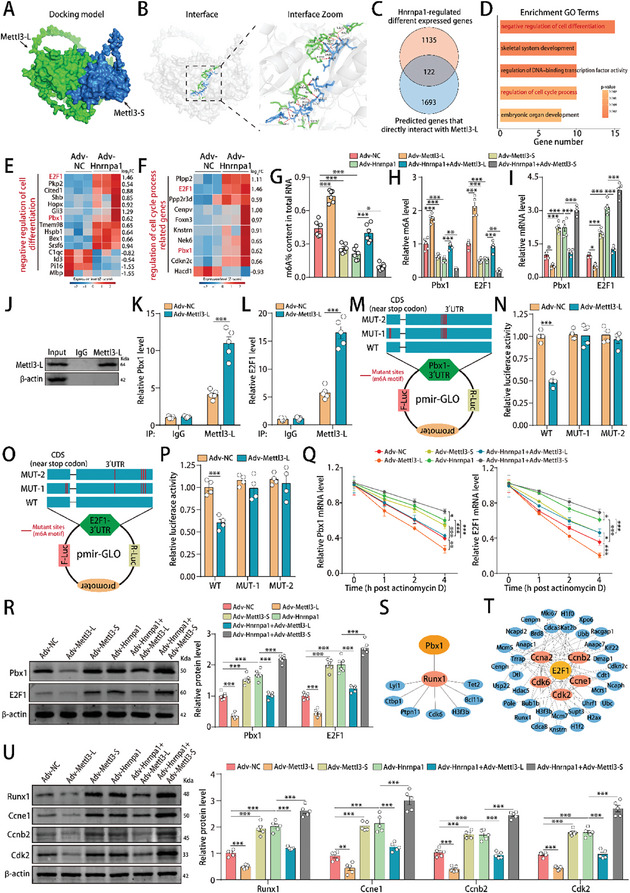
Hnrnpa1‐induced Mettl3 post‐transcriptional splicing accelerated the Pbx1‐mediated dedifferentiation and E2F1‐mediated cell cycle progression in CMs. A) Crystal structure of the Mettl3‐L and Mettl3‐S complexes. Mettl3‐L is depicted in green surface representation, whereas Mettl3‐S is depicted in blue surface representation. B) Crystal structure of the Mettl3‐L and Mettl3‐S complexes and the magnified images of their interaction interface region. The green and blue sticks represent the interacting residues of Mettl3‐L and Mettl3‐S, respectively. Interactions are marked with red dashes. C) Venn diagram analyzing the downstream targets of the Hnrnpa1/Mettl3‐L axis by integrating Hnrnpa1‐regulated differentially expressed genes with predicted genes directly interacting with Mettl3‐L. Genes with a z‐score>1 were identified as potential targets that directly interact with Mettl3‐L on the RNAct website (http://rnact.crg.eu/). D) GO enrichment analysis of the 122 downstream targets of the Hnrnpa1/Mettl3‐L axis. The x‐axis indicates the gene number, and the y‐axis specifies the GO terms. E,F) Heatmap displaying the 122 downstream targets of the Hnrnpa1/Mettl3‐L axis involved in the negative regulation of cell differentiation and the regulation of cell cycle progression. G) The m6A modification of total RNA in P7 CMs after Hnrnpa1, Mettl3‐L and Mettl3‐S interference. n = 6 cell samples. H) MeRIP‐qPCR was used to detect the m6A enrichment of E2F1 and Pbx1 mRNAs in P7 CMs after Hnrnpa1, Mettl3‐L and Mettl3‐S interference. n = 5 cell samples. I) E2F1 and Pbx1 mRNA levels in P7 CMs after Hnrnpa1, Mettl3‐L and Mettl3‐S interference. n = 5 cell samples. J) The Mettl3‐L protein was immunoprecipitated from UV‐cross‐linked P7 CM lysates, and western blot analysis of Mettl3‐L protein levels in the anti‐IgG and anti‐Mettl3‐L groups. K,L) qRT‐PCR analysis of Pbx1 and E2F1 mRNA levels in the anti‐IgG and anti‐Mettl3‐L groups. n = 5 cell samples. M–P) Three Pbx1 or E2F1 plasmids were constructed by inserting the corresponding cDNAs into pmir‐GLO luciferase reporters, and a luciferase assay was used to determine the activities of these pmir‐GLO reporters in P7 CMs after Mettl3‐L overexpression. n = 4 cell samples. The WT reporter included the full‐length 3′UTR and a partial CDS sequence near the stop codon of Pbx1 or E2F1 with intact m6A sites, while the mutant reporters included some A‐C mutations in m6A consensus motifs. Q) Pbx1 and E2F1 mRNA levels in P7 CMs at different time points after Hnrnpa1, Mettl3‐L and Mettl3‐S interference. Actinomycin D was used to block RNA synthesis. n = 5 cell samples. R) Pbx1 and E2F1 protein levels in P7 CMs after Hnrnpa1, Mettl3‐L and Mettl3‐S interference. n = 6 cell samples. S,T) Protein‐protein interaction analysis of the relationships of Pbx1 or E2F1 with Hnrnpa1‐regulated differentially expressed genes. U) Runx1, Ccne1, Cdk2 and Ccnb2 protein levels in P7 CMs after Hnrnpa1, Mettl3‐L and Mettl3‐S interference. n = 5 cell samples. Statistical significance was calculated using one‐way ANOVA in G‐I, R and U, an unpaired t test in K, L, N and P, and two‐way ANOVA in Q; *p < 0.05, **p<0.01, ***p < 0.001.

To elucidate the mechanism underlying the opposite roles of Mettl3‐L and Mettl3‐S in Hnrnpa1‐mediated CM dedifferentiation and cell cycle activity, we first used the catRAPID algorithm^[^ ^38^ ^]^ to predict RNAs that could directly interact with the Mettl3‐L or Mettl3‐S protein (Table S4, Supporting Information). We found that no RNAs could directly interact with Mettl3‐S, indicating that Mettl3‐S might regulate genes expression in an indirect manner (Table S4, Supporting Information). After performing the molecular docking experiment, we found that the Mettl3‐S protein has potential to interact with Mettl3‐L and inhibit its methyltransferase function (**Figure** **8**A,B). To test this postulation, we next performed the immunoprecipitation‐mass spectrometry (IP‐MS) experiments in P7 CMs expressing the Flag‐Mettl3‐S protein to identify the interacting proteins of Mettl3‐S (Figure S17A, Supporting Information), and 42 Mettl3‐S‐interacting proteins were uncovered by IP‐MS (Figure S17B and Table S5, Supporting Information). GO enrichment analysis of the 42 Mettl3‐S‐interacting proteins showed that these genes were involved in mRNA processing, methylation and so on (Figure S17C, Supporting Information). After performing the protein‐protein interaction networks of the above 42 proteins (Figure S17D, Supporting Information), we identified 10 core proteins (Figure S17E, Supporting Information) and found that Mettl3‐L was one of the essential proteins binding to Mettl3‐S (Figure S17F, Supporting Information). Co‐IP experiments in isolated P7 CMs transfected with Adv‐Flag‐Mettl3‐S and Adv‐HA‐Mettl3‐L further demonstrated an interaction between Mettl3‐S and Mettl3‐L (Figure S17G, Supporting Information). Additionally, the rescue experiment showed that Mettl3‐S overexpression abrogated the methyltransferase activity of Mettl3‐L in isolated P7 CMs (Figure S17H, Supporting Information). Therefore, these results suggested that Mettl3‐S could interact with Mettl3‐L and act as an antagonist to inhibit the methyltransferase activity of Mettl3‐L.

To elucidate the downstream process by which Hnrnpa1‐induced Mettl3 post‐transcriptional splicing promoted CM dedifferentiation and cell cycle activity, we next integrated Hnrnpa1‐regulated differentially expressed mRNAs with RNAs predicted to directly interact with the Mettl3‐L protein and identified 122 common genes (Figure 8C). GO enrichment analysis showed that these genes were associated with the negative regulation of cell differentiation and the regulation of cell cycle process (Figure 8D). After comparing genes involved in the negative regulation of cell differentiation and genes involved in the regulation of cell cycle processes, we identified 2 common genes: Pbx1 and E2F1 (Figure 8E,F; Figure S18A, Supporting Information). By using a colorimetric m6A quantification strategy, we found that Hnrnpa1 overexpression reduced m6A modifications of total RNA in P7 CMs, which was counteracted by Mettl3‐L and enhanced by Mettl3‐S (Figure 8G). Furthermore, the m6A modifications and mRNA levels of Pbx1 and E2F1 significantly changed after Hnrnpa1, Mettl3‐L and Mettl3‐S interference, suggesting that Hnrnpa1‐induced Mettl3 post‐transcriptional splicing promoted CM differentiation and cell cycle activity by regulating Pbx1 and E2F1 mRNAs in an m6A‐related manner (Figure 8H,I). PAR‐CLIP experiments confirmed the direct interaction between the Mettl3‐L protein and Pbx1 mRNA or E2F1 mRNA (Figure 8J–L). By employing a sequence‐based m6A modification site predictor (http://www.cuilab.cn/sramp),^[^ ^39^ ^]^ we identified several potential m6A modification sites within the Pbx1 and E2F1 transcripts (Figure S18B,C, Supporting Information). Mettl3‐L overexpression increased the m6A level in the CDS and 3′UTR‐2 regions of Pbx1, and in the CDS‐2 and 3′UTR regions of E2F1 (Figure S18D,E, Supporting Information). We next performed luciferase reporter assays to evaluate the m6A modification of Pbx1 and E2F1, and found that the luciferase activities of Pbx1 and E2F1 were reduced in the WT group after Mettl3‐L overexpression, whereas the luciferase activity in the MUT group was significantly resistant to Mettl3‐L overexpression (Figure 8M–P). Rescue experiments demonstrated that Hnrnpa1 overexpression inhibited the degradation of Pbx1 and E2F1 mRNAs, and increased Pbx1 and E2F1 protein levels, which were counteracted by Mettl3‐L and enhanced by Mettl3‐S (Figure 8Q,R). Additionally, protein‐protein interaction analysis revealed 1 core Pbx1‐regulated dedifferentiation gene and 5 core E2F1‐regulated cell cycle genes in Hnrnpa1‐overexpressing P7 CMs (Figure 8S,T), and rescue experiments showed that Hnrnpa1‐induced Mettl3 post‐transcriptional splicing upregulated the mRNA and protein levels of dedifferentiation and cell cycle genes, such as Runx1, Ccne1, Cdk2, and Ccnb2 (Figure 8U; Figure S18F,G, Supporting Information) without dramatically affecting their m6A levels (Figure S18H, Supporting Information). Therefore, these data indicated that Hnrnpa1 promoted CM dedifferentiation and cell cycle activity by inducing Mettl3 post‐transcriptional splicing to stabilize Pbx1 and E2F1 mRNAs in an m6A‐related manner.

## 3 Discussion

In the current study, our major finding was that Hnrnpa1‐mediated post‐transcriptional splicing played essential roles in the coordinated regulation of CM dedifferentiation and cell cycle activity, which would be an effective strategy for triggering daughter CM formation. The core evidence supporting our findings is as follows: 1) We novelly identified Hnrnpa1 as a crucial post‐transcriptional regulator that promotes CM dedifferentiation and cell cycle activity by analyzing the snRNA‐seq data obtained from neonatal and adult hearts. 2) Gain‐ and loss‐of‐function assays confirmed the important roles of Hnrnpa1 in CM dedifferentiation and cell cycle activity, daughter CM formation and cardiac repair following MI. Another important finding was that we explored the full chain mechanism underlying Hnrnpa1‐mediated CM dedifferentiation and cell cycle activity, in which the roles of Mettl3 post‐transcriptional splicing, and m6A modifications of Pbx1 and E2F1 were newly elucidated in the cardiac regeneration field.

Our findings revealed the essential role of Hnrnpa1 in regulating daughter CM formation by post‐transcriptionally controlling CM dedifferentiation and cell cycle activity. We applied multiple strategies to evaluate the functions of Hnrnpa1 in CMs. First, the comprehensive and strict analysis of the snRNA‐seq data emphasized the potential of Hnrnpa1 for regulating CM dedifferentiation and cell cycle activity. Next, calculation of the CM sarcomeric status and analysis of Runx1 and Dab2 expression confirmed that Hnrnpa1 induced CM dedifferentiation. Along with CM dedifferentiation, immunostaining of cell cycle markers and flow cytometry assays demonstrated that Hnrnpa1 overexpression promoted CM cell cycle activity, which is consistent with the suggestion that Hnrnpa1 functions as a proto‐oncogene to promote cell proliferation and tumorigenesis.^[^ ^40^ ^]^ These Hnrnpa1‐induced beneficial effects indicated the crucial role of Hnrnpa1 in daughter CM formation, making us further evaluate the cytokinetic effects induced by Hnrnpa1. We then used the α‐MHC‐H2B‐mCh/CAG‐eGFP‐anillin system to avoid false positive cytokinesis events,^[^ ^41^, ^42^, ^43^ ^]^ and observed that ≈75% of CMs reentering cell cycle progression underwent authentic cell division after Hnrnpa1 overexpression. In addition, nucleation assays showed that Hnrnpa1 decreased the percentage of binucleated or multinucleated CMs by nearly 25%, which was proven to be a reliable readout of CM cytokinesis. Moreover, ventricular stereological analysis showed an ≈1.23‐fold increase in the number of CMs after Hnrnpa1 overexpression. These findings demonstrated that Hnrnpa1, a dual functional component regulating CM dedifferentiation and cell cycle activity, effectively triggers CM cytokinesis rather than multinucleation. Theoretically, the CM size of proliferating CMs tend to be smaller than mature CMs.^[^ ^44^ ^]^ We observed that the averaged CM size of the border zone was greater than that of the remote zone in both the control and Hnrnpa1‐overexpressed group, which were similar with other studies.^[^ ^45^, ^46^ ^]^ The possible reasons might be that CMs in the border zone suffer from a more severe ischemic reaction and thereby induce a stronger hypertrophic response when compared with that in the remote zone.^[^ ^45^, ^46^ ^]^ Given that Hnrnpa1 acted as a novel regulator of CM proliferation in mice, we also evaluated the role of Hnrnpa1 in human cells. Analysis of scRNA‐seq data indicated the potential of Hnrnpa1 in regulating human CM proliferation, and immunostaining experiments further confirmed that Hnrnpa1 overexpression could induce cell cycle activation in hiPSC‐CMs, indicating that our findings regarding Hnrnpa1 are translatable to human CMs. Recently, studies have revealed that several regulators, such as ERBB2,^[^ ^10^ ^]^ miR‐199^[^ ^43^ ^]^ and lnc‐snhg1,^[^ ^47^ ^]^ play important roles in regulating daughter CM formation. However, the clinical translational value of these regulators is limited by their organ wide expression characteristics.^[^ ^47^, ^48^, ^49^ ^]^ In this study, our results showed that Hnrnpa1 was enriched in the heart and highly expressed in CMs compared with other cardiac cell types. Therefore, Hnrnpa1 might be less likely to cause significant negative effects on other organs and thus represents a more attractive target for triggering daughter CM formation.

We further demonstrated that Hnrnpa1 promoted CM dedifferentiation and cell cycle activity by inducing Mettl3 post‐transcriptional splicing to increase the ratio of Mettl3‐S to Mettl3‐L. Previous studies have reported that different isoforms, including the PKM^[^ ^50^ ^]^ and Periostin^[^ ^51^ ^]^ isoforms, induced by alternative splicing could exert opposite effects on CM function and homeostasis. In the current study, although Hnrnpa1 could alter Mettl3 splicing and reduce its total mRNA expression, we found that Mettl3 splicing was the major downstream for Hnrnpa1's function after performing the rescue experiments. The possible reason might be that the reduction of total mettl3 mRNA might decrease the content of both pro‐proliferative Mettl3 isoforms and anti‐proliferative Mettl3 isoforms at the same time while targeting the single Mettl3 isoform induced by alternative splicing might help to induce a purer reaction on CM proliferation. Moreover, Hnrnpa1 could directly interact with Mettl3 pre‐mRNA and induce exon 4 exclusion in Mettl3, thereby promoting the conversion of the Mettl3‐L isoform to the Mettl3‐S isoform. Interestingly, our functional experiments further showed that Mettl3‐L overexpression inhibited CM dedifferentiation and cell cycle activity, whereas Mettl3‐S overexpression significantly increased the ratio of dedifferentiated or proliferative CMs. Rescue experiments showed that Mettl3‐L overexpression acted as an inhibitor to counteract the positive effects of Hnrnpa1 in CMs, whereas Mettl3‐S overexpression acted as a synergist to enhance Hnrnpa1‐mediated CM function, indicating that the increased ratio of Mettl3‐S to Mettl3‐L was a crucial downstream mechanism of Hnrnpa1‐mediated CM dedifferentiation and cell cycle activity. Notably, previous studies have shown that Mettl3 could exert both inhibitory and promoting effects on cardiac regeneration.^[^ ^16^, ^34^, ^35^, ^36^ ^]^ Some studies have demonstrated that Mettl3 inhibited CM cell cycle activity by promoting m6A modifications of Fgf16^[^ ^16^ ^]^ or miR‐143^[^ ^34^ ^]^ while other studies have reported that Mettl3 induced a cardiac regenerative response by increasing m6A modifications of Psph^[^ ^35^ ^]^ or miR‐17‐3p.^[^ ^36^ ^]^ On the basis of our findings, we postulated that the controversial function of Mettl3 in cardiac regeneration might be attributed to the different transcript isoforms of Mettl3 induced by AS. Lending support to our study, a recent robust research also showed that different Mettl3 isoforms generated by AS could exert various functions in embryonic stem cells.^[^ ^52^ ^]^ Therefore, our findings provide a new mechanism by which AS links Mettl3 with cardiac regenerative capacity, and indicate that the RBP Hnrnpa1 could act as a crucial upstream regulator to control the efficacy of Mettl3 in regulating cardiac regeneration.

We also revealed the mechanism by which Mettl3‐L and Mettl3‐S exert opposite roles in Hnrnpa1‐mediated CM functions. Previous studies have reported that the short isoform of several proteins, including Lin28b^[^ ^53^ ^]^ and Reck,^[^ ^54^ ^]^ could interact with their long isoform to competitively inhibit their function. Similarly, our molecular docking, IP‐MS and rescue experiments showed that Mettl3‐S acted as an antagonist to inhibit the methyltransferase function of Mettl3‐L and thereby indirectly regulated the m6A modifications of downstream genes, which further indicated that AS was a new mechanism involved in the regulation of m6A levels in CMs. Notably, in addition to the core protein Mettl3‐L, we also identified the CM cell cycle regulator Prkar1a^[^ ^55^ ^]^ as an interacting protein of Mettl3‐S. Although Prkar1a belonged to the secondary rather than primary Mettl3‐S‐interacting protein, it would be interesting to evaluate its potential effect on Mettl3‐S‐mediated CM dedifferentiation and cell cycle activity in future studies. We found that the increasing ratio of Mettl3‐S to Mettl3‐L induced by Hnrnpa1 led to a decrease in total m6A levels in CMs, thereby promoting CM dedifferentiation and cell cycle activity, suggesting that total m6A levels are negatively correlated with cardiac regenerative capacity.^[^ ^56^ ^]^ In the current study, we newly identified Pbx1 and E2F1 as crucial m6A targeted genes of Hnrnpa1‐induced Mettl3 post‐transcriptional splicing. M6A modifications could regulate mRNA functions ranging from transportation to stability and nuclear localization.^[^ ^57^ ^]^ Our rescue experiments showed that Mettl3‐L overexpression blocked while Mettl3‐S overexpression enhanced the half‐life extension of Pbx1 and E2F1 mRNA induced by Hnrnpa1, indicating that both Mettl3‐L and Mettl3‐S regulated Pbx1 and E2F1 expression via m6A‐dependent degradation. Lending support to our study, prior research has shown that the m6A modifications of Pbx1^[^ ^58^ ^]^ and E2F1^[^ ^59^ ^]^ affected their stability in cancer cells. Furthermore, we found that the upregulated expression of Pbx1 and E2F1 promoted CM dedifferentiation and cell cycle activity by increasing the expression of Runx1, Ccne1, Cdk2 and Ccnb2. Taken together, our findings showed that Hnrnpa1‐induced Mettl3 post‐transcriptional splicing promoted CM dedifferentiation and cell cycle activity by inhibiting m6A‐dependent Pbx1 and E2F1 degradation to increase the levels of Runx1, Ccne1, Cdk2 and Ccnb2, indicating that m6A‐modified Pbx1 and E2F1 could serve as intervention targets for regulating daughter CM formation.

Our study has several limitations. First, we analyzed both male and female pups without separating the sexes. The possible sex‐specific effects of Hnrnpa1 on MI should be evaluated in future studies. Second, the procedures applied for isolating CMs from 1d‐old, 7d‐old and adult mice in our study were different. Hence, we have established the control group to reduce the bias induced by the isolation procedures.

Our study yielded some interesting and meaningful items worthy of further exploration in the future. First, CM needs to undergo a process of dedifferentiation‐proliferation‐redifferentiation.^[^ ^12^ ^]^ Unlike dedifferentiated and proliferating CMs, the marker genes of redifferentiated CMs remain to be uncertain. In our study, the GO enrichment analysis indicated that adhesion related CM3 has the strongest redifferentiation potential. Hence, it would be worthy to confirm whether CM3 cluster are redifferentiated CMs through potential specific markers, such as adhesion related genes. Besides, in addition to the finding that dedifferentiated CM5 cluster could transform into proliferating CM4 cluster, our RNA velocity analysis also suggested important transformations among CM1, 5, 4, and 3. It would be worth designing more detailed experiments in another future study to investigate whether inducing the transformation of CM1, 5, 4, and 3 has the potential to trigger daughter CM formation. Thirdly, a previous study reported that Hnrnpa1 shares 68% sequence identity with its homolog Hnrnpa2b1,^[^ ^60^ ^]^ it would be interesting to evaluate whether Hnrnpa1 and Hnrnpa2b1 function synergistically in CMs. Finally, given the essential role of RBPs in controlling the biogenesis of circRNAs^[^ ^61^ ^]^ which are important components regulating cardiac regeneration,^[^ ^62^ ^]^ whether Hnrnpa1 promotes CM dedifferentiation and cell cycle activity by modulating circRNA formation worth being further explored.

## 4 Conclusions

The cardiac‐specific RBP Hnrnpa1, which was identified by single‐cell omics, triggered daughter CM formation by promoting CM dedifferentiation and cell cycle activity in a post‐transcriptional manner, indicating that Hnrnpa1 is a promising therapeutic target for heart failure after MI.

## 5 Experimental Section

Healthy C57BL/6J mice were obtained from Southern Medical University. αMHC‐H2B‐mCh and CAG‐eGFP‐anillin mice were provided by Dr. Michael Hesse from the University of Bonn, Germany. The α‐MHC‐Cre mice were provided by Dr. Kunfu Ouyang from Peking University, China, and the Cre‐dependent Cas9 knock‐in mouse model (R26‐CAG‐Cas9/+) was purchased from GemPharmatech, China. All the mice were housed at the specific‐pathogen‐free animal facility on a 12‐hour/12‐hour light/dark cycle with free access to normal food and water. All groups were blinded to genotype until the completion of data analysis. The animal experimental protocols complied with the guidelines of Directive 2010/63/EU of the European Parliament on the protection of animals used for scientific purposes and were approved by the animal ethics committee of Nanfang Hospital, Southern Medical University (Approval no. NFYY‐2018‐1223)

### Establishment of the α‐MHC‐H2B‐mCh/CAG‐eGFP‐Anillin System

α‐MHC‐H2BmCh‐mice were constructed by expressing a fusion protein of human histone 2B and the red fluorescence protein mCherry under the control of the CM specific aMHC promoter, which allowed the unequivocal identification and quantitation of CM nuclei in isolated cells and native tissue slices.^[^ ^41^ ^]^ The CAG‐eGFP‐anillin mice were constructed by expressing eGFP‐anillin, a scaffolding protein of the contractile ring under the control of the CAG promoter, which helps distinguish a typical cell cycle activity from cell division.^[^ ^42^ ^]^ The CAG‐eGFP‐anillin/H2B‐mCh double transgenic mouse line was produced by crossing α‐MHC‐H2BmCh‐mice with CAG‐eGFP‐anillin‐mice, which were maintained on a mixed genetic background derived from CD‐1×C57BL/6Ncr and 129S6/SvEvTac.^[^ ^43^ ^]^ This α‐MHC‐H2B‐mCh/CAG‐eGFP‐anillin double transgenic mouse line enables the unequivocal identification of cardiomyocytes on the basis of mCherry fluorescence and the cell cycle status, as indicated by eGFP‐anillin expression and its subcellular localization. Since the anillin protein is localized in the nucleus during the G1/S/G2 phases of the cell cycle, translocates to the cytoplasm in the M‐phase and then resides in the contractile ring and midbody during cytokinesis, this eGFP‐anillin system enables visualization of cell cytokinesis with high spatiotemporal resolution.

### Statistical Analysis

All the data were presented as the mean ± standard errors of the means (SEMs). Statistical analysis was performed via SPSS 18.0 software. Normality tests were conducted for all continuous variables. Unpaired Student's t‐test was used to determine the significance of differences between two groups. One‐way ANOVA followed by Bonferroni's multiple comparisons test or two‐way ANOVA followed by Sidak's test were employed for analyzing multiple groups. For variables with an abnormal distribution, the nonparametric Mann–Whitney U test was used to compare the two independent groups. Survival analysis was conducted via the Kaplan‐Meier method, and the log‐rank (Mantel‐Cox) test was used to assess differences between survival curves. A p‐value less than 0.05 was considered statistically significant. More detailed descriptions of the materials and methods are provided in the Supplementary Information.

## Conflict of Interest

The authors declare no conflict of interest.

## Author Contributions

C.L.L., Y.J.C. and Q.Q.C. contributed equally to this work. J.P.B., Y.J.C. and C.L.L. conceived and designed the study; C.L.L., Y.J.C., Q.Q.C, L.Y.Z., H.X.H. and Y.L. performed the in vivo experiments; Y.J.C., C.L.L., Q.Q.C, M.J. and Y.F.R. performed the in vitro experiments; M.H. provided the α‐MHC‐H2B‐mCh/CAG‐eGFP‐anillin double transgenic mouse line; X.H., G.Q.W, H.Z., S.L.H., G.J.C., Y.M.C., W.J.L., Y.L.L. supervised all aspects of the study. Y.J.C. and C.L.L. analyzed the data and prepared the figures; Y.J.C. and C.L.L. contributed to initial data interpretation and wrote the manuscript; J.P.B. and Y.J.C. reviewed and edited the article.

## Supporting information



Supporting Information

Supplemental Figures

Supplemental Tables

Supplemental Table 1

Supplemental Table 2

Supplemental Table 3

Supplemental Table 4

Supplemental Table 5

## Data Availability

The data that support the findings of this study are available from the corresponding author upon reasonable request.
