# Supplementary material for: RNA‐Binding Protein Hnrnpa1 Triggers Daughter Cardiomyocyte Formation by Promoting Cardiomyocyte Dedifferentiation and Cell Cycle Activity in a Post‐Transcriptional Manner
Source: Adv Sci (Weinh). 2024 Nov 19;12(2):2402371. doi: 10.1002/advs.202402371 (PMC11727271; doi:10.1002/advs.202402371)
Supplement: Supplementary file 1 — Supporting Information [file ADVS-12-2402371-s005.docx]

**RNA-binding protein Hnrnpa1 triggers daughter cardiomyocyte formation by promoting cardiomyocyte dedifferentiation and cell cycle activity in a post-transcriptional manner**

Chuling Li ^1,2,3#^, Yijin Chen^1,2,3#^**^†^**, Qiqi Chen^1,2,3#^, Haoxiang Huang^1,2,3^，Michael Hesse^4^, Yilin Zhou^1,2,3^, Ming Jin^1,2,3^, Yu Liu^1,2,3^, Yifei Ruan^1,2,3^, Xiang He^1,2,3^, Guoquan Wei^1,2,3^, Hao Zheng^1,2,3^, Senlin Huang^1,2,3^, Guojun Chen^1,2,3^, Wangjun Liao^5^, Yulin Liao^1,2,3^, Yanmei Chen^1,2,3^**^†^**, Jianping Bin^1,2,3^**^†^**

^1^Department of Cardiology, State Key Laboratory of Organ Failure Research, Nanfang Hospital, Southern Medical University, 510515, Guangzhou, China;

^2^Guangdong Provincial Key Laboratory of Cardiac Function and Microcirculation, 510515 Guangzhou, China;

^3^Bioland Laboratory (Guangzhou Regenerative Medicine and Health Guangdong Laboratory), 510005, Guangzhou, China;

^4^Institute of Physiology I, Life and Brain Center, Medical Faculty, University of Bonn, Bonn, Germany.

^5^Department of Oncology, Nanfang Hospital, Southern Medical University, Guangzhou, 510515, China;

^#^ Chuling Li, Yijin Chen and Qiqi Chen contributed equally.

**^†^**Jianping Bin, Yijin Chen and Yamei Chen corresponded to this work. Email: [jianpingbin@hotmail.com](mailto:jianpingbin@hotmail.com) or [jianpingbin@126.com](mailto:jianpingbin@126.com) (Jianping, Bin); [yijinchen1@126.com](mailto:yijinchen1@126.com) (Yijin, Chen); yanmei0812@126.com (Yanmei, Chen).

**Supplemental Methods and Materials**

**SnRNA-seq Data Analysis**

***Data acquisition and code availability***

The publicly available processed UMI count data of each gene and nucleus (GSE130699 and GSE128628) from mouse hearts at various developmental stages, as well as the integrated processed scRNA-seq data of fetal and adult human hearts (GSE216019), were obtained from the NCBI GEO database.

***Quality control and normalization***

The analysis of the single nucleus gene expression matrix was conducted using the R package Seurat (4.0.2). Initially, we filtered out low-quality nuclei based on criteria in which the number of UMI counts (nCount_RNA) was less than 40,000, the number of expressed genes (nFeature_RNA) exceeded 200, and the fraction of all the counts belonging to mitochondrial genes (percent.mt) was less than 20%. The remaining nucleus were then subjected to normalization using the "LogNormalize" method in the NormalizeData function. Top 2000 highly variable genes were identified using FindVariableFeatures function with the "vst" method. The ScaleData function was subsequently used to perform standardized calculations on the normalized data.

***Unsupervised clustering analysis and sub-clustering analysis***

Highly variable genes were used to perform Principal Component Analysis (PCA). To mitigate the batch effect and integrate the scaled data, we utilized the harmony R package ([https://github.com/immunogenomics/harmony](https://github.com/immunogenomics/harmony)). By considering the top 20 principal components, unsupervised cell clustering was achieved using the Seurat FindNeighbors and FindClusters function. For data visualization, we employed Uniform Manifold Approximation and Projection (UMAP) to project cells onto a 2D space based on aligned canonical correlation analysis. The Spearman’s rank coefficient was used to evaluate the correlation among clusters. To identify cluster-specific genes, we utilized the FindAllMarkers function with a Wilcoxon rank sum test in Seurat. Manual annotation of cell types was performed based on the correlation among clusters and highly specific marker genes associated with each cluster. To focus our analysis on cardiomyocytes, we re-clustered the single-nucleus transcriptome data from cardiomyocytes.

The integration of 15 distinct CM subtypes was performed by referring the previous study^[1](#_ENREF_1)^ which integrated cell subtypes on the basis of specific marker genes and the previous study^[2](#_ENREF_2)^ which integrated cell subtypes on the basis of gene expression correlation analysis. In brief, the analytical process was summarized as follows:

Firstly, the preliminary evaluation and comparison of the selected specific marker gene expression among 15 CM subtypes were performed. Through this step, which CM subtypes might be suitable for integration was initially determined. The source references of selected specific marker genes and their related descriptions were concluded in **Table S1**.

Secondly, to integrate CM subtypes more accurately and objectively, the Spearman’s rank correlation coefficient was employed to evaluate the relationships among CM subtypes. In detail, the average expression for each subtype was first determined by using the AverageExpression function and then selected the top 2000 highly variable genes on the basis of the standard deviation. Subsequently, the ‘cor’ function with the ‘spearman’ method was used to calculate the Spearman’s correlation matrix of CM subtypes. Through this step, 15 distinct CM subtypes were integrated into 6 CM clusters.

***Single-cell trajectory analysis***

To infer the developmental trajectory of cardiomyocytes during heart development, cells were ordered in pseudotime using Monocle (v2.28.0) package in R. The cardiomyocyte gene expression matrix and annotation obtained from the Seurat object were extracted and utilized as input. Normalization was carried out using the estimateSizeFactors and estimateDispersions function, and reduce Dimension command was used for dimensionality reduction with the method of “DDRTree”. The resulting trajectories were visualized using the plot_cell_trajectory command with default parameters. After constructing the cell trajectories, we identified marker genes between different states by using the FindAllMarkers function with a Wilcoxon rank sum test in Seurat. Compared to other states, genes with adjust p-value < 0.001 and average log2 fold change> 0.25 in the target states were designed as marker genes. The top 30 high expression marker genes of each state were visualized using plot_genes_branched_heatmap function. Genes with a changing expression at different time points were screened and clustered using Mfuzz package.

***RNA velocity analysis***

RNA velocity analysis was performed using the Python package scVelo^[3](#_ENREF_3)^ with the dynamical model.

***Functional enrichment analysis***

Enriched GO and KEGG pathway enrichment analysis were conducted using the clusterProfiler package.

***Protein-protein interaction (PPI) analysis***

The interaction network of proteins was analyzed using the STRING: functional protein association networks (<https://string-db.org>). Centrality measures, including degree centrality, closeness centrality, betweenness centrality, eigenvector centrality and PageRank centrality, were evaluated by igragh package. The resulting network was visualized using Cytoscape (v3.9).

**RNA extraction, reverse transcription and quantitative RT-PCR**

Total RNA was extracted using the Total RNA Kit II (Omega) and then reverse-transcribed into single-stranded cDNA using the PrimeScript™ RT reagent Kit (TaKaRa Bio, China) according to the manufacturer’s instructions. Quantitative real-time PCR was conducted using the SYBR Green PCR Master Mix (TaKaRa Bio, China) and analyzed on a LightCycler480 (Roche). Gapdh was used as an internal control, and the mRNA expression levels in each sample were normalized to the internal control using the ΔΔCt method. The primers used for qRT-PCR are listed in **Table S2**.

**Western blot**

Cells or tissues were homogenized in RIPA buffer supplemented with a 1:100 protease (Sigma, P8340) and 1:100 phosphatase inhibitor cocktails (Sigma, P5726 and P0044). Protein concentrations were determined using the BCA protein assay (Beyotime, China). The samples were mixed with loading buffer and heated at 100 °C for 15 min. Approximately 20-40 μg of protein per lane was loaded on the SDS-page gel. The protein samples were subsequently separated by 8-12% SDS-PAGE and transferred onto polyvinylidene difluoride (PVDF) membranes (Millipore). The membranes were blocked with 5% BSA in TBST at 37°C for 1h and then probed with the primary antibodies overnight at 4°C. Following three washes, the membranes were incubated with secondary antibodies for 1 hour at room temperature. The results were visualized using an Odyssey detection system (LI-COR Biosciences, Lincoln, NE, USA) and the signal intensity of the bands was quantified using ImageJ software. The antibodies used for western blotting are listed in **Table S3**.

**Cardiomyocytes isolation and treatment**

***Isolation of P1 mouse ventricular CMs***

Ventricular CMs of 1-day-old mice were isolated as previously described^[4](#_ENREF_4)^. Briefly, ventricular tissues were harvested and shredded. After washing out red blood cells, ventricular tissues were digested with 0.25% trypsin (Invitrogen) at 4°C for 14 hours. The pre-digested ventricular tissues were then transferred to a PBS buffer containing collagenase II (1 mg/ml; Gibco) and bovine serum albumin (5 mg/ml; Sigma), and incubated under constant stirring for 20 mins at 37°C. The supernatant was centrifuged at 1,000 rpm for 5 mins, and the resulting pellet was resuspended in DMEM/F12 medium supplemented with 10% FBS_._ The collected cells were seeded onto 100-mm plastic dishes at 37°C with 5% CO_2_ for 2 hours_._ Nonadherent CMs were then collected and plated at certain densities on culture plates. CMs were transfected with siRNA and harvested 48 hours later.

***Isolation of P7 mouse ventricular CMs***

Ventricular CMs of 7-day-old mice were isolated following a previously described protocol with some modifications^[5](#_ENREF_5)^. Briefly, ventricular tissues were dissected and washed with HBSS without Ca^2+^ and Mg^2+^ (MacGene). The ventricular tissues were then cut into 1 mm^3^ pieces and dissociated in a HEPES buffer containing pancreatin (0.25 mg/ml; Sigma), collagenase II (0.125 mg/ml; Worthington Biochemical) and DNase II (10 µg/ml; Sigma) under constant stirring for 5 mins at 37°C. Digestions at 5-min steps were repeated 8 to 10 times at room temperature until tissues became flocculent. The supernatant was centrifuged at 1,000 rpm for 5 mins, and the resulting pellet was resuspended in DMEM/F12 medium supplemented with 10% FBS_._ The collected cells were seeded onto 100-mm plastic dishes and incubated at 37°C with 5% CO_2_ for 2 hours_._ The Nonadherent CMs were then collected and incubated on culture plates at certain densities. CMs were then transducted with adenovirus-Hnrnpa1 (Adv-Hnrnpa1), adenovirus-Mettl3-L (Adv-Mettl3-L), adenovirus-Mettl3-S (Adv-Mettl3-S), or adenovirus-negative control (Adv-NC) at a 20 multiplicity of infection (MOI=20), and harvested 48 hours later.

***Isolation of adult mouse CMs***

Adult CMs were isolated from adult mouse heart treated with AAV9-cTnT-Hnrnpa1 or AAV9-cTnT-NC at 14 days after transduction. Briefly, mouse was anaesthetized using isoflurane (4% induction, 2% maintenance), and the heart was removed via thoracotomy. After isolating the aorta, mouse heart was connected to the Langendorff perfusion system. Mouse heart was then perfused with calcium-free perfusion buffer (in mM: NaCl 113; KCl 4.7; KH_2_PO_4_ 0.6; Na_2_HPO_4_ 0.6; MgSO_4_ 1.2; Na-HEPES 10; NaHCO_3_ 12; KHCO_3_ 10; phenol red 0.032; taurine 30; BDM 10; glucose 5.5) until all residual blood was completely pumped out. Subsequently, the heart was digested with 50 ml of perfusion buffer containing 15,000 U of type II collagenase (Roche) and 50 μM CaCl_2_ at 37°C until the heart slightly appeared slightly pale and flaccid. The digested heart was gently divided into small pieces with a forceps and then triturated with a Pasteur pipette to dissociate individual CMs. The resulting cell suspension was collected and centrifuged at 1,000 rpm for 5 min to obtain CM pellet. The pelleted cells were then incubated on laminin-coated culture plates at certain densities for 2 h, after which the medium was changed from plating medium to culture medium (DMEM/F12 medium supplemented with 10% FBS) to filter non-CMs.

***Isolation and culture of neonatal mouse cardiac ECs***

Neonatal mouse cardiac endothelial cells were isolated and cultured as our previous study described^[6](#_ENREF_6)^. Briefly, shortly after being dissected, neonatal mouse hearts were cleaned with sterile PBS, cut into pieces, and digested in 0.25% trypsin (Invitrogen) for 5 minutes at 37°C. The heart tissue was further digested in 0.1% type II collagenase (Worthington Biochemical) at 37°C after centrifugation. After filtration and centrifugation, the separated cells were washed, resuspended, and then incubated with magnetic beads conjugated with an anti-CD31 antibody. Finally, the beads with ECs were washed several times and were ready for using in subsequent applications. Fluorescence with anti-CD31 was used for the identification, purity and population of ECs. ECs were then cultured in DMEM/F12 medium supplemented with 10% FBS and incubated at 37°C with 5% CO_2_.

***Isolation and culture of neonatal mouse cardiac fibroblasts***

Neonatal mouse cardiac fibroblasts were isolated and cultured as a previous study described with slight alterations^[7](#_ENREF_7)^_._ Briefly, after being harvested, neonatal mouse hearts were cut into 1 mm^3^ pieces and dissociated in PBS buffer containing 0.25% trypsin (Invitrogen) and 0.1% type II collagenase (Worthington Biochemical) under constant shaking for 7 min at 250 rpm and 37°C. Digestions at 7-min steps were repeated 10 times at room temperature until the tissues became flocculent. The supernatant was collected in horse serum and incubated on poly-L-lysine-coated culture plates for 1 hour. The supernatant was then discarded, followed by washing with medium to remove other cell types. Finally, the attached cardiac fibroblasts were cultured in DMEM/F12 medium supplemented with 10% FBS and incubated at 37°C with 5% CO_2._

***Human iPSC-CM culture***

CMs derived from human iPSCs were purchased from Cellapy (Beijing, China), and these hiPSC-CMs were cultured as our previous study described^[8](#_ENREF_8)^. Briefly, hiPSC-CMs were allowed to adhere for 48 h before maintenance medium exchange and the culture medium was replaced every other day. Then, the cells were transduced with Adv-Hnrnpa1 or Adv-NC at a 20 multiplicity of infection (MOI=20) and harvested 48 hours later.

***siRNA transfection***

For siRNA experiments, P1 CMs were transfected with siRNA-Hnrnpa1 (50 nM), siRNA-total Mettl3 or siRNA-NC (50nM) using Lipofectamine RNAiMAX (Invitrogen) following the manufacturer's instructions. The target sequences of siRNA-Hnrnpa1-1, siRNA-Hnrnpa1-2 and siRNA-Hnrnpa1-3 were: 5′-CCTGGTTACTCTGGAGGAA-3′, 5′-TGGACAGGGTTATGGAAAC-3′ and 5′-CAGCTATAACAA CGGAGGA-3′, respectively. The target sequence of siRNA-total Mettl3 was: 5′- GAGTTGATTGAGGTAAAGCGAGG-3′.

***Recombinant adenovirus construction***

The mouse Hnrnpa1 sequences (NM_001039129.5) were cloned into the pAdeno vector (pAdeno-MCMV-MCS-3FLAG-IRES-EGFP) backbone, and the adenovirus packaging was conducted by Kidan (Guangzhou, China). The sgRNA (Hnrnpa1) sequences were cloned into the pAdeno vector (pAdeno-U6-sgRNA-CRE-T2A-EGFP) backbone, and the adenovirus packaging was produced by Genechem (Shanghai, China). The target sequence of sgHnrnpa1 was 5′-GTTTGTCACATATG CCACT-3′.

***Minigene Construction***

For the construction of the pcDNA3.1-Mettl3-minigene vectors, the Mettl3-minigene genomic sequences spanning exons 3 to 5 were synthesized and then cloned and inserted into the pcDNA3.1 (+) vector. The Mettl3 minigene fragment works by expressing the Mettl3 exon 3-intron 3-exon 4-intron 4-exon 5 sequence in isolated CMs, which could help to further determine whether Hnrnpa1 plays an important role in exon 4 skipping of Mettl3.

***Nuclei isolation and Ki67 detection by flow cytometry***

Nuclei were isolated using a modified protocol from the Nuclei Isolation Kit (Sigma, NUC201). Briefly, hearts from adult mice were digested using the Langendorff perfusion technique. Following digestion, the ventricles were removed, diced, and quickly frozen in liquid nitrogen. 10 ml of lysis buffer plus 1 mM DTT (Sigma, D0632) were added to the frozen tissue. The tissue was then homogenized using an Omni TH115 homogenizer at low speed for 10 s, followed by 10 strokes with a loose-fitting Dounce homogenizer and 10 strokes with a tight-fitting Dounce homogenizer. The homogenate tissue was then strained through a 70-μm mesh and two 40-μm meshes. Next, 20 ml of 2M sucrose buffer (with 1 mM DTT) was added to each sample and mixed. 10 ml of 2M sucrose buffer was added to the bottom of the ultracentrifuge tube and the sample was gently poured on top. After centrifugation at 13,000xg for 1 hour at 4 °C using a swing-out rotor, the nuclear pellets were precipitated at the bottom. After removing the supernatant, nuclear pellets were resuspended in 5% BSA in PBS and then fixed/permeabilized in Fix/Perm (BD Biosciences, 554722) for 20 min at 4°C. After washing with 5% BSA in PBS, resuspended fixed/permeabilized nuclei were stained with preconjugated PCM1-CoraLite 555 (Proteintech, CL555-19856 1:250) and Ki67-Alexa Fluor 488 (Abcam, ab281847 1:5000) for 30 min at room temperature to detect the cell cycle activity of the cardiomyocyte nuclei. Flow cytometry was conducted using FACScan (BD Biosciences), and the data were analyzed with FlowJo software (version 10.8.1).

**Animal Models and Treatments**

**Myocardial Cas9 knock-in transgenic mouse model**

The Cre-dependent Cas9 knock-in mouse model was obtained from GemPharmatech (Jiangsu, China). The targeting vector used in this model was designed to incorporate a ubiquitously expressed CAG promoter, a loxP-flanked PGK-Neo-polyA sequence followed by a Cas9 protein inserted into intron 1 of the Rosa26 locus. After confirming the correct insertion, the construct was linearized and electroporated into JM8A3 embryonic stem cells. Subsequently, the JM8A3 embryonic stem cells were implanted in C57BL/6J blastocysts to generate chimeric mice and the high-percentage chimeric male were then crossed with female C57BL/6J mice to produce heterozygous Cre-dependent Cas9 mice (Rosa26-LSL-Cas9-tdTomato/+). Heterozygotes were further intercrossed to obtain homozygotes. The homozygous Rosa26-LSL-Cas9-tdTomato mice were subsequently crossed with α-MHC-Cre transgenic mice to generate myocardial Rosa26-Cas9-tdTomato mice.

**Neonatal mouse MI model**

MI surgery was performed in P1 mice as previously described^[9](#_ENREF_9)^. Briefly, P1 mice with myocardial Cas9-tdTomato expression were anaesthetized on an ice bed for 4-5 mins. Lateral thoracotomy at the fourth intercostal space was conducted by blunt dissection of the intercostal muscles. Subsequently, the left anterior descending coronary artery (LAD) was ligated using 8-0 silk sutures. After MI surgery, Adv vectors containing sgRNA were immediately injected into the myocardium at 4 sites with a dose of 1 × 10^10^ viral genome particles per animal using an insulin syringe. The neonatal pups were then transferred from the ice bed, and the thoracic incision and skin wounds were properly closed using 8-0 nonabsorbable silk sutures. Finally, the neonatal pups were placed under a heat lamp for a few minutes until they recovered, after which they were returned to their mother. Sham-operated mice underwent similar surgical procedures without occlusion of the coronary artery.

**Adult mouse MI model**

After anesthetized with isoflurane (4% induction, 2% maintenance), adult mouse MI surgery was induced by ligation of the LAD as previously described^[9](#_ENREF_9)^. After ligation, AAV9-cTnT-NC or AAV9-cTnT-Hnrnpa1 viral genome particles were injected into the myocardium of MI mouse hearts at multiple sites (5-6 sites with a dose of 1×10^11^ viral genome particles in a total volume of 30μL per animal using an insulin syringe), which started from the risky border zone and then gradually expanded to the safe remote zone. Finally, the dissected intercostal space and chest skin incision were closed using 5-0 silk sutures. Sham-operated mice underwent similar surgical procedures without occlusion of the coronary artery.

**Immunostaining**

Treated cardiomyocytes and heart sections (4 μm) were washed with PBS three times and fixed with 4% paraformaldehyde at room temperature for 30 mins. After washed with PBS three times, samples were permeabilized with 0.5% Triton X-100 in PBS at room temperature for 10 mins and then blocked with 1% BSA in PBS at room temperature for 1 hour. The samples were then incubated with the primary antibodies diluted in 1% BSA blocking solution at 4°C for 24 hours. Following three PBS washes, the samples were incubated with the secondary antibodies at room temperature for 1 hour, followed by incubation with the cell nuclei indicator DAPI at room temperature for 10 mins. For EdU labeling experiments in vitro, the EdU Apollo 567 in Vitro Kit (Ribobio, China) was used. Following the manufacturer’s instructions, cell samples were treated with EdU at a concentration of 50 µM for 12 hours prior to fixation. After fixation and penetration, cell samples were labeled with Apollo mixture reagent to evaluate DNA synthesis and then performed similar steps refer to above experiments. For measuring the cross-sectional area of cardiomyocytes, heart sections were stained with wheat germ agglutinin (WGA; AlexaFluor 647 conjugate; Invitrogen) and DAPI. Cardiomyocytes with a centrally located nucleus and a circularity index between 1.0 and 0.895 (radius ratio of 1:1 to 1:1.4) was considered for measurement. The primary and secondary antibodies are listed in **Table S4**.

**Echocardiography**

Cardiac ultrasound echocardiography of approximately 14-day-old, 28-day-old, and adult mice were performed by using a same Vevo 2100 imaging system (VisualSonics, Toronto, Canada) equipped with a 30-MHz MS-400 transducer. In brief, mice were slightly anesthetized by 0.5–1.0% isoflurane. Two-dimensional guided M-mode tracings in the parasternal short axis view were used to measure the left ventricular internal diameter at end-diastole (LVEDd) and end-systole (LVESd), which were used to calculate the left ventricular fractional shortening (LVFS) and the left ventricular ejection fraction (LVEF). All measurements were performed from more than three beats and averaged for analysis.

**Triphenyltetrazolium chloride (TTC) staining**

After isolation and briefly freezing, the adult mouse heart was sectioned into serial 3-mm sections. These heart sections were then incubated in a 2% TTC (Solarbio, China) staining solution at room temperature for 30 mins, followed by washed with PBS three times and finally photographed. The percentage of the infarcted area in the left ventricular was calculated by using the Image-Pro Plus 6.0.

**Histology**

Heart tissues were fixed in 4% paraformaldehyde at room temperature for 24 hours and then subjected to a series of ethanol dehydration and paraffin embedding. Subsequently, heart tissues were divided into serial 8-µm sections. Masson’s trichrome staining was performed to evaluate the degree of cardiac fibrosis in each MI model according to manufacturer’s instructions (MST-8004, MXB Biotechnologies). The percentage of the fibrotic area in the left ventricle was calculated using the Image-Pro Plus 6.0.

**Nanopore RNA Sequencing Analysis**

***Sample selection***

Compared with the isolation of adult CMs that required heart-by-heart processing due to the Langendorff perfusion system, the isolation of P7 CMs could process multiple hearts at the same time, which would make it easier to obtain enough CMs to meet the cell number requirement of nanopore RNA-seq (approximately 5×10^7^ cells) ^[10](#_ENREF_10)^. Therefore, P7 CMs were used for nanopore RNA-seq in this study.

***Transcriptional analysis***

The resulting FAST5 files generated by the nanopore sequencing instrument were converted into FASTQ format via the GUPPY tool from the MinKNOW software package. The mean Q score of each sample FASTQ files in our study was 11 **(Table S5)**, which exceeded the threshold of “7”^[11](#_ENREF_11)^, indicating that all sample FASTQ files qualified for subsequent analysis. Low-quality reads were filtered out using nanofilt software and clean data were then mapped to the mouse genome (mm10) using mimimap2 software. Consensus isoforms were obtained after polishing within each cluster by pinfish and mapped reads were further collapsed using the cDNA_Cupcake package. Reads with a match quality＞5 were selected for quantification and the expression levels were estimated as reads per gene/transcript per 10,000 mapped reads. The DESeq2 R package was used to perform the differential expression analysis of two groups. Genes with a p value<0.05 and |log2 fold change|>0.5 were considered differentially expressed. GO, KEGG pathway and GSEA analysis were performed using the clusterProfiler package.

***Gene Set Variation Analysis***

Gene set variation analysis (GSVA), a particular type of gene set enrichment method for estimating the variation of gene set enrichment in a dataset of expression samples, was conducted using the "GSVA" R package^[12](#_ENREF_12)^. The combined z-score method was used to compute an enrichment score for each gene set and individual sample. The gene sets used in this study were obtained from the MSigDB database for GSVA. Pearson's correlation analysis was performed to examine the relationship between the regulation of RNA splicing and other biological pathways**.**

***Alternative splicing analysis***

Alternative splicing (AS) analysis of Nanopore RNA-seq was performed using PSI-Sigma (https://github.com/wososa/PSI-Sigma). The Percent Spliced In (PSI) value is defined as the ratio of inclusion/exclusion normalized read counts as a percentage of the total (both inclusion and exclusion) normalized read counts for that event and was calculated for five types of AS events. To generate a reliable set of AS events, we implemented a series of stringent filters (percentage of samples with PSI ≥75, average PSI ≥ 0.05). AS events with a Δ|PSI|> 0.05 and a p value<0.05 were considered to indicate statistical significance. GO and KEGG pathway enrichment analysis of the significant AS events were implemented using the clusterProfiler package.

**RNA immunoprecipitation assay**

The RNA immunoprecipitation (RIP) assay was performed using the Magna RIP RNA-Binding Protein Immunoprecipitation Kit (Millipore, USA) according to manufacturer’s instructions. P7 CMs were transfected with Adv-Hnrnpa1 and subjected to RIP experiments using the Hnrnpa1 antibody (Santa, sc-32301). For the Mettl3 RIP assay, the primers sequences were AGGAACCCAGAAGCGGC ACT (forward) and GGCAAGACGGATGGAAACAG (reverse). For the Gapdh RIP assay, the primer sequences were ACTCACGGCAAATTCAAC (forward) and CCACGACATACTCA GCAC (reverse).

**Calculation of Delta PSI**

The calculation formula of the PSI is: PSI=splice_in/(splice_in+splice_out), and the calculation formula of Delta PSI is: Delta PSI=PSI(Adv-Hnrnpa1)-PSI(Adv-NC). When Delta PSI＞0, there are more inclusive transcripts in the Adv-Hnrnpa1 group than in the Adv-NC group. When Delta PSI＜0, there are more inclusive transcripts in the Adv-NC group than in the Adv-Hnrnpa1 group. PSI, percent spliced in.

**Semi-quantitative RT-PCR**

Total RNA was extracted using the Total RNA Kit II (Omega) and then reverse-transcribed into cDNA using the cDNA Synthesis Kit (Yeasen, China). Semiquantitative RT-PCR was performed using the 2×Hieff® HotStart PCR Genotyping Master Mix (Yeasen, China). The inclusion/ exclusion forms of exon4 of Mettl3 were detected by RT-PCR, and these two isoforms were depicted on 2% agarose gels. Gapdh was used as an internal control. The primer sequences for the semi-quantitative RT-PCR are listed in **Table S6**.

**Protein-RNA interaction prediction**

Predicted RNA interactions with Mettl3-L were obtained from the RNAct website (http://rnact.crg.eu/), a database of protein-RNA interactions calculated on the basis of the catRAPID website (<http://service.tartaglialab.com/page/catrapid_group>). This algorithm estimates the binding propensity of protein-RNA pairs by combining secondary structure, hydrogen bonding, and van der Waals contributions^[13](#_ENREF_13)^. Given that the RNAct website does not include the Mettl3-S protein, we predicted potential RNA interactions of the METTL3-S protein via the catRAPID website. The output is filtered on the basis of the Z-score, which represents the interaction propensity normalized by the mean and standard deviation calculated from the reference RBP set. For our analysis, only predicted interactions with a Z-score greater than 1 were considered.

**Prediction of protein structure and interaction**

The protein sequence of Mettl3-S was analyzed via the open reading frame (ORF) Finder and BLAST tools available at NCBI (<http://www.ncbi.nlm.nih.gov/>), which comprises 258 amino acids. The protein sequence of Mettl3-L, which comprises 580 amino acids, was obtained from the Ensembl Database. The protein structures of Mettl3-L and Mettl3-S and their interaction prediction analysis were conducted via ColabFold (version 1.5.5) with default parameters. As predicted by ColabFold^[14](#_ENREF_14)^, the interface pTM (ipTM)+predicted template modeling (pTM) scores for the complexes of Mettl3-L and Mettl3-S were 0.782+0.549=1.331 and exceeded the threshold of 0.75, which indicated that the interaction model of Mettl3-L and Mettl3-S can be adopted with reasonable confidence. The structures and docking interactions of the Mettl3-L and Mettl3-S proteins were visualized via PyMOL (version 2.5.4).

**Mass spectrometry**

After transfected with Adv-Flag-GFP or Adv-Flag-Mettl3-S for 48h, isolated P7 CMs were washed with PBS three times and lysed with immunoprecipitation lysis buffer containing 100 μM phenylmethylsulfonyl fluoride (PMSF, Beyotime, China). The CM cell lysates were then subjected to immunoprecipitation with 50 μl protein A-agarose and anti-Flag antibody. The candidate proteins interacting with Mettl3-S were identified by mass spectroscopy. The digested peptides were analyzed using Orbitrap Fusion™ Lumos™ Tribrid™ mass spectrometer (Thermo Fisher Scientific, Waltham, MA, USA). The mass spectrometry data files were processed using MaxQuant software (v2.6.4.0) for label-free quantification (LFQ) and searched against the UniProtKB mouse protein sequence database (UP000000589). The default of 2 miscleavages was allowed for global proteins were and the protein FDR (false discovery rate) levels were set at 1%. To visualize the representative MS/MS spectra of tryptic peptides, the mass spectrometry data was firstly converted to “mgf” format using the MSConvert part in ProteoWizard and then visualized using the pLable(v2.4) software.

**Co-immunoprecipitation assay**

After P7 CMs co-transfected with Adv-Flag-Mettl3-S and Adv-HA-Mettl3-L for 48h, cell samples were washed twice with cold PBS and lysed in 1.5 ml of cold lysis buffer (50 mM Tris-HCl, pH 7.6, 150 mM NaCl, 0.1% Triton X-100, 1 mM sodium orthovanadate, 1 mM sodium fluoride, 1 mM sodium pyrophosphate, 10 mg ml-1 aprotinin, 10 mg ml-1 leupeptin, 2 mM phenylmethylsulfonyl fluoride and 1 mM EDTA) on ice. Subsequently, the lysates were incubated with an anti-Flag antibody (MBL, Cat#PM020) for 1 h followed by protein A/G-agarose beads (Millipore, USA) at 4°C for 12 h. The immunoprecipitates were pelleted, washed and subjected to immunoblotting using an anti-Flag antibody (MBL, Cat#M183-3L) and anti-HA antibody (Millipore, Cat#H3663).

**RNA m6A quantification**

RNA m6A quantification was performed following a previously described protocol^[15](#_ENREF_15)^. The isolated total RNA was quantified by a NanoDrop spectrophotometer after treated with deoxyribonuclease I (AMPD1-1KT, Sigma). An m6A RNA methylation quantification kit (ab185912; Abcam) was then used to detect the m6A content of the total RNA. In brief, 200 ng of RNA was coated onto the wells of an assay plate, followed by adding a capture antibody solution (1:1000) and a detection antibody solution (1:2000) according to the manufacturer’s protocol. The m6A levels were quantified colorimetrically by measuring the absorbance at 450 nm using a microplate reader.

**m6A-RIP-qPCR**

The MeRIP-qPCR was performed as previously described^[16](#_ENREF_16)^. Briefly, 100 μg DNAase-treated RNA samples were incubated with m6A-antibody-bound protein G beads which were pre-blocked with 1% BSA in an immunoprecipitation buffer (150 mM NaCl, 10 mM Tris-HCl, and 0.1% NP-40 containing protease and RNAse inhibitor) under gentle rotation for 12 hours at 4°C. Beads were then washed with the immunoprecipitation buffer, and RNA was eluted twice using an m6A-free nucleotide solution. Subsequently, the elutions containing m6A-enriched transcripts were subjected to phenol-chloroform extraction for RNA purification, followed by qRT-PCR analysis. The primer sequences for the m6A-RIP-qPCR were listed in **Table S2**, and the primers used for detecting the m6A enrichment levels of different Pbx1 or E2F1 regions were listed in **Table S7-8**.

**PAR-CLIP**

The PAR-CLIP was performed as previously described^[17](#_ENREF_17)^. P7 CMs were incubated with 100 mM 4-thiopyridine (4SU) for 16 hours, and cellular DNA and protein were crosslinked with UV radiation at 365 nm. After lysis, samples were centrifuged at 12,000×g, and the supernatant containing proteins was obtained. Input samples were reserved for RNA and protein analysis, while the remaining supernatant was divided into two equal parts. Protein G beads (Invitrogen) were prepared according to manufacturer’s instructions. The two parts were incubated with either rabbit IgG or an anti-Mettl3-L antibody overnight at 4°C. Subsequently, the samples were incubated with beads for 2 hours and placed on a magnetic separator. The supernatant was discarded and the beads were washed twice with buffer A, twice with buffer B and twice with buffer C. One-quarter of the beads were mixed with 5×SDS loading buffer for protein analysis, and the remaining beads were put into a buffer containing 107 μL NT-2 buffer (50 mM Tris pH 7.4, 150 mM NaCl, 1 mM MgCl_2_, 0.05% NP-40), 15 μL 10% SDS, 2 μL RNase inhibitor and 18 μL protease K at 55°C. RNA was extracted using Trizol and subsequently analyzed to detect Pbx1 or E2F1 expression.

**Luciferase reporter assays**

cDNAs containing partial CDS sequence near stop codon and full-length 3’UTR of Pbx1 or E2F1 were cloned into pmir-GLO reporter plasmids using Tsingke (Nanjing, China). For the mutant 1 or 2 reporter plasmids, 6 or 4 adenosine (A) within the m6A motif were substituted with cytosine (C), respectively. Pre-treated CMs cells were seeded into a 24-well plate, followed by co-transfection of 0.5μg of WT, mutated Pbx1 or E2F1 reporter plasmids and 25 ng pRL-TK plasmids (renilla luciferase reporter vector) using Lipofectamine-3000 transfection reagent (Invitrogen, USA). After 24 hours, CMs were harvested to access the luciferase activity using the Dual-Glo Luciferase system (Promega, USA) with normalization to pRL-TK.

**References**

1. Bischoff P, Trinks A, Obermayer B. Single-cell rna sequencing reveals distinct tumor microenvironmental patterns in lung adenocarcinoma. ***Oncogene***. 2021;40:6748-6758

2. Ren Z, Yu P, Li D, Li Z, Liao Y, Wang Y, Zhou B, Wang L. Single-cell reconstruction of progression trajectory reveals intervention principles in pathological cardiac hypertrophy. ***Circulation***. 2020;141:1704-1719

3. Bergen V, Lange M. Generalizing rna velocity to transient cell states through dynamical modeling. ***Nature biotechnology***. 2020;38:1408-1414

4. Chen Y, Wu G, Li M, Hesse M, Ma Y, Chen W, Huang H, Liu Y, Xu W, Tang Y, Zheng H, Li C, Lin Z, Chen G, Liao W, Liao Y, Bin J, Chen Y. Ldha-mediated metabolic reprogramming promoted cardiomyocyte proliferation by alleviating ros and inducing m2 macrophage polarization. ***Redox biology***. 2022;56:102446

5. Eulalio A, Mano M, Dal Ferro M, Zentilin L, Sinagra G, Zacchigna S, Giacca M. Functional screening identifies mirnas inducing cardiac regeneration. ***Nature***. 2012;492:376-381

6. Wei G, Li C, Jia X, Xie J, Tang Z, Jin M, Chen Q, Sun Y, He S, Li X, Chen Y, Zheng H, Liao W, Liao Y, Bin J, Huang S. Extracellular vesicle-derived circwhsc1 promotes cardiomyocyte proliferation and heart repair by activating trim59/stat3/cyclin b2 pathway. ***Journal of advanced research***. 2023;53:199-218

7. Kumar S, Nagesh D, Ramasubbu V, Prabhashankar AB, Sundaresan NR. Isolation and culture of primary fibroblasts from neonatal murine hearts to study cardiac fibrosis. ***Bio-protocol***. 2023;13:e4616

8. Li M, Zheng H, Han Y, Chen Y, Li B, Chen G, Chen X, Huang S, He X, Wei G, Xu T, Feng X, Liao W, Liao Y, Chen Y, Bin J. Lncrna snhg1-driven self-reinforcing regulatory network promoted cardiac regeneration and repair after myocardial infarction. ***Theranostics***. 2021;11:9397-9414

9. Porrello ER, Mahmoud AI, Simpson E, Johnson BA, Grinsfelder D, Canseco D, Mammen PP, Rothermel BA, Olson EN, Sadek HA. Regulation of neonatal and adult mammalian heart regeneration by the mir-15 family. ***Proceedings of the National Academy of Sciences of the United States of America***. 2013;110:187-192

10. Workman RE, Tang AD. Nanopore native rna sequencing of a human poly(a) transcriptome. ***Nature Methods***. 2019;16:1297-1305

11. Harel N, Meir M, Gophna U, Stern A. Direct sequencing of rna with minion nanopore: Detecting mutations based on associations. ***Nucleic acids research***. 2019;47:e148

12. Hänzelmann S, Castelo R, Guinney J. Gsva: Gene set variation analysis for microarray and rna-seq data. ***BMC bioinformatics***. 2013;14:7

13. Armaos A, Colantoni A, Proietti G, Rupert J, Tartaglia GG. Catrapid omics v2.0: Going deeper and wider in the prediction of protein-rna interactions. ***Nucleic acids research***. 2021;49:W72-w79

14. Mirdita M, Schütze K. Colabfold: Making protein folding accessible to all. ***Nature methods***. 2022;19:679-682

15. Zhong L, He X, Song H, Sun Y, Chen G, Si X, Sun J, Chen X, Liao W, Liao Y, Bin J. Mettl3 induces aaa development and progression by modulating n6-methyladenosine-dependent primary mir34a processing. ***Molecular therapy. Nucleic acids***. 2020;21:394-411

16. Wang T, Zhou LY, Li XM, Liu F, Liang L, Chen XZ, Ju J, Ponnusamy M, Wang K, Liu CY, Yan KW, Wang K. Abro1 arrests cardiomyocyte proliferation and myocardial repair by suppressing psph. ***Molecular therapy : the journal of the American Society of Gene Therapy***. 2023;31:847-865

17. Zhang Y, Qiao X, Liu L, Han W, Liu Q, Wang Y, Xie T, Tang Y, Wang T, Meng J, Ye A, He S, Chen R, Chen C. Long noncoding rna magi2-as3 regulates the h(2)o(2) level and cell senescence via hspa8. ***Redox biology***. 2022;54:102383
