# Supplementary material for: RNA‐Binding Protein Hnrnpa1 Triggers Daughter Cardiomyocyte Formation by Promoting Cardiomyocyte Dedifferentiation and Cell Cycle Activity in a Post‐Transcriptional Manner
Source: Adv Sci (Weinh). 2024 Nov 19;12(2):2402371. doi: 10.1002/advs.202402371 (PMC11727271; doi:10.1002/advs.202402371)
Supplement: Supplementary file 2 — Supplemental Figures [file ADVS-12-2402371-s001.docx]

**RNA-binding protein Hnrnpa1 triggers daughter cardiomyocyte formation by promoting cardiomyocyte dedifferentiation and cell cycle activity in a post-transcriptional manner**

Chuling Li ^1,2,3#^, Yijin Chen^1,2,3#^**^†^**, Qiqi Chen^1,2,3#^, Haoxiang Huang^1,2,3^，Michael Hesse^4^, Yilin Zhou^1,2,3^, Ming Jin^1,2,3^, Yu Liu^1,2,3^, Yifei Ruan^1,2,3^, Xiang He^1,2,3^, Guoquan Wei^1,2,3^, Hao Zheng^1,2,3^, Senlin Huang^1,2,3^, Guojun Chen^1,2,3^, Wangjun Liao^5^, Yulin Liao^1,2,3^, Yanmei Chen^1,2,3^**^†^**, Jianping Bin^1,2,3^**^†^**

^1^Department of Cardiology, State Key Laboratory of Organ Failure Research, Nanfang Hospital, Southern Medical University, 510515, Guangzhou, China;

^2^Guangdong Provincial Key Laboratory of Cardiac Function and Microcirculation, 510515 Guangzhou, China;

^3^Bioland Laboratory (Guangzhou Regenerative Medicine and Health Guangdong Laboratory), 510005, Guangzhou, China;

^4^Institute of Physiology I, Life and Brain Center, Medical Faculty, University of Bonn, Bonn, Germany.

^5^Department of Oncology, Nanfang Hospital, Southern Medical University, Guangzhou, 510515, China;

^#^ Chuling Li, Yijin Chen and Qiqi Chen contributed equally.

**^†^**Jianping Bin, Yijin Chen and Yamei Chen corresponded to this work. Email: [jianpingbin@hotmail.com](mailto:jianpingbin@hotmail.com) or [jianpingbin@126.com](mailto:jianpingbin@126.com) (Jianping, Bin); [yijinchen1@126.com](mailto:yijinchen1@126.com) (Yijin, Chen); yanmei0812@126.com (Yanmei, Chen).

**Supplementary Figures and legends**

**
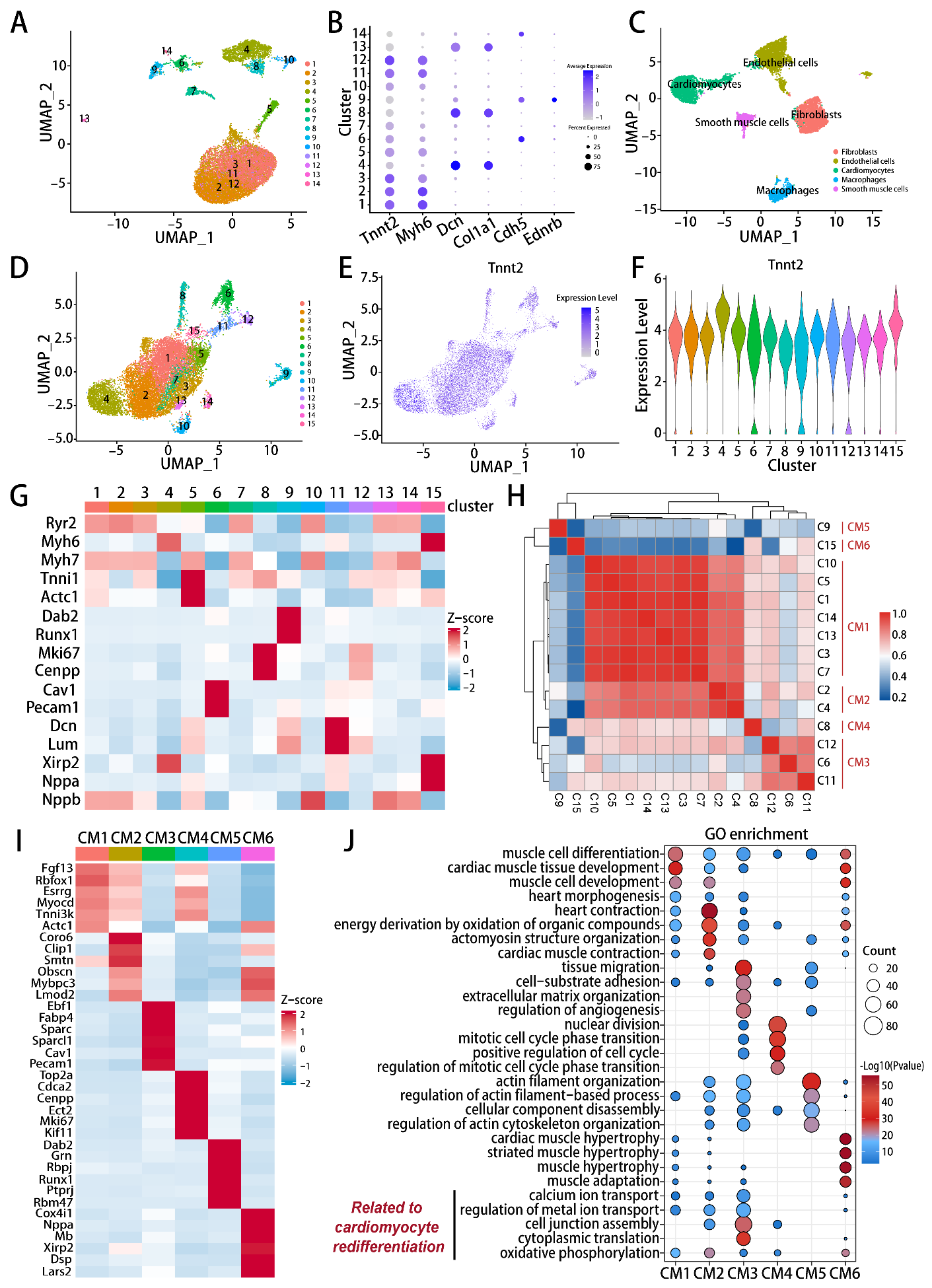
**

Supplemental Figure 1

**
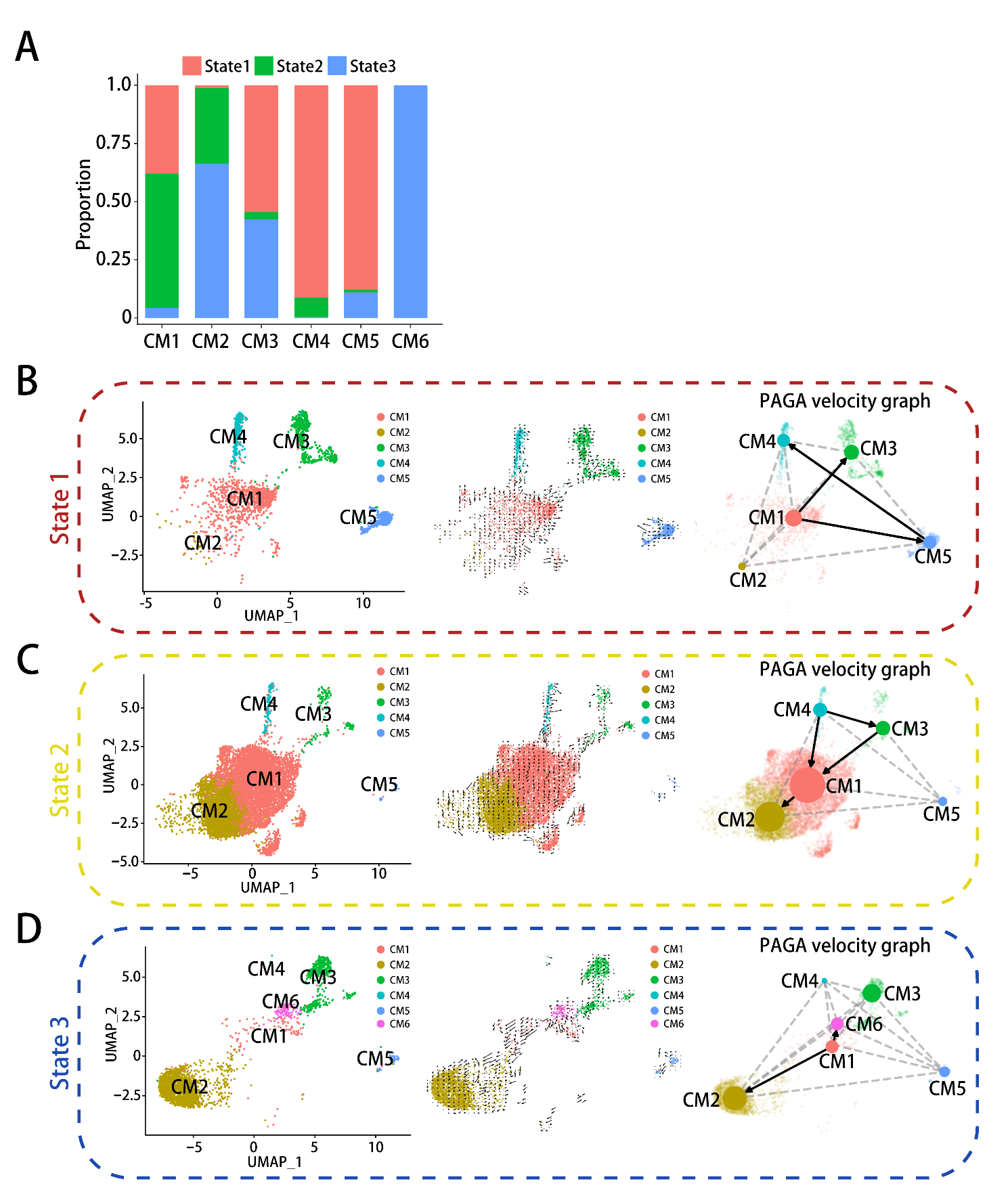
**

Supplemental Figure 2

**
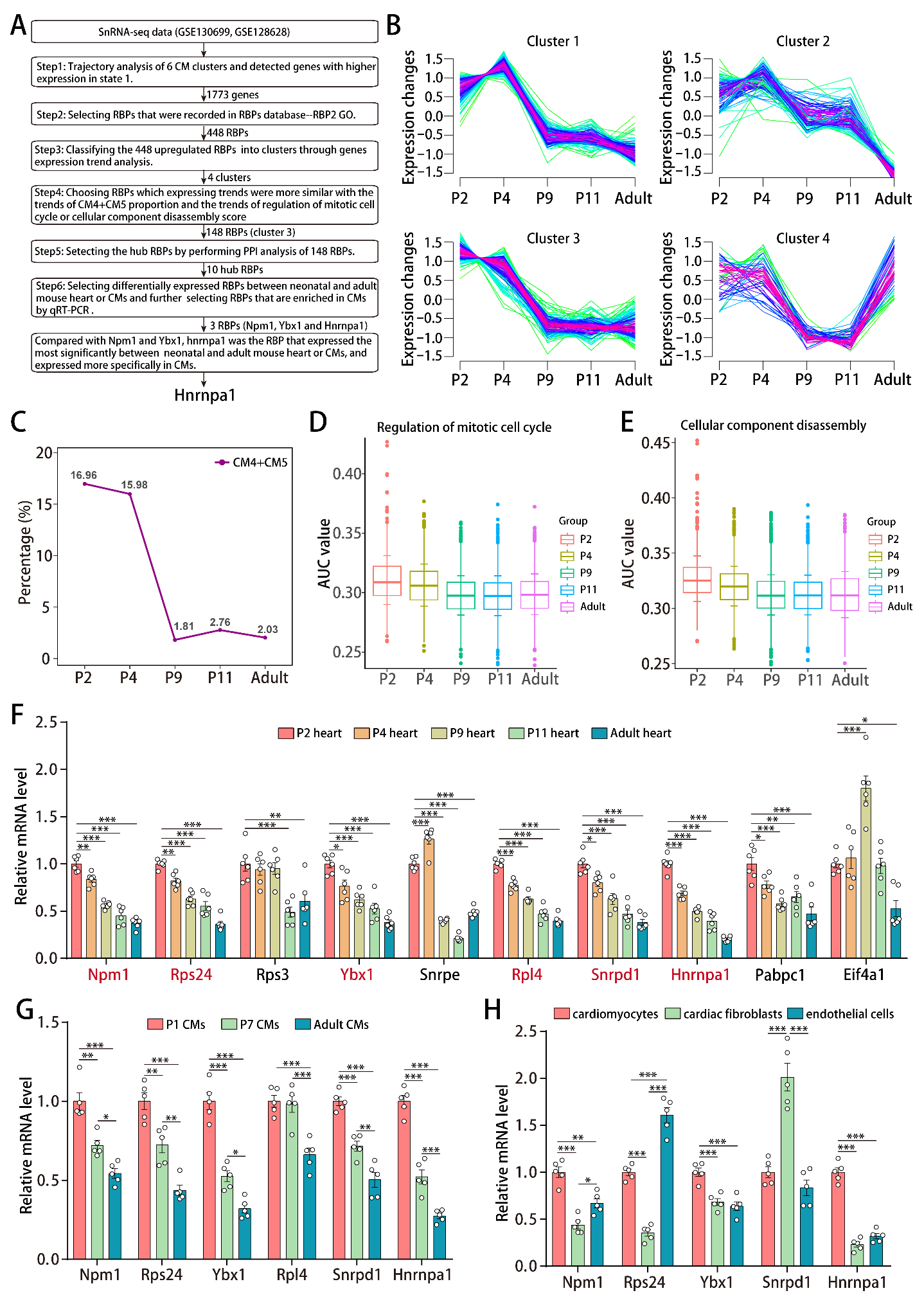
**

Supplemental Figure 3
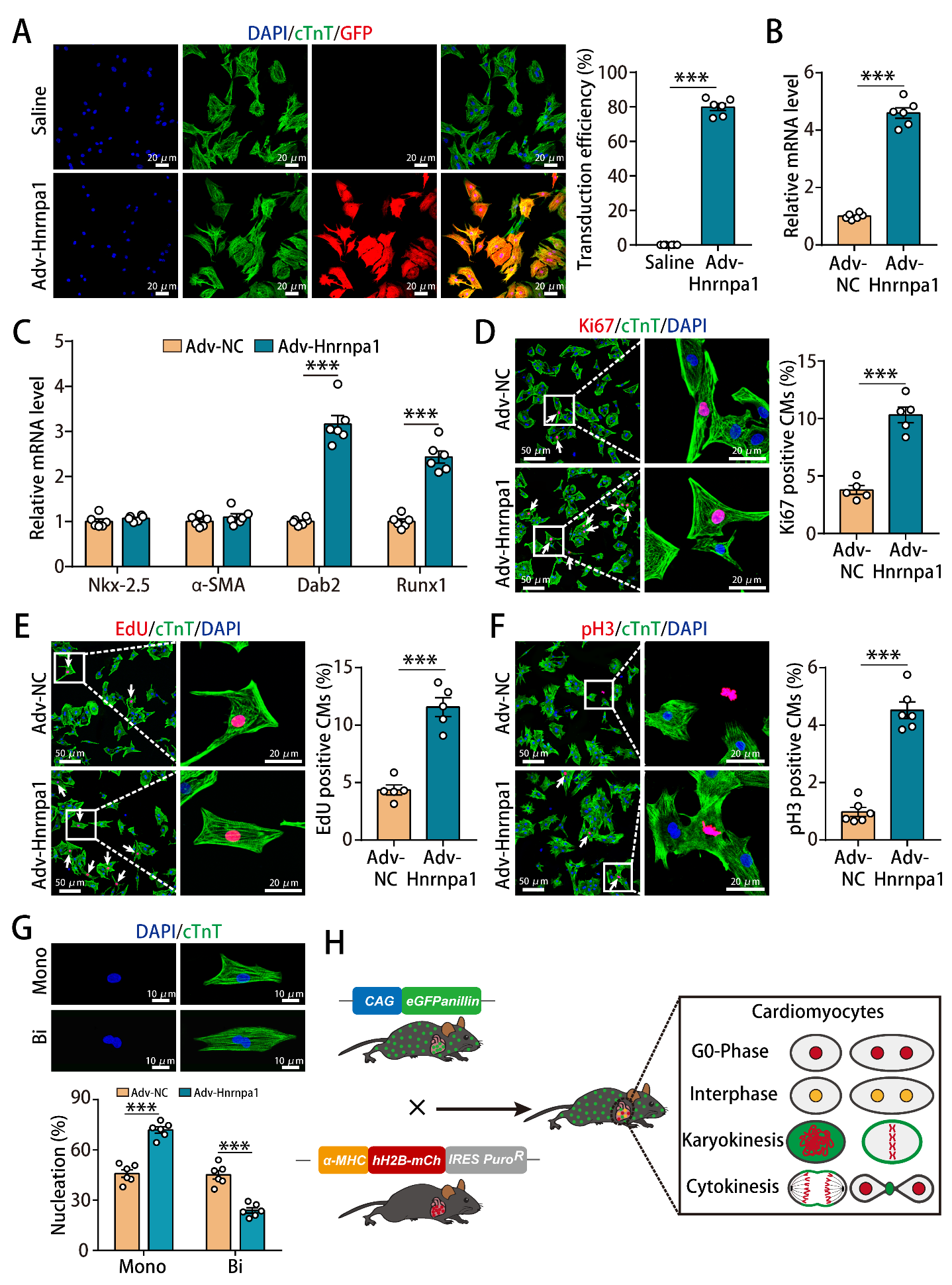


Supplemental Figure 4


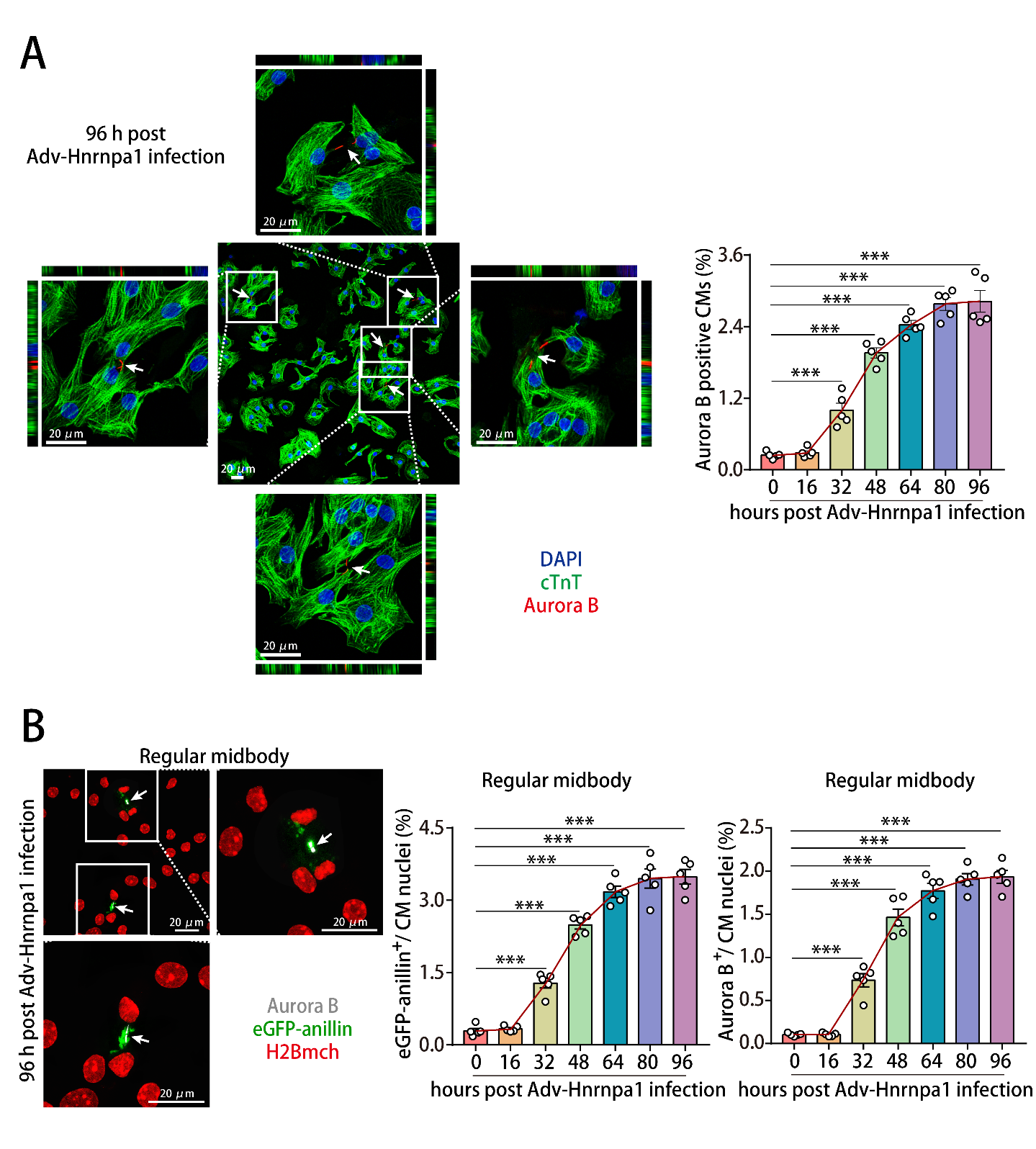


Supplemental Figure 5


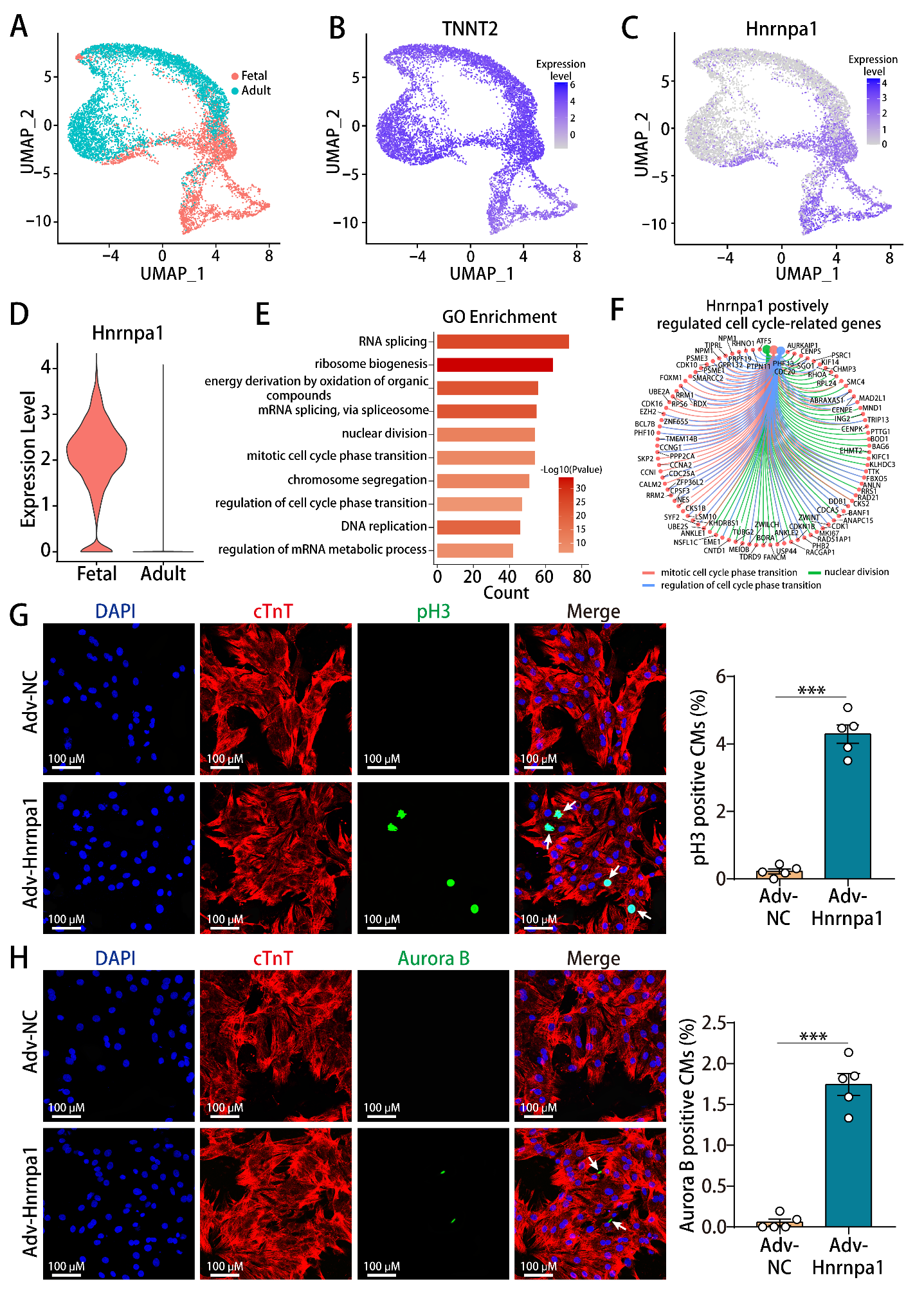


Supplemental Figure 6


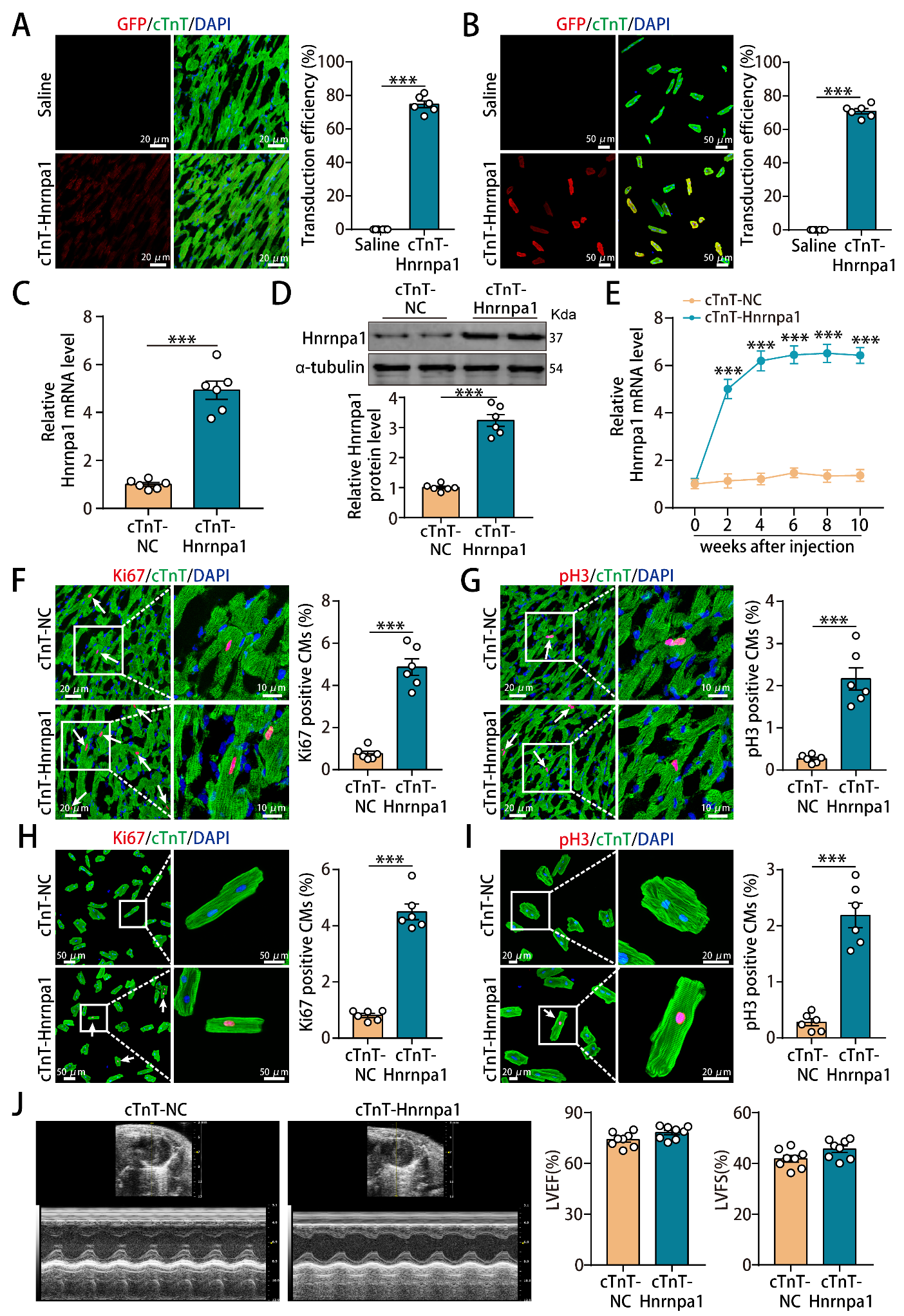


Supplemental Figure 7

**
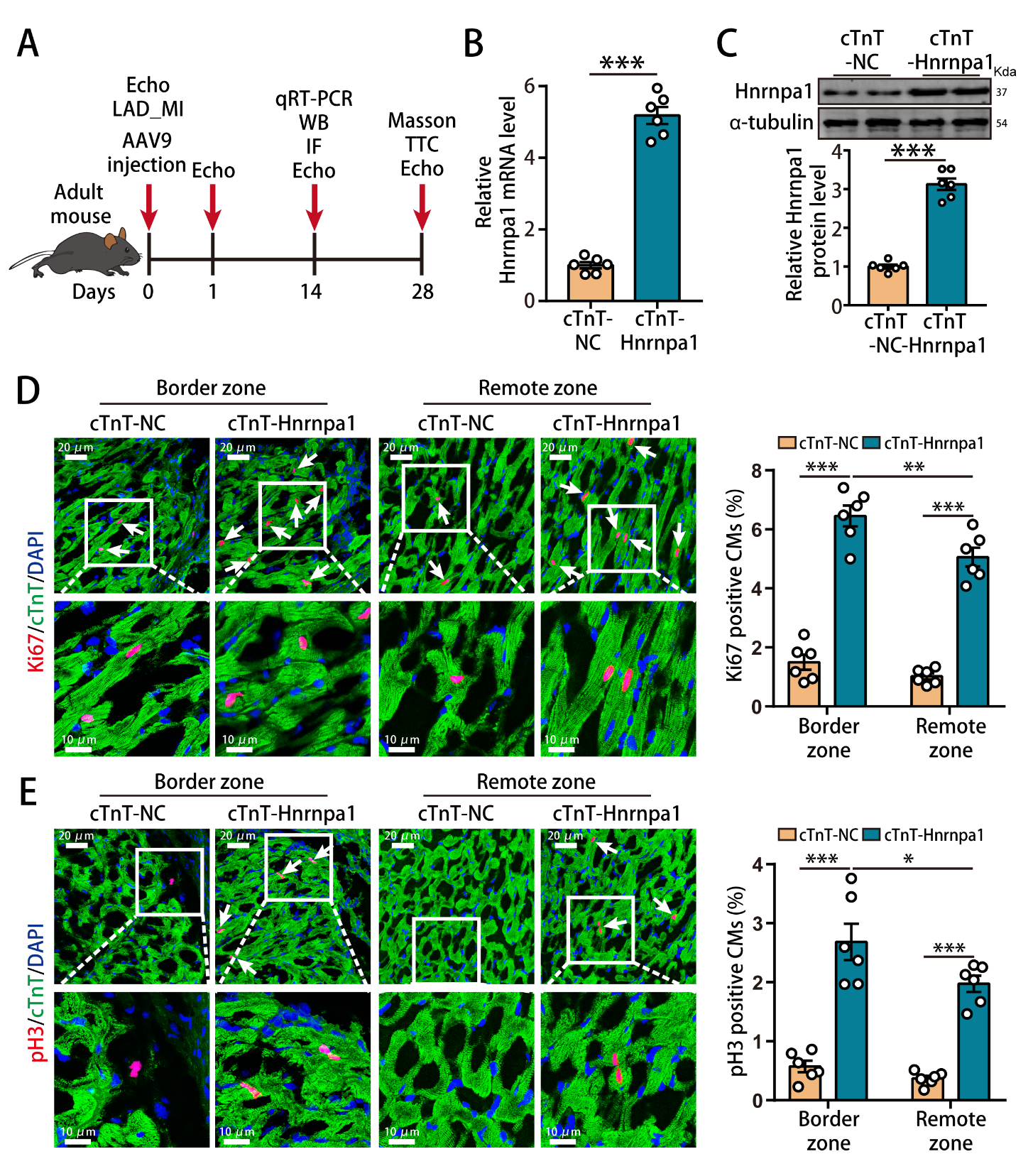
**

Supplemental Figure 8


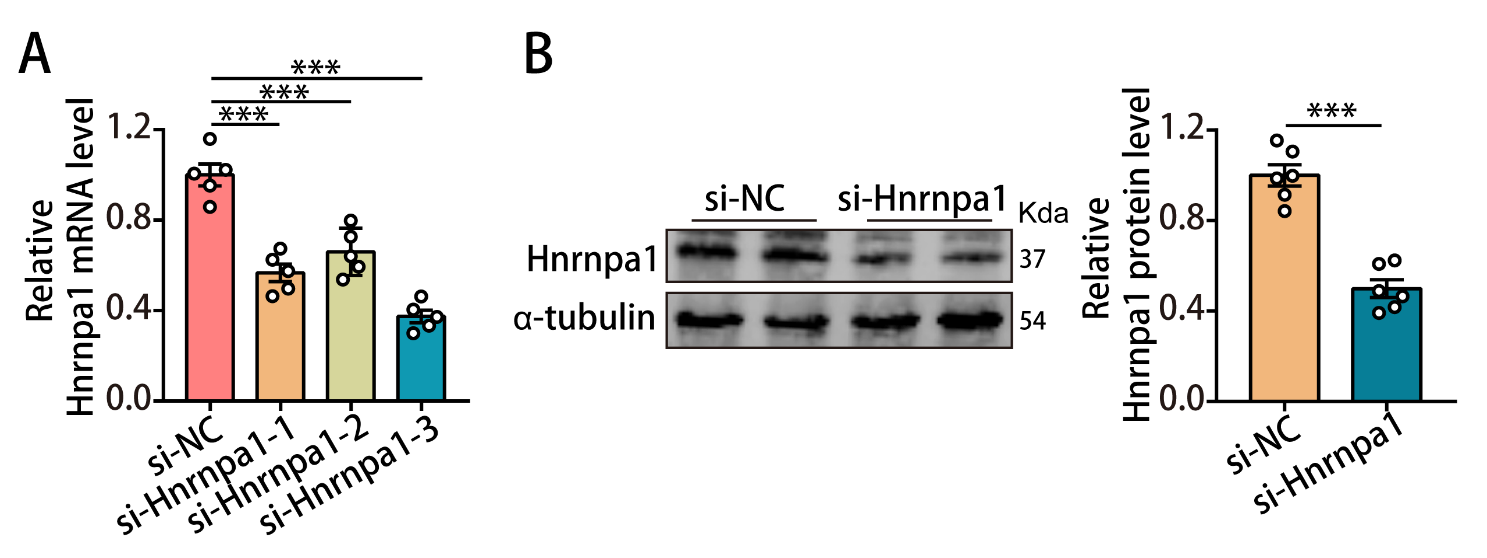


Supplemental Figure 9


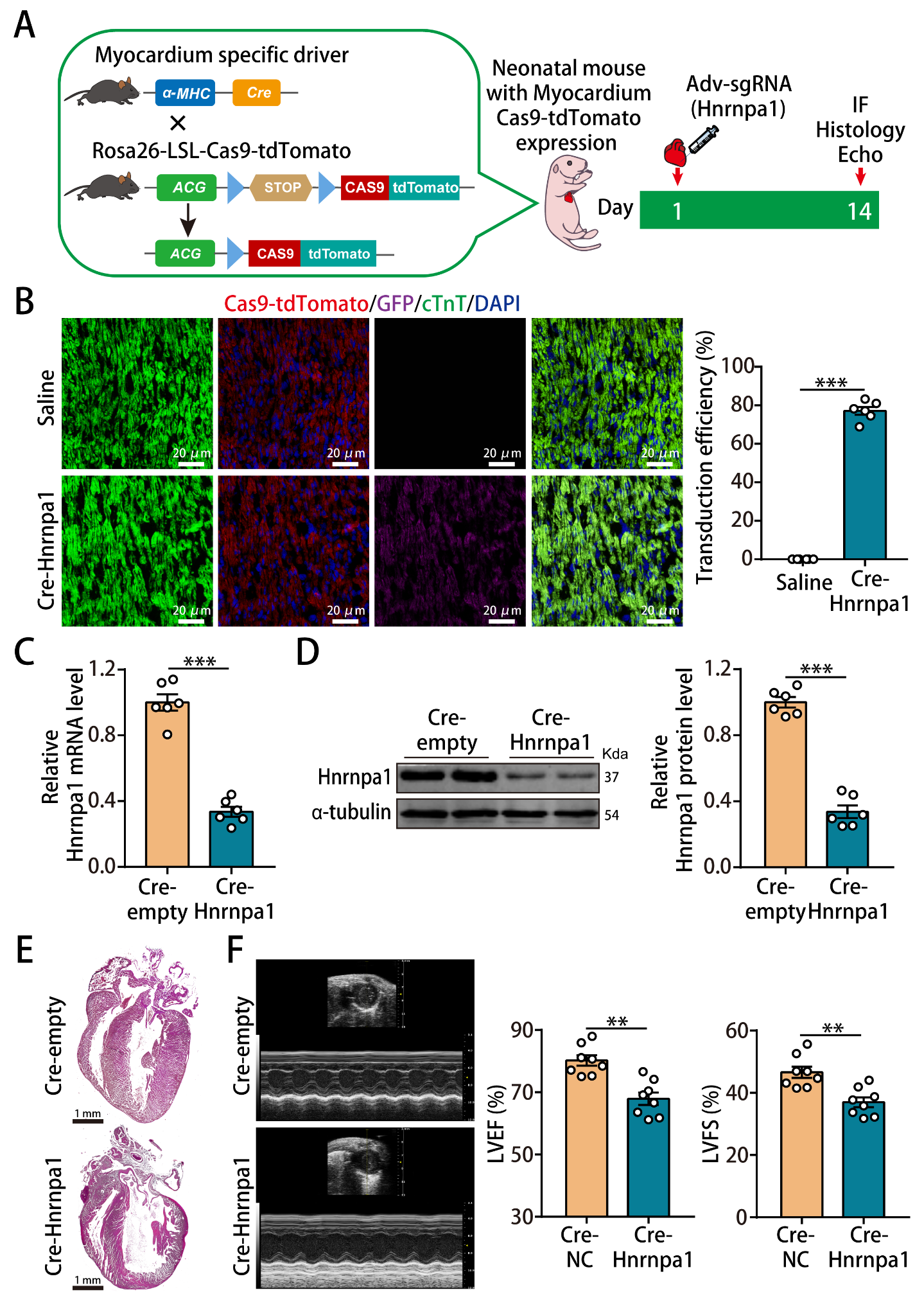


Supplemental Figure 10


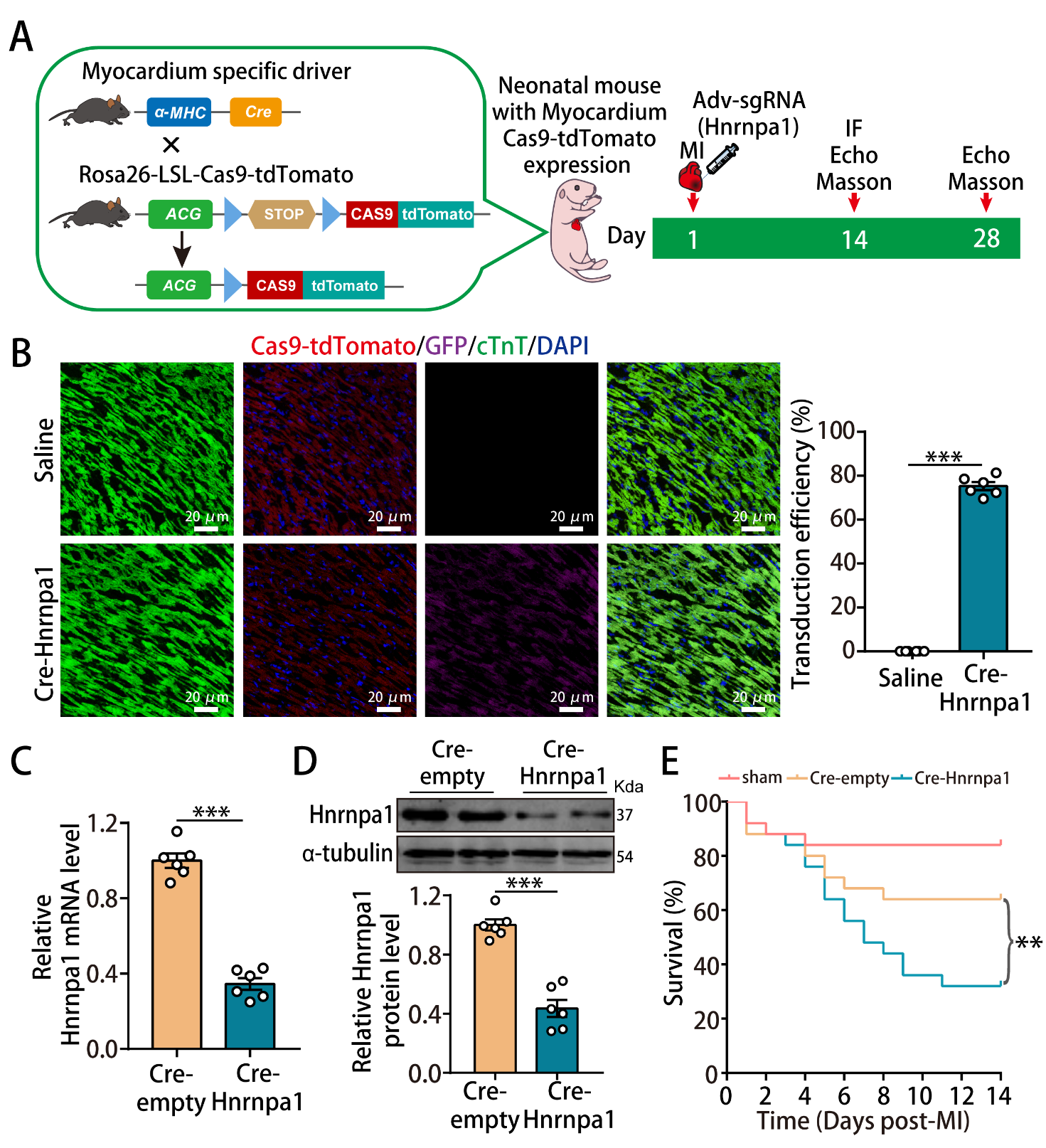


Supplemental Figure 11


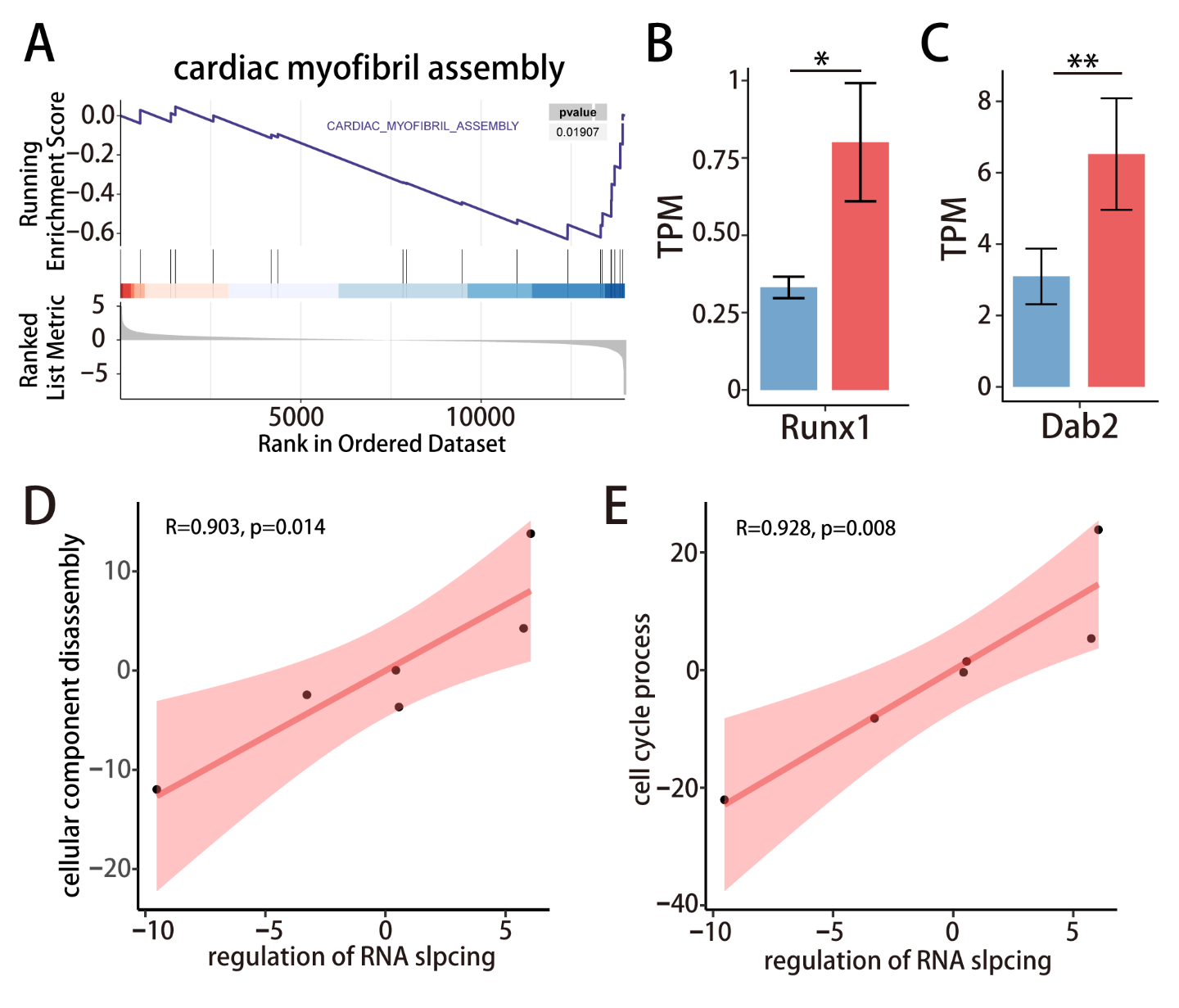


Supplemental Figure 12


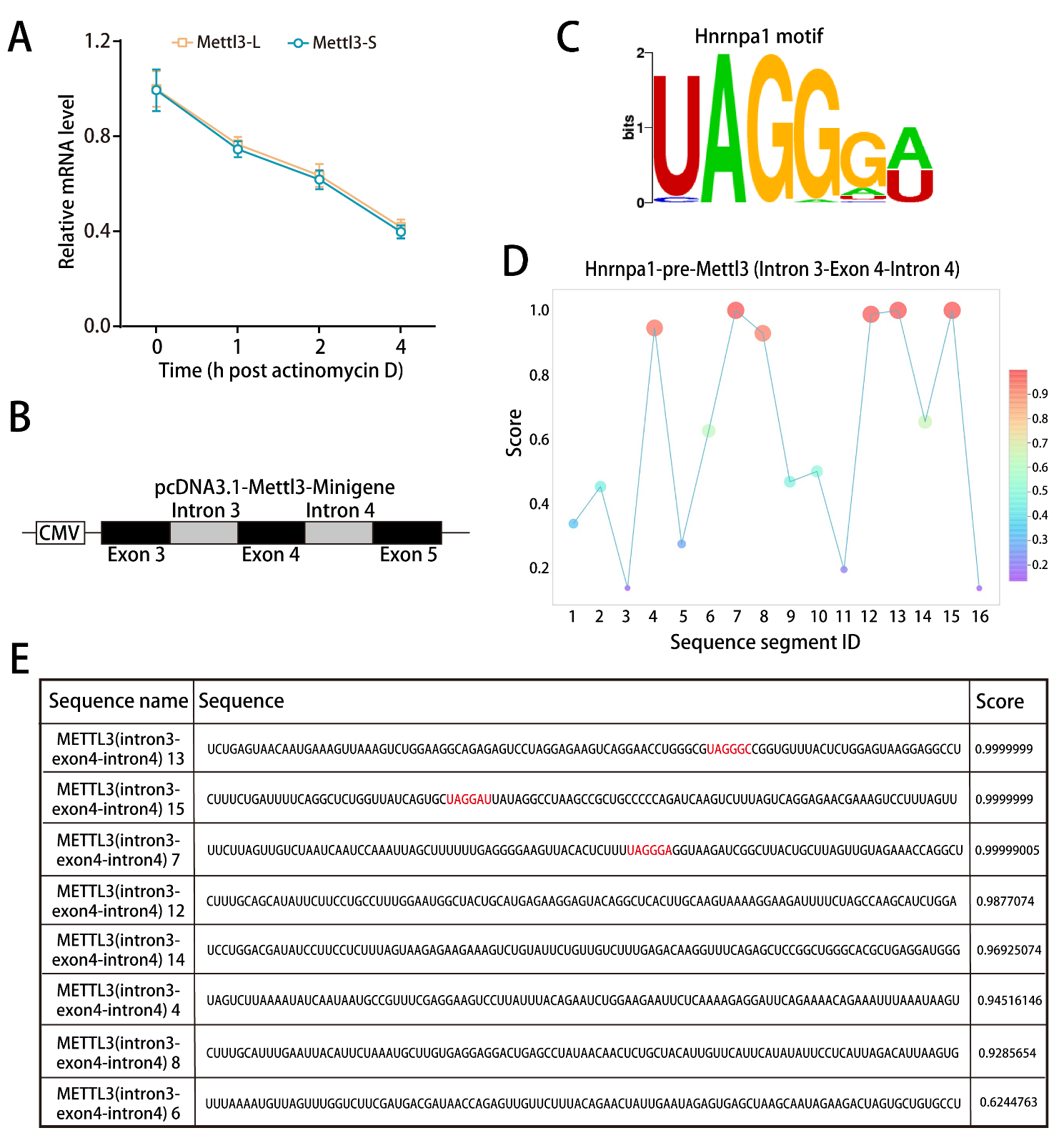


Supplemental Figure 13


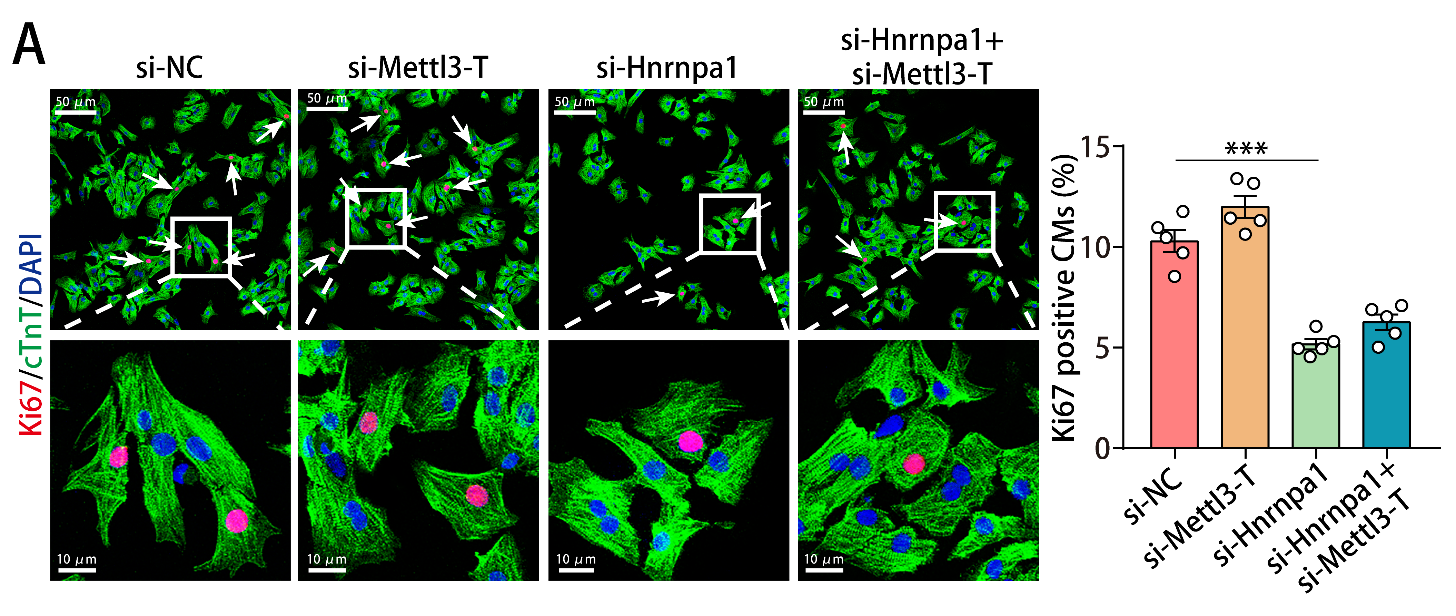


Supplemental Figure 14


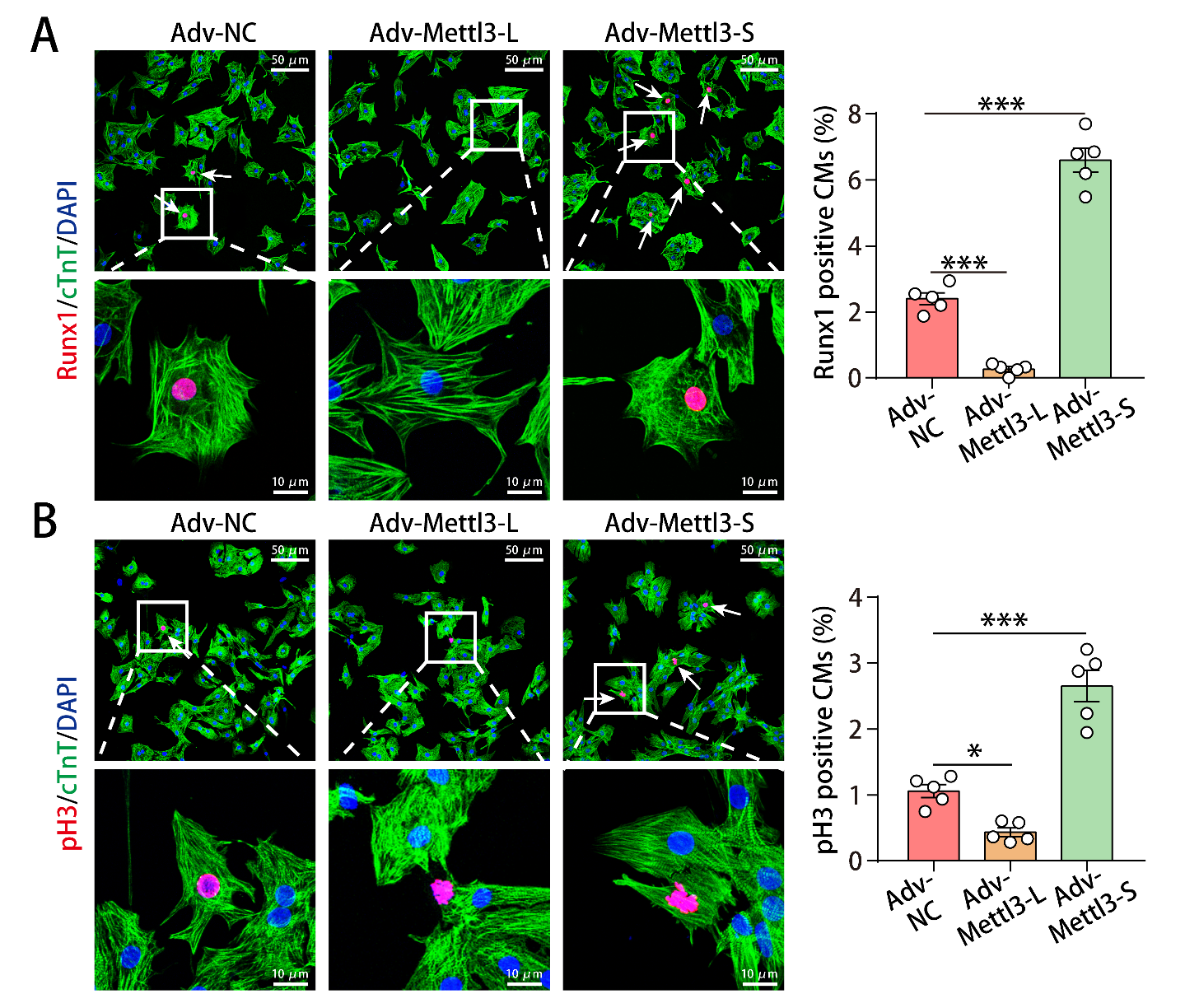


Supplemental Figure 15


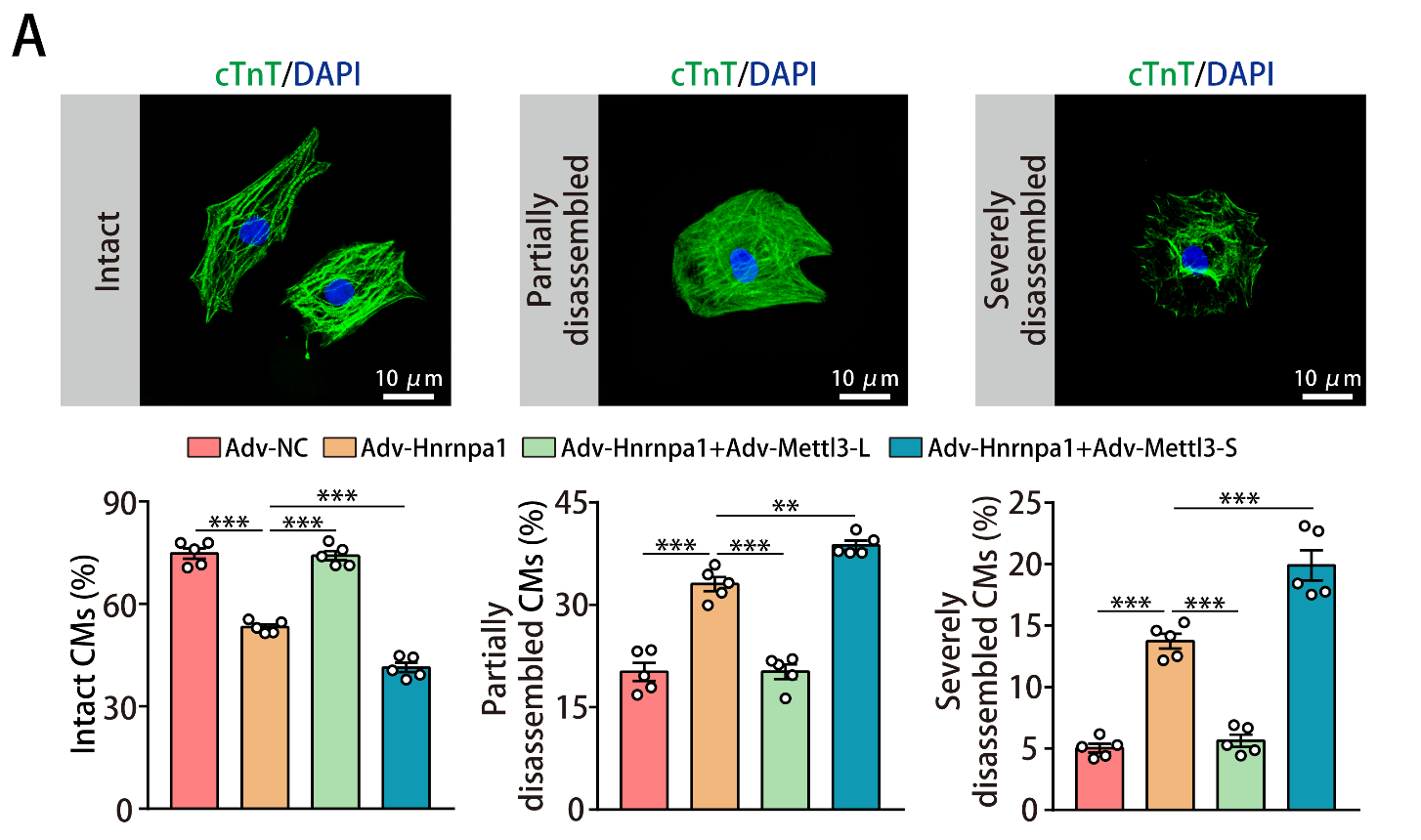


Supplemental Figure 16


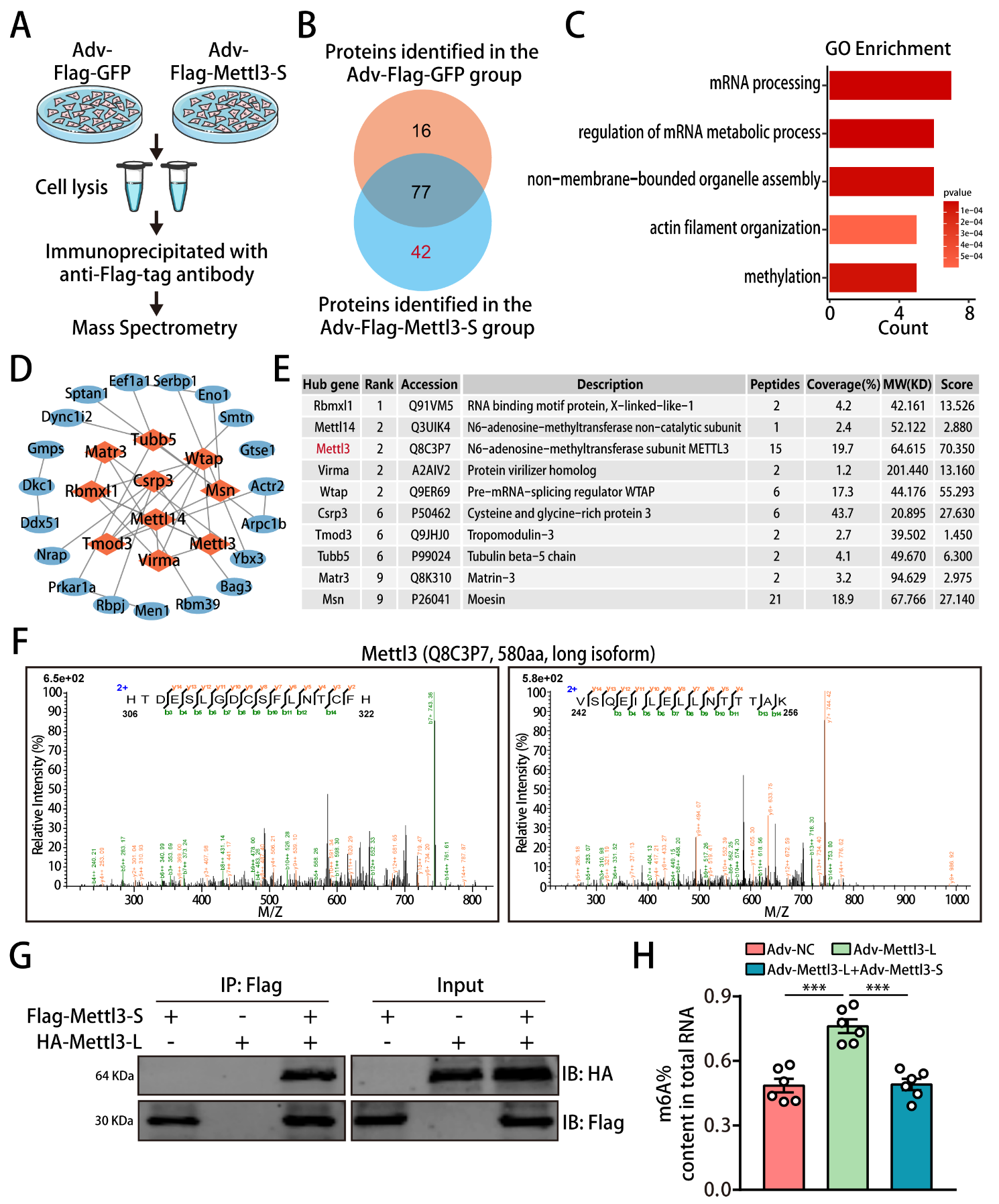


Supplemental Figure 17


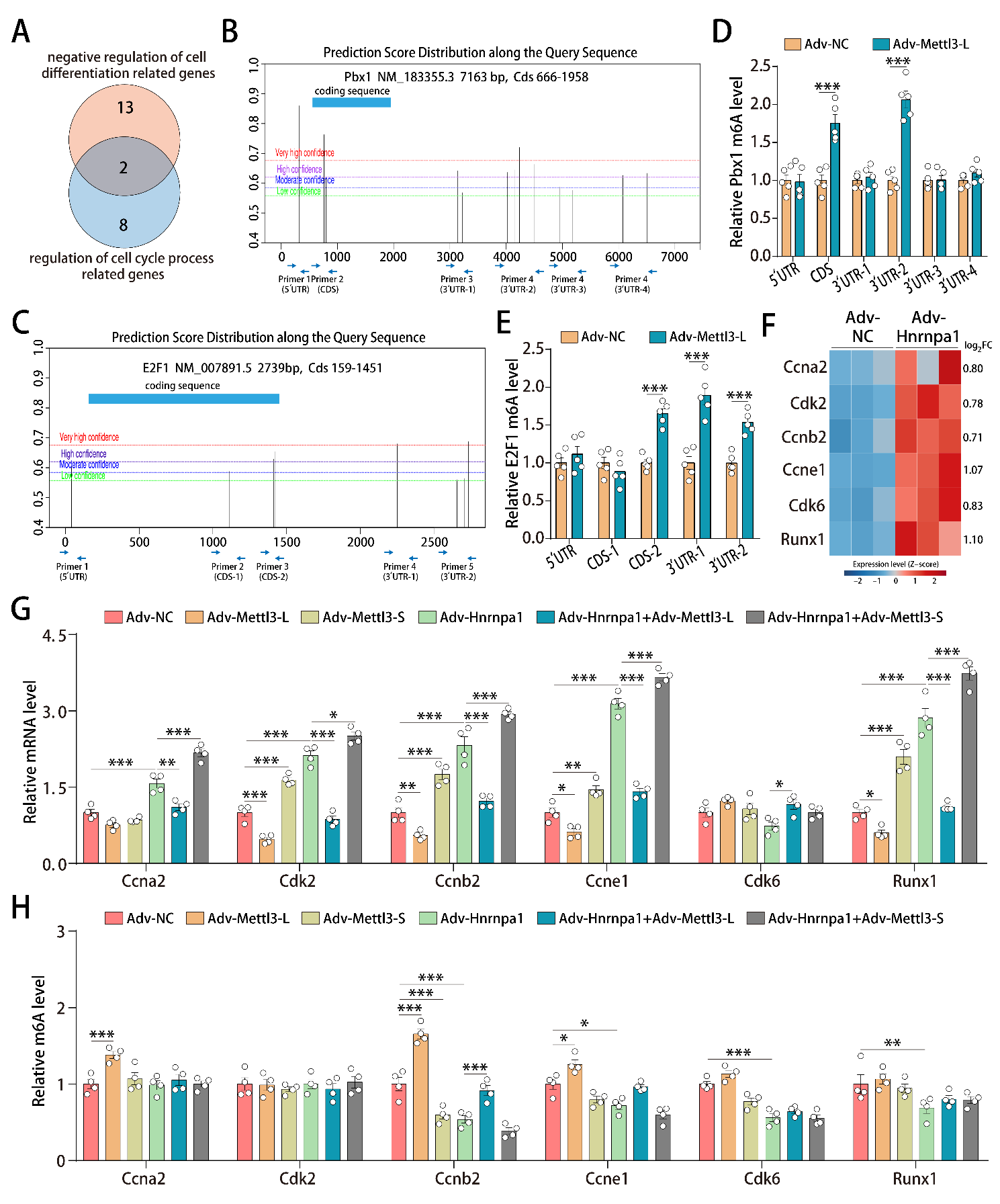


Supplemental Figure 18

**Supplementary Figure 1. Single nucleus RNA-seq analysis of hearts at different ages.**

**(A)** UMAP visualization of 14 cell clusters colored by identity in the heart of P2, P4, P9 and P11 mice (n=13367). **(B)** Dot plot showing cell-specific markers of CMs, CFs and ECs. **(C)** UMAP visualization of 5 cell clusters colored by identity in the heart of adult mice (n=8086). **(D)** UMAP visualization of 15 CM clusters colored by identity (n=13383). **(E)** Heatmap of Tnnt2 expression in each CM cluster projected on UMAP graph. **(F)** Violin plots showing the expression of Tnnt2 in 15 CM clusters. **(G)** Heatmap showing the expression of selected genes in 15 CM clusters. **(H)** Integration of 15 CM clusters into 6 CM clusters via gene expression correlation analysis. **(I)** The highly specific marker genes for each cluster. Gene signatures of cardiomyocyte CM1-CM6 populations were based on the expression levels of top marker genes for each cluster. **(J)** GO enrichment analysis of the highly expressed genes in CM1-6 clusters.

**Supplementary Figure 2. The cell transformation analysis of CM clusters.**

**(A)** Bar plots showing the distribution of CM clusters in state 1, 2, and 3. **(B-D)** UMAP visualization of CM clusters in state 1 (n=2050), state 2 (n=9008), and state 3 (n=2325) (left). RNA velocity analysis indicating altered trajectories for CMs across state 1-3. Local vectors demonstrated the cell fate trajectory from the early stage to the late stage, and the vector length coded the velocity of the transition (middle). Partition-based graph abstraction (PAGA) velocity graph illustrating trajectory inference of CM clusters across states 1-3 (right).

**Supplementary Figure 3. Identifying the crucial RBP with potential in regulating cardiomyocyte dedifferentiation and cell cycle activity.**

**(A)** Illustration of steps for identifying and validating crucial RBPs that are involved in CM dedifferentiation and cell cycle activity. **(B)** Time-series cluster analysis of the upregulated RBPs data in state 1 showed the different mRNA expression patterns during postnatal heart development. **(C)** Line plot depicting the percentage of CM4+CM5 in the heart of P2, P4, P9, P11 and adult mice. **(D-E)** Box plots illustrating the AUC scores of ‘regulation of the mitotic cell cycle’ or ‘cellular component disassembly’ in CMs from P2, P4, P9, P11 and adult mice. **(F)** The mRNA expression of ten core RBPs in mouse hearts of different ages. n=6 mice. **(G)** Npm1, Rps24, Ybx1, Rpl4, Snrpd1 and Hnrnpa1 mRNA expression in CMs isolated from mouse hearts at different ages. n=5 cell samples. **(H)** Npm1, Rps24, Ybx1, Snrpd1 and Hnrnpa1 mRNA expression in isolated CMs, CFs and ECs. n=5 cell samples. Statistical significance was calculated using one-way ANOVA in **F-H**; **P*<0.05, ***P*<0.01, ****P*<0.001.

**Supplementary Figure 4. Hnrnpa1 overexpression promoted P7 CM dedifferentiation and cell cycle activity.**

**(A)** GFP and cTnT co-staining determined the transduction efficiency of Adv-Hnrnpa1 in isolated P7 CMs (230 CMs from 6 mice in the Adv-NC group and 190 CMs from 6 mice in the Adv-Hnrnpa1 group). **(B)** Hnrnpa1 mRNA expression in isolated P7 CMs after Hnrnpa1 overexpression. n=6 cell samples. **(C)** The mRNA expression of dedifferentiation markers Nkx-2.5, α-SMA, Dab2 and Runx1 in P7 CMs after Hnrnpa1 overexpression. n=6 cell samples. **(D)** Ki67 staining of P7 CMs (701 CMs from 5 mice in the Adv-NC group and 741 CMs from 5 mice in the Adv-Hnrnpa1 group). **(E)** EdU staining of P7 CMs (692 CMs from 5 mice in the Adv-NC group and 802 CMs from 5 mice in the Adv-Hnrnpa1 group). **(F)** pH3 staining of P7 CMs (829 CMs from 6 mice in the Adv-NC group and 915 CMs from 6 mice in the Adv-Hnrnpa1 group). **(G)** Analysis of CM cell nucleation in isolated P7 CMs (669 CMs from 6 mice in the Adv-NC group and 1281 CMs from 6 mice in the Adv-Hnrnpa1 group). **(H)** Establishment about the α-MHC-H2B-mCh/CAG-eGFP-anillin system. Bars=20 µm in **A**, 50 µm (left) and 20 µm (right) in **D-F**, 10 µm in **G**. Statistical significance was calculated using an unpaired t test in **A-G**; **P*<0.05, ***P*<0.01, ****P*<0.001.

**Supplementary Figure 5. Growth curve and multiple time point evaluation in isolated P7 CMs post Adv-Hnrnpa1 infection.**

**(A)** Aurora B staining of P7 CMs after Hnrnpa1 overexpression (2129 CMs from 5 mice in the 0 h group, 2192 CMs from 5 mice in the 16 h group, 2781 CMs from 5 mice in the 32 h group, 2995 CMs from 5 mice in the 48 h group, 3231 CMs from 5 mice in the 64 h group, 3301 CMs from 5 mice in the 80 h group and 3266 CMs from 5 mice in the 96 h group). **(B)** Representative image of a P7 double transgenic CMs expressing eGFP-anillin (green) and Aurora B (white) after Hnrnpa1 overexpression and the frequency of regular midbodies identified by anillin or Aurora B staining (4919 CMs from 5 mice in the 0 h group, 4949 CMs from 5 mice in the 16 h group, 5214 CMs from 5 mice in the 32 h group, 5329 CMs from 5 mice in the 48 h group, 5542 CMs from 5 mice in the 64 h group, 5533 CMs from 5 mice in the 80 h group and 5388 CMs from 5 mice in the 96 h group). Bars=20 µm in **A-B**. Statistical significance was calculated using one-way ANOVA in **A-B**; **P*<0.05, ***P*<0.01, ****P*<0.001.

**Supplementary Figure 6. Hnrnpa1 overexpression induced cell cycle activation in hiPSC-CMs.**

**(A)** UMAP visualization of fetal and adult human CMs (n=8930). **(B)** Heatmap of Tnnt2 expression projected on the UMAP graph. **(C)** Heatmap of Hnrnpa1 expression projected on the UMAP graph. **(D)** Violin plots showing the expression of Hnrnpa1 in fetal and adult CMs. **(E)** GO enrichment analysis of Hnrnpa1 positively-correlated genes. The x-axis indicates the gene count and the y-axis specifies the GO terms. **(F)** Network plot illustrating the cell cycle-related GO terms of Hnrnpa1 positively-correlated genes. Red color is related to the mitotic cell cycle phase transition, green color is related to nuclear division, and blue color was related to the regulation of cell cycle phase transition. **(G)** pH3 staining of hiPSC-CMs after Hnrnpa1 overexpression (1981 CMs from 5 cell samples in the Adv-NC group and 2020 CMs from 5 cell samples in the Adv-Hnrnpa1 group). **(H)** Aurora B staining of hiPSC-CMs after Hnrnpa1 overexpression (3343 CMs from 5 cell samples in the Adv-NC group and 3466 CMs from 5 cell samples in the Adv-Hnrnpa1 group). Bars=100 µm in **G-H**. Statistical significance was calculated using an unpaired t test in **G-H**; **P*<0.05, ***P*<0.01, ****P*<0.001.

**Supplementary Figure 7. Hnrnpa1 overexpression promoted adult CM cell cycle activity in vivo.**

**(A)** The transduction efficiency of AAV9-cTnT-Hnrnpa1 in adult mouse hearts at 14 days after AAV9 infection. n=6 mice. **(B)** The transduction efficiency of AAV9-cTnT-Hnrnpa1 in adult CMs isolated from adult mouse hearts at 14 days after AAV9 infection (282 CMs from 6 mice in the Saline group and 275 CMs from 6 mice in the cTnT-Hnrnpa1 group). **(C-D)** Hnrnpa1 mRNA and protein expression in adult mouse hearts after Hnrnpa1 overexpression. n=6 mice. **(E)** The maintenance time for the AAV9 delivery system to overexpress Hnrnpa1 in adult mouse hearts. n=4 mice. **(F)** Ki67 staining of adult mouse hearts (984 CMs from 6 mice in the cTnT-NC group and 751 CMs from 6 mice in the cTnT-Hnrnpa1 group). **(G)** pH3 staining of adult mouse hearts (2586 CMs from 6 mice in the cTnT-NC group and 1182 CMs from 6 mice in the cTnT-Hnrnpa1 group). **(H)** Detection of Ki67^+^ adult CMs isolated from adult mouse hearts (1107 CMs from 6 mice in the cTnT-NC group and 859 CMs from 6 mice in the cTnT-Hnrnpa1 group). **(I)** Detection of pH3^+^ adult CMs isolated from adult mouse hearts (2973 CMs from 6 mice in the cTnT-NC group and 1219 CMs from 6 mice in the cTnT-Hnrnpa1 group). **(J)** Evaluation of cardiac function in normal adult mice at 14 days after AAV9 infection. n=8 mice. Bars=20 μm in **A and I**, 20 µm (left) and 10 µm (right) in **F-G,** 50 μm in **B** and **H**. Statistical significance was calculated using an unpaired t test in **A-D and F-J**, and two-way ANOVA in **E**; **P*<0.05, ***P*<0.01, ****P*<0.001.

**Supplementary Figure 8. Hnrnpa1 overexpression promoted adult cardiac regeneration after myocardial infarction.**

**(A)** Schematic diagram for the MI experimental procedure after AAV9 infection. Echo, echocardiography. **(B)** Hnrnpa1 mRNA expression in adult mouse MI model hearts 14 days after Hnrnpa1 overexpression. n=6 mice. **(C)** Hnrnpa1 protein levels in adult mouse MI model hearts 14 days after Hnrnpa1 overexpression. n=6 mice. **(D)** Ki67 staining of adult mouse hearts at 14 days post-MI (745 CMs from the border zone and 916 CMs from the remote zone of 6 mice in the cTnT-NC group; 831 CMs from the border zone and 829 CMs from the remote zone of 6 mice in the cTnT-Hnrnpa1 group). **(E)** pH3 staining of adult mouse hearts at 14 days post-MI (1265 CMs from the border zone and 2019 CMs from the remote zone of 6 mice in the cTnT-NC group; 1129 CMs from the border zone and 2155 CMs from the remote zone of 6 mice in the cTnT-Hnrnpa1 group). Bars=20 µm (upper) and 10 µm (lower) in **D-E**. Statistical significance was calculated using an unpaired t test in **B-C** and two-way ANOVA in **D-E**; **P*<0.05, ***P*<0.01, ****P*<0.001.

**Supplementary Figure 9. Efficiency of Hnrnpa1 knockdown in isolated P1 CMs.**

**(A)** Hnrnpa1 mRNA expression in P1 CMs transfected with si-NC or three Hnrnpa1 siRNAs. n=5 cell samples. **(B)** Hnrnpa1 protein levels in P1 CMs after Hnrnpa1 downregulation. n=6 cell samples. Statistical significance was calculated using one-way ANOVA in **A** and unpaired t test in **B**; **P*<0.05, ***P*<0.01, ****P*<0.001.

**Supplementary Figure 10. Hnrnpa1 deficiency impaired the cardiac function in normal neonatal mice.**

**(A)** Schematic diagram illustrating the crossbreeding of Cas9-tdTomato mice with α-MHC-Cre mice to obtain mice myocardially expressing Cas9, followed by injection of the Adv-mediated delivery system carrying sgRNA-Hnrnpa1. **(B)** GFP and cTnT co-staining of neonatal mice heart with myocardium Cas9-tdTomato expression at 14 days after Adv infection. n=6 mice. **(C-D)** Hnrnpa1 mRNA and protein expression in neonatal mouse hearts after Hnrnpa1 depletion. n=6 mice. **(E)** Cardiac structure of normal neonatal mice after Hnrnpa1 depletion. **(F)** Evaluation of cardiac function in normal neonatal mice at 14 days after Adv infection. n=8 mice. Bar=20 µm in **B** and 1mm in **E**. Statistical significance was calculated using an unpaired t test in **B-D** and **F**; **P*<0.05, ***P*<0.01, ****P*<0.001.

**Supplementary Figure 11. Hnrnpa1 deficiency led to a lower survival rate in neonatal mice after myocardial infarction.**

**(A)** Schematic diagram illustrating the crossbreeding of Cas9-tdTomato mice with α-MHC-Cre mice to obtain mice myocardially expressing Cas9, followed by LAD ligation and injection of the Adv-mediated delivery system carrying sgRNA-Hnrnpa1. **(B)** GFP and cTnT co-staining of neonatal mice heart with myocardium Cas9-tdTomato expression at 14 days after Adv infection. n=6 mice. **(C-D)** Hnrnpa1 mRNA expression and protein levels in neonatal mouse MI model hearts after Hnrnpa1 depletion. n=6 mice. **(E)** Analysis of the survival rate after Hnrnpa1 depletion. n=25 mice. Bar=20 µm in **B**. Statistical significance was calculated using an unpaired t test in **B-D** and the log-rank (Mantel-Cox) test in **E**; **P*<0.05, ***P*<0.01, ****P*<0.001.

**Supplementary Figure 12. Hnrnpa1 overexpression promoted cardiomyocyte dedifferentiation and cell cycle activity.**

**(A)** Gene Set Enrichment Analysis of cardiac myofibril assembly in P7 CMs after Hnrnpa1 overexpression. (**B-C)** The transcript expression of Runx1 and Dab2 in P7 CMs after Hnrnpa1 overexpression. TPM:Transcript per Million. n= 3 cell samples. (**D)** Z-score correlation analysis of the biological pathways ‘regulation of RNA splicing’ and ‘cellular component disassembly’. (**E**) Z-score correlation analysis of the biological pathways ‘regulation of RNA splicing’ and ‘cell cycle process’. **P*<0.05, ***P*<0.01, ****P*<0.001.

**Supplementary Figure 13. Hnrnpa1 binding site in the intron3-exon4-intron4 sequence of pre-Mettl3.**

**(A)** Mettl3-L and Mettl3-S mRNA levels in P7 CMs at different time points. Actinomycin D was used to block RNA synthesis. n=5 cell samples. **(B)** Graphical for pcDNA3.1-Mettl3-minigene containing the exon 3-intron 3-exon 4-intron 4-exon 5 sequence**. (C)** The known motif of Hnrnpa1 downloaded from the RBPsuite website (http://www. csbio.sjtu.edu.cn/bioinf/RBPsuite/). **(D-E)** The score of the Hnrnpa1 binding site in the intron 3-exon 4-intron 4 sequence of pre-Mettl3 downloaded from the RBPsuite website.

**Supplementary Figure 14.** **Mettl3 total mRNA expression has limited effect on Hnrnpa1-mediated CM function.**

**(A)** Ki67 staining of P1 CMs (886 CMs from 5 mice in the si-NC group, 956 CMs from 5 mice in the si-Mettl3-T group, 865 CMs from 5 mice in the si-Hnrnpa1 group and 856 CMs from 5 mice in the si-Hnrnpa1+si-Mettl3-T group). Mettl3-T indicates total Mettl3. Bars=50 µm (upper) and 10 µm (lower) in **A**. Statistical significance was calculated using one-way ANOVA in **A**; **P*<0.05, ***P*<0.01, ****P*<0.001.

**Supplementary Figure 15. The effect of different Mettl3 isoforms on cardiomyocyte dedifferentiation and cell cycle activity.**

**(A)** Runx1 staining of P7 CMs after Mettl3-L or Mettl3-S interference (1394 CMs of 5 mice in the Adv-NC group, 1683 CMs of 5 mice in the Adv-Mettl3-L group and 912 CMs of 5 mice in the Adv- Mettl3-S group). **(B)** pH3 staining of P7 CMs after Mettl3-L or Mettl3-S interference (848 CMs of 5 mice in the Adv-NC group, 1267 CMs of 5 mice in the Adv-Mettl3-L group and 860 CMs of 5 mice in the Adv-Mettl3-S group). Bars=50 µm (upper) and 10 µm (lower) in **A-B**. Statistical significance was calculated using one-way ANOVA in **A-B**; **P*<0.05, ***P*<0.01, ****P*<0.001.

**Supplementary Figure 16. Mettl3-L and Mettl3-S played opposite roles in Hnrnpa1-mediated CM myofibril disassembly.**

**(A)** cTnT staining of P7 CMs after Hnrnpa1, Mettl3-L and Mettl3-S interference (1060 CMs of 5 mice in the Adv-NC group, 1144 CMs of 5 mice in the Adv-Hnrnpa1 group, 1039 CMs of 5 mice in the Adv-Hnrnpa1+Adv-Mettl3-L group and 996 CMs of 5 mice in the Adv-Hnrnpa1+Adv-Mettl3-S group). Bar=10 µm. Statistical significance was calculated using one-way ANOVA in **A**; **P*<0.05, ***P*<0.01, ****P*<0.001.

**Supplementary Figure 17. Mettl3-S directly interacted with Mettl3-L and inhibited its methyltransferase activity.**

**(A)** Schematic diagram illustrating the identification of Mettl3-S interacting proteins through mass spectrometry. **(B)** An overlap was performed between pull-down proteins in Adv-Flag-GFP and Adv-Flag-Mettl3-S incubated P7 CMs lysates. **(C)** GO enrichment analysis of 42 Mettl3-S-interacting proteins in Adv-Flag-Mettl3-S incubated P7 CMs. **(D)** Protein-protein interaction analysis of 42 Mettl3-S-interacting proteins in Adv-Flag-Mettl3-S incubated P7 CMs. **(E)** Summary of the top 10 core Mettl3-S-interacting proteins identified by mass spectrometry. **(F)** Representative MS/MS spectra of the tryptic peptides from Mettl3-L: HTDESLGDCSFLNTCFH (left) and VSQEILELLNTTTAK (right). The b and y ions were indicated with green and orange colors, respectively. **(G)** Mettl3-S and Mettl3-L co-immunoprecipitation assays in P7 CMs transfected with Adv-Flag-Mettl3-S and Adv-HA-Mettl3-L individually or together. **(H)** The m6A modification of total RNA in P7 CMs after Mettl3-L and Mettl3-S interference. n=6 cell samples. Statistical significance was calculated using one-way ANOVA in **H**; **P*<0.05, ***P*<0.01, ****P*<0.001.

**Supplementary Figure 18. The effect of Hnrnpa1-induced Mettl3 post-transcriptional splicing on dedifferentiation and cell cycle-related genes.**

**(A)** Venn diagram analysis of the common genes involved in the negative regulation of cell differentiation and the regulation of cell cycle process. **(B-C)** Prediction scores of m6A modifications in the Pbx1 and E2F1 genes were calculated using the SRAMP algorithm. Arrows show the location of qPCR primers. **(D-E)** MeRIP-qPCR was used to determine the m6A enrichment levels of different Pbx1 or E2F1 regions in P7 CMs after Mettl3-L overexpression. n=5 cell samples. **(F)** Heatmap displaying the core downstream targets of Pbx1 and E2F1 involved in the dedifferentiation and cell cycle progression. **(G)** mRNA levels of Ccna2, Cdk2, Ccnb2, Ccne1, Cdk6 and Runx1 in P7 CMs after Hnrnpa1, Mettl3-L and Mettl3-S interference. n=4 cell samples. **(H)** The m6A enrichment of Ccna2, Cdk2, Ccnb2, Ccne1, Cdk6 and Runx1 mRNAs in P7 CMs after Hnrnpa1, Mettl3-L and Mettl3-S interference. n=4 cell samples. Statistical significance was calculated using an unpaired t test in **D-E** and one-way ANOVA in **G-H**; **P*<0.05, ***P*<0.01, ****P*<0.001.
