# Supplementary material for: RNA‐Binding Protein Hnrnpa1 Triggers Daughter Cardiomyocyte Formation by Promoting Cardiomyocyte Dedifferentiation and Cell Cycle Activity in a Post‐Transcriptional Manner
Source: Adv Sci (Weinh). 2024 Nov 19;12(2):2402371. doi: 10.1002/advs.202402371 (PMC11727271; doi:10.1002/advs.202402371)
Supplement: Supplementary file 3 — Supplemental Tables [file ADVS-12-2402371-s002.docx]

**Table S1: The rationale behind the selection of specific marker genes for** **cardiomyocyte subpopulations.**

| **Gene** | **Anno** | **CM** | **Article Title** | **PMID** | **Description** |
| --- | --- | --- | --- | --- | --- |
| Myh7 | immature | CM1 | Dynamic transcriptional responses to injury of regenerative and non-regenerative cardiomyocytes revealed by single-nucleus RNA sequencing | 32220304 | Compared to the other cardiomyocyte populations, CM4 cells express higher levels of markers of immature hearts, including **Tnni1, Myh7, and Actc1** (Ames et al., 2013; Cui et al., 2018; Taegtmeyer et al., 2010) (Figure 3C), and lower levels of maturation genes, including **Myh6, Ryr2**, and Cacna1c (Sun and Nunes, 2017; Taegtmeyer et al., 2010) (Figures 6D), suggesting that they are immature. |
| Tnni1 |  |  |  |  |  |
| Actc1 |  |  |  |  |  |
| Myh6 | mature | CM2 |  |  |  |
| Ryr2 |  |  |  |  |  |
| Cav1 | adhesion related | CM3 | Cardiomyocyte gene programs encoding morphological and functional signatures in cardiac hypertrophy and failure | 30375404 | Some genes thought to be specifically expressed in endothelial cells (e.g., **Cav1 and Pecam1**) or fibroblasts (e.g., **Dcn and Lum**) were clustered together with genes essential for transcription; Cav1 and Pecam1 were in G7 and Dcn and Lum were in G8 (Supplementary Fig. 2a). We used single-molecule RNA in situ hybridization (smFISH)16 to confirm that these genes are expressed in sham and TAC cardiomyocytes (Supplementary Fig. 4). A previous study of single-nucleus RNA-seq of cardiomyocytes also mentioned the presence of cardiomyocytes expressing endothelial marker genes9, consistent with our findings. |
| Pecam1 |  |  |  |  |  |
| Dcn |  |  |  |  |  |
| Lum |  |  |  |  |  |
| Mki67 | proliferation | CM4 | Cardiac cellular diversity and functionality in cardiac repair by single-cell transcriptomics | 37920179 | This study also entified a small population of presumably proliferating cardiomyocytes (pCMs) that expressed cell cycle genes (e.g., **Mki67, Cenpp**, and Kif15) |
| Cenpp |  |  |  |  |  |
| Dab2 | Dedifferentiation | CM5 | Dedifferentiation, Proliferation and Redifferentiation of Adult Mammalian Cardiomyocytes after Ischemic Injury | 28642276 | During this time, some ACMs were immuno-positive for the dedifferentiation markers **Runx1 and Dab2**. |
| Runx1 |  |  |  |  |  |
| Xirp2 | hypertrophy | CM6 | Modulation of Angiotensin II–Mediated Cardiac Remodeling by the MEF2A Target Gene Xirp2 | 20093629 | Mice harboring a hypomorphic **Xirp2** allele are viable but display cardiac hypertrophy. |
| Nppa |  |  |  |  | To further characterize the hypertrophic phenotype we examined the expression of hypertrophic marker genes by qRT-PCR. There was no significant change in expression of **atrial natriuretic factor (ANF), brain natriuretic peptide (BNP)**, or alpha myosin heavy chain (αMHC) genes |
| Nppb |  |  |  |  |  |

**Table S2: Sequences of primers used for qRT-PCR and m6A-RIP-qPCR**

**in this study.**

| **Primers name** |  | **Sequence (5’-3’)** |
| --- | --- | --- |
| Hnrnpa1 | + | GACAGGGTTATGGAAACCAGG |
|  | - | GCCTCCTCCGTTGTTATAGCTG |
| Npm1 | + | CACCAGTTGTCATTAAGAACGGT |
|  | - | GACTGCCTTCATAGTTCATTGCT |
| Rps24 | + | AACCGTCTGCTTCAGAGGAAA |
|  | - | CAGTCTGTGTTTAGGCTCATTCT |
| Rps3 | + | ATGGCGGTGCAGATTTCCAA |
|  | - | GTAACTCGGACTTCAACTCCAG |
| Ybx1 | + | AAGGTCATCGCAACGAAGGTT |
|  | - | CAAATACGTCTTCCTTGGTGTCA |
| Snrpe | + | GGTGCAGCCCATCAACCTTAT |
|  | - | ATTCACTTGTTCATACAGCCACA |
| Rpl4 | + | CAGACCAGTGCTGAGTCTTGG |
|  | - | TTGGGTTGTATTCACTCTGCG |
| Snrpd1 | + | CAGCATGAACACACACCTTAAAG |
|  | - | GCCTCGAATACTCAATGTTTCCA |
| Pabpc1 | + | CCTCCTTCAGGTTACTTCATGGC |
|  | - | GGACTTGGTCTTAGTTGAGCAAT |
| Eif4a1 | + | ATGTCTGCGAGTCAGGATTCT |
|  | - | TCTCGTTCCAGTTACTCTCGAT |
| Nkx-2.5 | + | GACAAAGCCGAGACGGATGG |
|  | - | CTGTCGCTTGCACTTGTAGC |
| α-SMA | + | CCCAGACATCAGGGAGTAATGG |
|  | - | TCTATCGGATACTTCAGCGTCA |
| Runx1 | + | GAGATTCAACGACCTCAGGTTT |
|  | - | \| TGTAAAGACGGTGATGGTCAGA \| \| --- \| |
| Dab2 | + | CCTTCATTGCTCGTGATGTGA |
|  | - | CCCCAAACAAATCCATCTGGTC |
| Mettl3-Total | + | GAGGTAAAGCGAGGTCTCC |
|  | - | CCTTAGCTGGTTCTGATGC |
| Mettl3-L | + | GAACCAACAGTCAACGAA |
|  | - | TCAGAATCAACACAAGCA |
| Mettl3-S | + | CAAGAAGACGAATTATCAATAAGCA |
|  | - | TCTCAGAATCAACACAAGCA |
| E2F1 | + | CTGCTGACTCACTCCTG |
|  | - | GATCGTGCTATTCCAAT |
| Pbx1 | + | TGGTTTGGAAATAAGCGA |
|  | - | GAGTTGGGAGTAGAGGGC |
| Ccna2 | + | GCCTTCACCATTCATGTGGAT |
|  | - | TTGCTCCGGGTAAAGAGACAG |
| Cdk2 | + | CTCGACACTGAGACTGAAGGT |
|  | - | GCAGCTTGACGATATTAGGGTGA |
| Ccnb2 | + | GCCAAGAGCCATGTGACTATC |
|  | - | CAGAGCTGGTACTTTGGTGTTC |
| Ccne1 | + | CTCCGACCTTTCAGTCCGC |
|  | - | CACAGTCTTGTCAATCTTGGCA |
| Cdk6 | + | TCTCACAGAGTAGTGCATCGT |
|  | - | CGAGGTAAGGGCCATCTGAAAA |
| Gapdh | + | TGACCTCAACTACATGGTCTACA |
|  | - | CTTCCCATTCTCGGCCTTG |

**Table S3: Antibodies used for western blot in this study.**

|  | **Vendor or**  **Source** | **Catalog #** | **Dilute**  **Proportion** |
| --- | --- | --- | --- |
| anti-Hnrnpa1  anti-Hnrnpa1 | Santa Cruz Biotechnology  Proteintech | sc-32301  11176-1-AP | 1:100  1:2000 |
| anti-α-tubulin | Proteintech | 11224-1-AP | 1:3000 |
| anti-Dab2 | Santa Cruz Biotechnology | sc-136964 | 1:100 |
| anti-Runx1  anti-Mettl3-L  anti-Mettl3 | Santa Cruz Biotechnology  Proteintech  Abcam | sc-365644  15073-1-AP  ab240595 | 1:100  1:500  1:1000 |
| anti-Pbx1  anti-E2F1 | Proteintech  Santa Cruz Biotechnology | 18204-1-AP  sc-251 | 1:500  1:100 |
| anti-β-actin | Proteintech | 81115-1-RR | 1:5000 |
| anti-Ccne1 | Abcam | ab33911 | 1:1000 |
| anti-Cdk2 | Proteintech | 10122-1-AP | 1:1000 |
| anti-Ccnb2 | Proteintech | 21644-1-AP | 1:1000 |
| donkey anti-rabbit IgG H&L | Abcam | ab175772 | 1:10000 |
| Goat Anti-Mouse IgG H&L | Abcam | ab6708 | 1:10000 |

**Table S4: Antibodies used for Immunofluorescence staining in this**

**study**

|  | **Vendor or**  **Source** | **Catalog #** | **Dilute**  **Proportion** |
| --- | --- | --- | --- |
| anti- cTnT  anti- cTnT  anti-Hnrnpa1 | Santa Cruz Biotechnology  Abcam  Proteintech | sc-20025  ab209813  11176-1-AP | 1:50  1:100  1:100 |
| anti-PCM1 | Proteintech | 19856-1-AP | 1:50 |
| anti-ki67 | Abcam | ab15580 | 1:100 |
| anti-pH3 | Abcam | ab267372 | 1:100 |
| anti-aurora B | Abcam | ab2254 | 1:100 |
| anti-Dab2  anti-Runx1 | Santa Cruz Biotechnology  Santa Cruz Biotechnology | sc-136964  sc-365644 | 1:50  1:50 |
| goat anti-mouse IgG/Alexa Fluor 488 | Bioss | bs-0296G-AF488 | 1:100 |
| goat anti-rabbit IgG/Alexa Fluor 555 | Bioss | bs-0295G-AF555 | 1:100 |
| goat anti-mouse IgG/Alexa Fluor 647 | Bioss | bs-0296G-AF647 | 1:100 |
| goat anti-rabbit IgG/Alexa Fluor 647 | Bioss | bs-0295G-AF647 | 1:100 |

**Table S5: The mean Q score of the FASTQ files from the Nanopore RNA-seq**

**of P7 CMs.**

| **Group** | **Adv-NC-1** | **Adv-NC-2** | **Adv-NC-3** | **Adv-Hnrnpa1-1** | **Adv-Hnrnpa1-2** | **Adv-Hnrnpa1-3** |
| --- | --- | --- | --- | --- | --- | --- |
| **mean Q score** | 11 | 11 | 11 | 11 | 11 | 11 |

**Table S6: Sequences of primers used for semi-quantitative RT-PCR in**

**this study.**

| **Primers name** |  | **Sequence (5’-3’)** |
| --- | --- | --- |
| Mettl3-L | + | GCTCTTTAGCATCTGGTC |
|  | - | TTGGCTGTTGTGGTATTT |
| Mettl3-S | + | CAAGAAGACGAATTATCAATAAGCA |
|  | - | TCTCAGAATCAACACAAGCA |
| Gapdh | + | CCTCAACTACATGGTCTACA |
|  | - | CTTCCCATTCTCGGCCTTG |

**Table S7: Sequences of primers used for detecting the m6A enrichment levels of different Pbx1 regions in this study.**

| **Primers name** |  | **Sequence (5’-3’)** |
| --- | --- | --- |
| 5’UTR | + | CCCGATCAATGCATATTTGCA |
|  | - | GATAAGGGCTGGTGGGGAAGG |
| CDS | + | CGAAGAGGAAGAGCCGGGGCT |
|  | - | TGGTCATAATTTGCTGTAAAA |
| 3’UTR-1 | + | AGCCCAAGAGAGGAGCAGTA |
|  | - | AAAGCATCCAAATCCATCAT |
| 3’UTR-2 | + | TTGAATCCAGTTATGAGACC |
|  | - | AGAGAGAAGCATTCTGTGAG |
| 3’UTR-3 | + | CCTGAGAGATGGTAGCAGAC |
|  | - | TCGCAAGAAACAATGGAAAA |
| 3’UTR-4 | + | CCTCATGTCCCTGCTTTCTT |
|  | - | TACTCACGCTTGTCTTCTTT |

**Table S8: Sequences of primers used for detecting the m6A enrichment levels of different E2F1 regions in this study.**

| **Primers name** |  | **Sequence (5’-3’)** |
| --- | --- | --- |
| 5’UTR | + | GCGGCAAAAAGGATTTGG |
|  | - | GGGCCCATGACTGGCAGG |
| CDS-1 | + | GGAGGAGAGTGCAGACGG |
|  | - | TGGAGGGAGGTGATGGTG |
| CDS-2 | + | GGGGAGTTCATCAGCCT |
|  | - | AATCCAGAGGGGTCAGG |
| 3’UTR-1 | + | CCCAAGCAGTTTATTTATT |
|  | - | CTCACGTACCCTCCCCATC |
| 3’UTR-2 | + | ATTTCCTTTCAGTTTGTAA |
|  | - | ACAGTTCACTGTGTTGCAT |
